# Supplementary material for: SUPERGNOVA: local genetic correlation analysis reveals heterogeneous etiologic sharing of complex traits
Source: Genome Biol. 2021 Sep 7;22:262. doi: 10.1186/s13059-021-02478-w (PMC8422619; doi:10.1186/s13059-021-02478-w)
Supplement: Supplementary file 1 — Additional file 1. Supplementary note, containing further derivations of the statistical models, information about additional simulations, and discussion of interpretability of local genetic covariance. [file 13059_2021_2478_MOESM1_ESM.pdf]

Supplementary Note

1 Properties for the SUPERGNOVA framework

1.1 Statistical model for global genetic covariance

We follow the random design and random effects model to construct phenotypes, which is same as the model used by LDSC[2]. Suppose we sample two cohorts of different phenotypes, in which sample sizes are n_1 and n_2, respectively. Assume the two GWAS studies share the same set of SNPs and there are m SNPs in total. We measure phenotype 1 in cohort 1 and phenotype 2 in cohort 2. We assume all the m SNPs are associated with both traits. We model phenotype vectors for each cohort as

$$\phi_1 = X\beta + \epsilon$$
$$\phi_2 = Y\gamma + \delta,$$

where X and Y are standardized random matrices of genotypes, with demensions $n_1 \times m$ and $n_2 \times m$; β and γ are vectors of standardized genotype effect sizes, and δ and ϵ are vectors of residuals, representing environmental effects and non-additive genetic effects.

Each row of X and Y represents the standardized genotypes of an individual in the corresponding GWAS study. By standardized genotypes, we mean the genotype of each SNP is normalized to mean zero and variance one. We assume the genotypes of different samples are independent from each other. Due to linkage disequilibrium(LD), genotypes of different SNPs are correlated. We denote the LD matrix as V. That is, $cov(X_{i_1 \cdot}) = V = cov(Y_{i_2 \cdot})$, for any $1 \leq i_1 \leq n_1$ and $1 \leq i_2 \leq n_2$. We define the LD score of a variant j as $l_j := \sum_k V_{jk}^2$. We assume we can bound l_j by generic constant M, i.e. $M > l_j$, for $j \in \{1, 2, \ldots, p\}$. We suppose that $(\beta^T, \gamma^T)^T$ is subject to multivariate normal distribution and has mean zero and covariance matrix

$$\mathrm{Var}\left[\begin{pmatrix}\beta \\ \gamma\end{pmatrix}\right] = \frac{1}{m}\begin{pmatrix} h_1^2 I_m & \rho I_m \\ \rho I_m & h_2^2 I_m \end{pmatrix}.$$

We define h_1^2 and h_2^2 as the heritability of trait 1 and trait 2, respectively. ρ is defined as genetic covariance between trait 1 and trait 2. In addition, genetic correlation r is defined by $\rho/\sqrt{h_1^2 h_2^2}$.

In practice, two different GWASs often share a subset of samples. Without loss of generality, we assume $0 \leq n_s \leq \min\{n_1, n_2\}$ is the number of samples shared by the two GWASs and the first n_s samples in each study are shared, i.e. the first n_s rows of X and Y are the same. To account for the non-genetic correlation introduced by sample overlapping, we assume ϵ and δ are subject to multivariate normal distribution with covariance:

$$\mathrm{Cov}\left[\epsilon_i, \delta_j\right] = \begin{cases} \rho_e, 1 \leq i = j \leq n_s \\ 0, \text{otherwise} \end{cases}.$$

The variance of ϵ and δ are:

$$\mathrm{Var}\left[\epsilon\right] = \left(1 - h_1^2\right) I_{n_1},\ \mathrm{Var}\left[\delta\right] = \left(1 - h_2^2\right) I_{n_2},$$

so that

$$
\begin{aligned}
\text{Var}\left[\phi_1\right] &= \text{Var}\left[X\beta\right] + \text{Var}[\epsilon] \\
&= \mathbb{E}\left[X\beta\beta^T X^T\right] + \left(1 - h_1^2\right) I_{n_1} \\
&= \frac{h_1^2}{m}\mathbb{E}\left[XX^T\right] + \left(1 - h_1^2\right) I_{n_1} \\
&= h_1^2 I_{n_1} + \left(1 - h_1^2\right) I_{n_1} = I_{n_1}
\end{aligned}
$$

and similarly, $\text{Var}\left[\phi_2\right] = I_{n_2}$. We assume genotype, effect size, and environmental effect are independent to each other.

Genetic covariance ρ is the covariance of genetic components. For an individual with standarized genotype G, denoting g_1 and g_2 as the genetic components for trait 1 and trait 2, respectively, we have:

$$
\begin{aligned}
\text{Cov}\left[g_1, g_2\right] &= \mathbb{E}\left[G^T\beta\gamma^T G\right] \\
&= \mathbb{E}\left[\mathbb{E}\left[G^T\beta\gamma^T G \middle| G\right]\right] \\
&= \mathbb{E}\left[G^T\mathbb{E}\left[\beta\gamma^T\right] G\right] \\
&= \frac{\rho}{m}\mathbb{E}\left[G^T G\right] \\
&= \rho.
\end{aligned}
$$

We define genetic correlation as the genetic covariance normalized by the heritability:

$$
r_g = \frac{\text{Cov}\left[g_1, g_2\right]}{\sqrt{\text{Var}\left[g_1\right]\text{Var}\left[g_2\right]}} = \frac{\rho}{\sqrt{h_1^2 h_2^2}}.
$$

1.2 Statistical model for local genetic covariance

In this section, we generalize the statistical framework above for local genetic covariance. We assume ϕ_1 and ϕ_2 follow additive linear models:

$$
\begin{aligned}
\phi_1 &= \sum_{i=1}^{I} X_i\beta_i + \epsilon \\
\phi_2 &= \sum_{i=1}^{I} Y_i\gamma_i + \delta,
\end{aligned}
$$

where X_i and Y_i are the standarized genotypes and β_i and γ_i are the effect sizes of SNPs in regions i. We assume SNPs from different regions are independent and we use V_i to denote the LD matrix in region i. We assume effect size $(\beta_i^T, \gamma_i^T)^T$ is subject to multivariate normal distribution:

$$
\begin{pmatrix} \beta_i \\ \gamma_i \end{pmatrix} \sim N\left(0, \frac{1}{m_i}\begin{bmatrix} h_{1i}^2 I_{m_i} & \rho_i I_{m_i} \\ \rho_i I_{m_i} & h_{2i}^2 I_{m_i} \end{bmatrix}\right)
$$

and effect sizes of SNPs from different regions are independent. Environmental error covariance ρ_e in local genetic covariance is defined the same as it is in global genetic covariance. The variance of ϵ and δ are $Var\left[\epsilon\right] = \left(1 - \sum_{i=1}^{I} h_{1i}^2\right) I_{n_1}$and $Var\left[\delta\right] = \left(1 - \sum_{i=1}^{I} h_{2i}^2\right) I_{n_2}$ so that $Var\left(\phi_1\right) = 1$ and $Var\left(\phi_2\right) = 1$.

The covariance of the genetic components is the sum of local genetic covariance. Denote G_i

as the standardized genotype in region i for an individual. We have

$$\begin{aligned}\mathrm{Cov}\left[g_1, g_2\right] &= \mathbb{E}\left[\left(\sum_{i=1}^{I} G_i^T \beta_i\right)\left(\sum_{i=1}^{I} \gamma_i^T G_i\right)\right] \\ &= \sum_{i=1}^{I} \mathbb{E}\left[\left(G_i^T \beta_i \gamma_i^T G_i\right)\right] \\ &= \sum_{i=1}^{I} \mathbb{E}\left[\mathbb{E}\left[G_i^T \beta_i \gamma_i^T G_i \middle| G_i\right]\right] \\ &= \sum_{i=1}^{I} \mathbb{E}\left[G_i^T \mathbb{E}\left[\beta_i \gamma_i^T\right] G_i\right] \\ &= \sum_{i=1}^{I} \frac{\rho_i}{m_i} \mathbb{E}\left[G_i^T G_i\right] \\ &= \sum_{i=1}^{I} \rho_i.\end{aligned}$$

Local genetic correlation is defined by:

$$r_{ig} = \frac{\rho_i}{\sqrt{h_{1i}^2 h_{2i}^2}}.$$

1.3 Covariance of z scores

In genome-wide association studies (GWAS), summary statistics are more accessible than individual-level genotype data due to potential privacy and data sharing security concerns. For a GWAS with quantitative trait, the z score of a single SNP j is given by:

$$z_j = \frac{\hat{\beta}_j}{se(\hat{\beta}_j)}$$

where $\hat{\beta}_j$ is the estimated coefficient from marginal linear regression between the trait and the SNP j, and $se(\hat{\beta}_j)$ is the corresponding standard error. In practice, we approximate z scores by $z_{1j} = X_{\cdot j}^T \phi_1/\sqrt{n_1}$ and $z_{2j} = Y_{\cdot j}^T \phi_2/\sqrt{n_2}$.

1.3.1 Global

We derive the variance-covariance matrix of $\left(z_1^T, z_2^T\right)^T$ in this section. It's sufficient to show that

$$Cov\left(z_1, z_2\right) = \frac{\sqrt{n_1 n_2}\rho}{m} V^2 + \frac{n_s \rho_t}{\sqrt{n_1 n_2}} V, \tag{1}$$

where $\rho_t = \rho + \rho_e$. Because $Var(z_1)$ and $Var(z_2)$ can be derived from (1). In fact, to compute $Var(z_1)$, we can assume trait 1 and trait 2 are from the same study and hence we have

$$Var(z_1) = Cov(z_1, z_1) = \frac{\sqrt{n_1 n_1} h_1^2}{m} V^2 + \frac{n_1\left[h_1^2 + \left(1 - h_1^2\right)\right]}{\sqrt{n_1 n_1}} V = \frac{n_1 h_1^2}{m} V^2 + V.$$

Similarly, $Cov(z_2) = \left(n_2 h_2^2/m\right) V^2 + V$. To prove (1), we begin with the following proposition.

Proposition 1 *Assume* $\Gamma_1, \Gamma_2 \overset{i.i.d.}{\sim} B(1, p_\Gamma)$; $\Pi_1, \Pi_2 \overset{i.i.d.}{\sim} B(1, p_\Pi)$; $Cor(\Gamma_1, \Pi_1) = Cor(\Gamma_2, \Pi_2) = r_{\Gamma\Pi}$; Γ_1 *and* Π_2 *are independent.* Γ_2 *and* Π_1 *are independent. We have:*

(1).

$$\begin{aligned}&\mathbb{E}\left[\left(\Gamma_1+\Gamma_2-2p_\Gamma\right)^2\left(\Pi_1+\Pi_2-2p_\Pi\right)^2\right]\\=&4\left(r_{\Gamma\Pi}^2+1\right)p_\Pi\left(1-p_\Pi\right)p_\Gamma\left(1-p_\Gamma\right)+2r_{\Gamma\Pi}\sqrt{p_\Pi\left(1-p_\Pi\right)}\sqrt{p_\Gamma\left(1-p_\Gamma\right)}\left(1-2p_\Pi\right)\left(1-2p_\Gamma\right).\end{aligned}$$

(2). Additionally, if $\Lambda_1,\Lambda_2\overset{i.i.d.}{\sim}B(1,p_\Lambda)$*;* $Cor(\Gamma_1,\Lambda_1)=Cor(\Gamma_2,\Lambda_2)=r_{\Gamma\Lambda}$*;* $Cor(\Pi_1,\Lambda_1)=Cor(\Pi_2,\Lambda_2)=r_{\Pi\Lambda}$*;* $\mathbb{E}\left[\left(\Pi_1-p_\Pi\right)\left(\Gamma_1-p_\Gamma\right)\left(\Lambda_1-p_\Lambda\right)\right]=\mathbb{E}\left[\left(\Pi_2-p_\Pi\right)\left(\Gamma_2-p_\Gamma\right)\left(\Lambda_2-p_\Lambda\right)\right]=\Delta$*;* Λ_1 *is independent with* Γ_2 *and* Π_2*;* Λ_2 *is independent with* Γ_1 *and* Π_1*. We have:*

$$\begin{aligned}&\mathbb{E}\left[\left(\Gamma_1+\Gamma_2-2p_\Gamma\right)\left(\Lambda_1+\Lambda_2-2p_\Lambda\right)\left(\Pi_1+\Pi_2-2p_\Pi\right)^2\right]\\=&4\left(r_{\Gamma\Pi}r_{\Pi\Lambda}+r_{\Gamma\Lambda}\right)p_\Pi\left(1-p_\Pi\right)\sqrt{p_\Gamma\left(1-p_\Gamma\right)}\sqrt{p_\Lambda\left(1-p_\Lambda\right)}+2\left(1-2p_\Pi\right)\Delta.\end{aligned}$$

We note the genotype of SNP j before standardization is the sum of two independent idential binomial distribution with p equal to minor allele frequency, $0.05<f_j<0.5$, of SNP j. We only consider common variants. With Hardy-Weinberg equilibrium, for any $1\le\tau\le n_1$, we approximate $X_{\tau j}$ by

$$X_{\tau j}:=\frac{S_{1\tau j}+S_{2\tau j}-2f_j}{\sqrt{2f_j\left(1-f_j\right)}},$$

where $S_{1\tau j}$ and $S_{2\tau j}$ represent allelic dosage for SNP j on each chromosomethe and f_j is the minor allele frequency of SNP j. The approximate of genotype in Y is defined in the same way. By proposition 1, for different SNP, SNP j, SNP ζ, we have

$$\mathbb{E}\left[X_{\tau j}^2X_{\tau\zeta}^2\right]=V_{j\zeta}^2+1+\frac{V_{j\zeta}\left(1-2f_j\right)\left(1-2f_\zeta\right)}{2\sqrt{f_j\left(1-f_j\right)}\sqrt{f_\zeta\left(1-f_\zeta\right)}}.$$

For SNP k, $k\neq j,\zeta$, denoting $\Delta_{jk\zeta}=\mathbb{E}\left[\left(S_{\alpha\tau j}-f_j\right)\left(S_{\alpha\tau k}-f_k\right)\left(S_{\alpha\tau\zeta}-f_\zeta\right)\right]$, $\alpha=1,2$, we have

$$\mathbb{E}\left[X_{\tau j}X_{\tau k}X_{\tau\zeta}^2\right]=V_{j\zeta}V_{k\zeta}+V_{jk}+\frac{\left(1-2f_\zeta\right)\Delta_{jk\zeta}}{2f_\zeta\left(1-f_\zeta\right)\sqrt{f_j\left(1-f_j\right)}\sqrt{f_k\left(1-f_k\right)}},$$

where $V_{j\zeta}$ represents the j-th row, ζ-th column of LD matrix V. In other words, $V_{j\zeta}$ is the correlation of genotypes between SNP j and SNP ζ. V_{jk} and $V_{k\zeta}$ are defined in the same way.

Now, we prove (1). We have

$$\begin{aligned}\mathbb{E}\left[z_1z_2^T\right]&=\mathbb{E}\left[\frac{X^T\phi_1}{\sqrt{n_1}}\cdot\frac{\phi_2^TY}{\sqrt{n_2}}\right]=\mathbb{E}\left[\frac{X^T\left(X\beta+\epsilon\right)}{\sqrt{n_1}}\cdot\frac{\left(Y\gamma+\delta\right)^TY}{\sqrt{n_2}}\right]\\&=\mathbb{E}\left[\frac{X^TX\beta\gamma^TY^TY}{\sqrt{n_1n_2}}\right]+\mathbb{E}\left[\frac{X^T\epsilon\delta^TY}{\sqrt{n_1n_2}}\right]\\&=\mathbb{E}\left\{\mathbb{E}\left[\frac{X^TX\beta\gamma^TY^TY}{\sqrt{n_1n_2}}\middle|X,Y\right]\right\}+\mathbb{E}\left\{\mathbb{E}\left[\frac{X^T\epsilon\delta^TY}{\sqrt{n_1n_2}}\middle|X,Y\right]\right\}\\&=\mathbb{E}\left\{\frac{X^TX\mathbb{E}\left[\beta\gamma^T\right]Y^TY}{\sqrt{n_1n_2}}\right\}+\mathbb{E}\left\{\frac{X^T\mathbb{E}\left[\epsilon\delta^T\right]Y}{\sqrt{n_1n_2}}\right\}\\&=\frac{\rho}{m\sqrt{n_1n_2}}\mathbb{E}\left[X^TXY^TY\right]+\frac{\rho_e}{\sqrt{n_1n_2}}\mathbb{E}\left[\sum_{i=1}^{n_s}X_{i\cdot}^TY_{i\cdot}\right]\\&=\frac{\rho}{m\sqrt{n_1n_2}}\mathbb{E}\left[X^TXY^TY\right]+\frac{\rho_en_s}{\sqrt{n_1n_2}}V.\end{aligned}\tag{2}$$

To complete the calculation, we next compute $\mathbb{E}\left[X_{\cdot j}^T X Y^T Y_{\cdot k}\right]$. First, we denote $\mathcal{N} = \left\{(\tau,\eta) \,\middle|\, \tau = \eta = 1, 2, \ldots, n_s\right\}$. For $j = k$, we have

$$\begin{aligned}
\mathbb{E}\left[X_{\cdot j}^T X Y^T Y_{\cdot j}\right] &= \mathbb{E}\left[\sum_{\zeta=1}^{m}\left(\sum_{\tau=1}^{n_1} X_{\tau j} X_{\tau\zeta}\right)\cdot\left(\sum_{\eta=1}^{n_2} Y_{\eta j} Y_{\eta\zeta}\right)\right] \\
&= \sum_{\zeta=1}^{m}\sum_{\tau=1}^{n_1}\sum_{\eta=1}^{n_2}\mathbb{E}\left[X_{\tau j}X_{\tau\zeta}Y_{\eta j}Y_{\eta\zeta}\right] \\
&= \sum_{\zeta=1}^{m}\left(\sum_{(\tau,\eta)\in\mathcal{N}}\mathbb{E}\left[X_{\tau j}X_{\tau\zeta}Y_{\eta j}Y_{\eta\zeta}\right] + \sum_{(\tau,\eta)\notin\mathcal{N}}\mathbb{E}\left[X_{\tau j}X_{\tau\zeta}\right]\mathbb{E}\left[Y_{\eta j}Y_{\eta\zeta}\right]\right) \\
&= \sum_{\zeta=1}^{m}\left(\sum_{(\tau,\eta)\in\mathcal{N}}\mathbb{E}\left[X_{\tau j}^2 X_{\tau\zeta}^2\right] + (n_1 n_2 - n_s)\,V_{j\zeta}^2\right) \\
&= \sum_{\zeta=1}^{m}\left(n_s\left[V_{j\zeta}^2 + 1 + \frac{V_{j\zeta}\,(1-2f_j)\,(1-2f_\zeta)}{2\sqrt{f_j\,(1-f_j)}\sqrt{f_\zeta\,(1-f_\zeta)}}\right] + (n_1 n_2 - n_s)\,V_{j\zeta}^2\right) \\
&= n_1 n_2 \sum_{\zeta=1}^{m} V_{j\zeta}^2 + \sum_{\zeta=1}^{m} n_s + \sum_{\zeta=1}^{m}\frac{n_s V_{j\zeta}\,(1-2f_j)\,(1-2f_\zeta)}{2\sqrt{f_j\,(1-f_j)}\sqrt{f_\zeta\,(1-f_\zeta)}} \\
&= n_1 n_2 \sum_{\zeta=1}^{m} V_{j\zeta}^2 + m n_s + n_s\sum_{\zeta=1}^{m}\frac{V_{j\zeta}\,(1-2f_j)\,(1-2f_\zeta)}{2\sqrt{f_j\,(1-f_j)}\sqrt{f_\zeta\,(1-f_\zeta)}} \\
&= n_1 n_2 \sum_{\zeta=1}^{m} V_{j\zeta}^2 + m n_s + o(n_s m) \\
&\approx n_1 n_2 \sum_{\zeta=1}^{m} V_{j\zeta}^2 + m n_s. \qquad (3)
\end{aligned}$$

We explain the approximation in (3) here. In the deivation below, C represents a generic constant, whose value may be different at different places. As mentioned above, we only consider common variants in our supplementary note, which means $0.05 < f_j < 0.5$ for any SNP j. So, for any SNP j and ζ, there exists a constant C so that

$$\frac{(1-2f_j)\,(1-2f_\zeta)}{2\sqrt{f_j\,(1-f_j)}\sqrt{f_\zeta\,(1-f_\zeta)}} < C.$$

By Cauchy-Schwarz inequality, we have

$$\sum_{\zeta=1}^{m}\frac{V_{j\zeta}\,(1-2f_j)\,(1-2f_\zeta)}{2\sqrt{f_j\,(1-f_j)}\sqrt{f_\zeta\,(1-f_\zeta)}} < C\sum_{\zeta=1}^{m} V_{j\zeta} \le C\sqrt{m\sum_{\zeta=1}^{m} V_{j\zeta}^2} = C\sqrt{m l_j}. \qquad (4)$$

So, we have

$$n_s\sum_{\zeta=1}^{m}\frac{V_{j\zeta}\,(1-2f_j)\,(1-2f_\zeta)}{2\sqrt{f_j\,(1-f_j)}\sqrt{f_\zeta\,(1-f_\zeta)}} \le C\cdot l_j\cdot n_s\sqrt{m} = o(n_s m)$$

For $j \neq k$, we have

$$\begin{aligned}
\mathbb{E}\left[X_{\cdot j}^T X Y^T Y_{\cdot k}\right] &= \mathbb{E}\left[\sum_{\zeta=1}^{m}\left(\sum_{\tau=1}^{n_1} X_{\tau j}X_{\tau\zeta}\right)\cdot\left(\sum_{\eta=1}^{n_2} Y_{\eta k}Y_{\eta\zeta}\right)\right] \\
&= \sum_{\zeta=1}^{m}\sum_{\tau=1}^{n_1}\sum_{\eta=1}^{n_2}\mathbb{E}\left[X_{\tau j}X_{\tau\zeta}Y_{\eta k}Y_{\eta\zeta}\right] \\
&= \sum_{\zeta=1}^{m}\left(\sum_{(\tau,\eta)\in\mathcal{N}}\mathbb{E}\left[X_{\tau j}X_{\tau\zeta}Y_{\eta k}Y_{\eta\zeta}\right] + \sum_{(\tau,\eta)\notin\mathcal{N}}\mathbb{E}\left[X_{\tau j}X_{\tau\zeta}\right]\mathbb{E}\left[Y_{\eta k}Y_{\eta\zeta}\right]\right) \\
&= \sum_{\zeta=1}^{m}\left(\sum_{(\tau,\eta)\in\mathcal{N}}\mathbb{E}\left[X_{\tau j}X_{\tau k}X_{\tau\zeta}^2\right] + \left(n_1n_2 - n_s\right)V_{j\zeta}V_{k\zeta}\right) \\
&= \sum_{\zeta=1}^{m}\left(n_s\left[V_{j\zeta}V_{k\zeta} + V_{jk} + \frac{\left(1-2f_\zeta\right)\Delta_{jk\zeta}}{2f_\zeta\left(1-f_\zeta\right)\sqrt{f_j\left(1-f_j\right)}\sqrt{f_k\left(1-f_k\right)}}\right] + \left(n_1n_2-n_s\right)V_{j\zeta}V_{k\zeta}\right) \\
&= n_1n_2\sum_{\zeta=1}^{m}V_{j\zeta}V_{k\zeta} + \sum_{\zeta=1}^{m}n_sV_{jk} + \sum_{\zeta=1}^{m}\frac{n_s\left(1-2f_\zeta\right)\Delta_{jk\zeta}}{2f_\zeta\left(1-f_\zeta\right)\sqrt{f_j\left(1-f_j\right)}\sqrt{f_k\left(1-f_k\right)}} \\
&= n_1n_2\sum_{\zeta=1}^{m}V_{j\zeta}V_{k\zeta} + mn_sV_{jk} + n_s\sum_{\zeta=1}^{m}\frac{\left(1-2f_\zeta\right)\Delta_{jk\zeta}}{2f_\zeta\left(1-f_\zeta\right)\sqrt{f_j\left(1-f_j\right)}\sqrt{f_k\left(1-f_k\right)}} \\
&= n_1n_2\sum_{\zeta=1}^{m}V_{j\zeta}V_{k\zeta} + mn_sV_{jk} + o(n_sm) \\
&\approx n_1n_2\sum_{\zeta=1}^{m}V_{j\zeta}V_{k\zeta} + mn_sV_{jk}. \qquad (5)
\end{aligned}$$

We note that $\Delta_{jk\zeta} \leq V_{j\zeta}$ by definition. So, the approximation in (5) is derived from the same argument as (4).

Combine (3) and (5) we have

$$\mathbb{E}\left[X^TXY^TY\right] \approx n_1n_2V^2 + mn_sV \qquad (6)$$

Substituting (6) into (2), we finish the calculation for $Cov\left(z_1, z_2\right)$.

1.3.2 Local

We assume SNPs from different local regions are indepedent. In other words, we assume LD matrix takes the block structure

$$V = \begin{pmatrix} V_1 & & & \\ & V_2 & & \\ & & \ddots & \\ & & & V_I \end{pmatrix},$$

where I is the number of locla regions.

Based on the derivations for global genetic covariance above, we generalize the results to

calculate covariance of local z scores, $Cov(z_{1i}, z_{2i})$. We have

$$
\begin{aligned}
\mathbb{E}\left[z_{1i}z_{2i}^T\right] &= \mathbb{E}\left[\frac{X_i^T\phi_1}{\sqrt{n_1}}\cdot\frac{\phi_2^TY_i}{\sqrt{n_2}}\right] = \mathbb{E}\left[\frac{X_i^T\left(\sum_{\nu=1}^I X_\nu\beta_\nu+\epsilon\right)}{\sqrt{n_1}}\cdot\frac{\left(\sum_{\nu=1}^I Y_\nu\gamma_\nu+\delta\right)^TY_i}{\sqrt{n_2}}\right] \\
&= \sum_{\nu=1}^I \mathbb{E}\left[\frac{X_i^TX_\nu\beta_\nu\gamma_\nu^TY_\nu^TY_i}{\sqrt{n_1n_2}}\right] + \mathbb{E}\left[\frac{X_i^T\epsilon\delta^TY_i}{\sqrt{n_1n_2}}\right] \\
&= \mathbb{E}\left[\frac{X_i^TX_i\beta_i\gamma_i^TY_i^TY_i}{\sqrt{n_1n_2}}\right] + \sum_{\nu\neq i}\mathbb{E}\left[\frac{X_i^TX_\nu\beta_\nu\gamma_\nu^TY_\nu^TY_i}{\sqrt{n_1n_2}}\right] + \frac{n_s\rho_e}{\sqrt{n_1n_2}}V_i \\
&= \frac{\rho_i}{m_i}\mathbb{E}\left[\frac{X_i^TX_iY_i^TY_i}{\sqrt{n_1n_2}}\right] + \sum_{\nu\neq i}\frac{\rho_\nu}{m_\nu}\mathbb{E}\left[\frac{X_i^T\mathbb{E}\left(X_\nu Y_\nu^T\right)Y_i}{\sqrt{n_1n_2}}\right] + \frac{n_s\rho_e}{\sqrt{n_1n_2}}V_i \\
&\approx \frac{\sqrt{n_1n_2}\rho_i}{m_i}V_i^2 + \frac{n_s\rho_i}{\sqrt{n_1n_2}}V_i + \sum_{\nu\neq i}\frac{n_s\rho_\nu}{\sqrt{n_1n_2}}V_i + \frac{n_s\rho_e}{\sqrt{n_1n_2}}V_i \qquad (7) \\
&= \frac{\sqrt{n_1n_2}\rho_i}{m_i}V_i^2 + \frac{n_s\rho_t}{\sqrt{n_1n_2}}V_i.
\end{aligned}
$$

Here, $\rho_t = \sum_{i=1}^I \rho_i + \rho_e$. Approximation in (7) follows from (6).

1.4 Variance of $\tilde{\mathbf{z}}_{\mathbf{1ij}}\tilde{\mathbf{z}}_{\mathbf{2ij}}$

Assume eigen decomposition of V_i is $V_i = U_i\Sigma_iU_i^T$. Denote $\tilde{z}_{1i} = U_i^Tz_{1i}$ and $\tilde{z}_{2i} = U_i^Tz_{2i}$. Then, we have

$$
\begin{pmatrix}\tilde{z}_{1i}\\ \tilde{z}_{2i}\end{pmatrix} \sim N\left(0, \begin{pmatrix}\frac{n_1h_{1i}^2}{m_i}\Sigma_i^2+\Sigma_i & \frac{\sqrt{n_1n_2}\rho_i}{m_i}\Sigma_i^2+\frac{n_s\rho_t}{\sqrt{n_1n_2}}\Sigma_i \\ \frac{\sqrt{n_1n_2}\rho_i}{m_i}\Sigma_i^2+\frac{n_s\rho_t}{\sqrt{n_1n_2}}\Sigma_i & \frac{n_2h_{2i}^2}{m_i}\Sigma_i^2+\Sigma_i\end{pmatrix}\right). \qquad (8)
$$

We order the eigenvalues in Σ_i by their values. We denote the eigenvalues by $w_{i1} \leq w_{i2} \leq \ldots \leq w_{im_i}$. $\tilde{z}_{1ij}$ and $\tilde{z}_{2ij}$ denote the j-th element of $\tilde{z}_{1i}$ and $\tilde{z}_{2i}$, respectively. We use the following proposition to obtain $Var\left(\tilde{z}_{1ij}\tilde{z}_{2ij}\right)$.

Proposition 2 *Assume*

$$
\begin{pmatrix}\xi_1\\ \xi_2\end{pmatrix} \sim N\left(0, \begin{pmatrix}\sigma_1^2 & \rho_0\\ \rho_0 & \sigma_2^2\end{pmatrix}\right)
$$

we have $Var\left(\xi_1\xi_2\right) = \rho_0^2 + \sigma_1^2\sigma_2^2$.

By proposition 2, we have

$$
var\left[\tilde{z}_{1ij}\tilde{z}_{2ij}\right] = \left(\frac{\sqrt{n_1n_2}\rho_i}{m_i}w_{ij}^2 + \frac{n_s\rho_t}{\sqrt{n_1n_2}}w_{ij}\right)^2 + \left(\frac{n_1h_{1i}^2}{m_i}w_{ij}^2 + w_{ij}\right)\left(\frac{n_2h_{2i}^2}{m_i}w_{ij}^2 + w_{ij}\right). \qquad (9)
$$

1.5 Variance of $\hat{\rho}_\mathbf{i}$

From (8), we have

$$
\mathbb{E}\left[\tilde{z}_{1ij}\tilde{z}_{2ij}\right] = \frac{\sqrt{n_1n_2}\rho_i}{m_i}w_{ij}^2 + \frac{n_s\rho_t}{\sqrt{n_1n_2}}w_{ij}.
$$

We obtain the estimates of ρ_i by weighted linear regression that regresses $\tilde{z}_{1ij}\tilde{z}_{2ij} - \frac{n_s\rho_t}{\sqrt{n_1n_2}}w_{ij}$ on w_{ij}^2. We use LD score regression[2] to obtain the estimates of $\frac{n_s\rho_t}{\sqrt{n_1n_2}}$, which is denoted by $\widehat{n_s\rho_t}/\sqrt{n_1n_2}$. The weights are given by the reciprocal of the variance in (9). In practice, we use $\left(\frac{n_1h_{1i}^2}{m_i}w_{ij}^2 + w_{ij}\right)\left(\frac{n_2h_{2i}^2}{m_i}w_{ij}^2 + w_{ij}\right)$ to approximate the variance, $\mathbb{E}\left[\tilde{z}_{1ij}\tilde{z}_{2ij}\right]$. For notation

simplicity, we denote $q_{ij}^2 = \left(\frac{n_1 h_{1i}^2}{m_i} w_{ij}^2 + w_{ij}\right)\left(\frac{n_2 h_{2i}^2}{m_i} w_{ij}^2 + w_{ij}\right)$ and $\eta_{ij} = \tilde{z}_{1ij}\tilde{z}_{2ij} - \frac{n_s \rho_t}{\sqrt{n_1 n_2}} w_{ij}$. We note $Var\left(n_{ij}\right) = q_{ij}^2$. We use the K_i largest eigenvalues in the estimation of local genetic covariance to reduce noise.

1.5.1 Theoretical and empirical variance of $\hat{\rho}_\mathbf{i}$

From weighted linear regression, the weighted estimate of local genetic covariance is given by

$$\hat{\rho}_i = \frac{m_i}{\sqrt{n_1 n_2}} \frac{\sum_{j=1}^{K_i} \eta_{ij} w_{ij}^2 / q_{ij}^2}{\sum_{j=1}^{K_i} w_{ij}^4 / q_{ij}^2}. \tag{10}$$

So, the theoretical variance of $\hat{\rho}_i$ is given by

$$Var\left(\hat{\rho}_i\right) = \left(\frac{m_i^2}{n_1 n_2}\right) Var\left(\frac{\sum_{j=1}^{K_i} \eta_{ij} w_{ij}^2 / q_{ij}^2}{\sum_{j=1}^{K_i} w_{ij}^4 / q_{ij}^2}\right) = \left(\frac{m_i^2}{n_1 n_2}\right) \frac{\sum_{j=1}^{K_i} Var\left(\eta_{ij}\right) w_{ij}^4 / q_{ij}^4}{\left(\sum_{j=1}^{K_i} w_{ij}^4 / q_{ij}^2\right)^2} = \frac{m_i^2 / \left(n_1 n_2\right)}{\sum_{j=1}^{K_i} w_{ij}^4 / q_{ij}^2}.$$

We assume the variance of η_{ij} is proportional to q_{ij} and given by $q_{ij}\sigma_{\eta i}^2$ although we know the true value of $\sigma_{\eta i}^2$ is equal to 1. In other words, we treat the variance of η_{ij} as unknown and use weighted linear regression procedure to estimate $\sigma_{\eta i}^2$. The estimate of $\sigma_{\eta i}^2$ is denoted by $\hat{\sigma}_{\eta i}^2$. Then, multiply the theoretical variance by $\hat{\sigma}_{\eta i}^2$, we have the empirical variance of $\hat{\rho}_i$

$$\widehat{Var}\left(\hat{\rho}_i\right) = Var\left(\hat{\rho}_i\right) \hat{\sigma}_{\eta i}^2. \tag{11}$$

We obtain $\hat{\sigma}_{\eta i}^2$ by the residues from the regression model. We have

$$\hat{\sigma}_{\eta i}^2 = \left[\sum_{j=1}^{K_i} \frac{\eta_{ij}^2}{q_{ij}^2} - \frac{\left(\sum_{j=1}^{K_i} \eta_{ij} w_{ij}^2 / q_{ij}^2\right)^2}{\sum_{j=1}^{K_i} w_{ij}^4 / q_{ij}^2}\right] \Bigg/ \left(K_i - 1\right). \tag{12}$$

Substituting (12) into (11), we have

$$\widehat{Var}\left(\hat{\rho}_i\right) = \frac{m_i^2 / \left(n_1 n_2\right)}{\sum_{j=1}^{K_i} w_{ij}^4 / q_{ij}^2} \cdot \left[\sum_{j=1}^{K_i} \frac{\eta_{ij}^2}{q_{ij}^2} - \frac{\left(\sum_{j=1}^{K_i} \eta_{ij} w_{ij}^2 / q_{ij}^2\right)^2}{\sum_{j=1}^{K_i} w_{ij}^4 / q_{ij}^2}\right] \Bigg/ \left(K_i - 1\right). \tag{13}$$

1.5.2 Approximation of $\mathbf{Var}\left(\mathbb{E}\left[\hat{\rho}_\mathbf{i} \middle| \left(\widehat{\mathbf{n_s}\rho_\mathbf{t}} / \sqrt{\mathbf{n_1 n_2}}\right)\right]\right)$

To compensate the noise introduced by the estimation of $\widehat{n_s\rho_t}/\sqrt{n_1 n_2}$ in LD score regression[2], we add $Var\left(\mathbb{E}\left[\hat{\rho}_i \middle| \left(\widehat{n_s\rho_t}/\sqrt{n_1 n_2}\right)\right]\right)$ to the total variance, $Var\left(\hat{\rho}_i\right)$. Then, the total variance of $\hat{\rho}_i$ is then given by

$$Var\left(\hat{\rho}_i\right) = Var\left(\mathbb{E}\left[\hat{\rho}_i \middle| \frac{\widehat{n_s\rho_t}}{\sqrt{n_1 n_2}}\right]\right) + \mathbb{E}\left(Var\left[\hat{\rho}_i \middle| \frac{\widehat{n_s\rho_t}}{\sqrt{n_1 n_2}}\right]\right).$$

The estimation of $\mathbb{E}\left(Var\left[\hat{\rho}_i|\left(\widehat{n_s\rho_t}/\sqrt{n_1n_2}\right)\right]\right)$ is given by the empirical variance in (13). In this section, we derive the expression of $Var\left(\mathbb{E}\left[\hat{\rho}_i|\left(\widehat{n_s\rho_t}/\sqrt{n_1n_2}\right)\right]\right)$. By (10), We have

$$\begin{aligned}
\mathbb{E}\left[\hat{\rho}_i\middle|\frac{\widehat{n_s\rho_t}}{\sqrt{n_1n_2}}\right] &= \frac{m_i}{\sqrt{n_1n_2}}\mathbb{E}\left[\frac{\sum_{j=1}^{K_i}\eta_{ij}w_{ij}^2/q_{ij}^2}{\sum_{j=1}^{K_i}w_{ij}^4/q_{ij}^2}\middle|\frac{\widehat{n_s\rho_t}}{\sqrt{n_1n_2}}\right] \\
&= \frac{m_i}{\sqrt{n_1n_2}}\frac{\sum_{j=1}^{K_i}\mathbb{E}\left[\eta_{ij}|\left(\widehat{n_s\rho_t}/\sqrt{n_1n_2}\right)\right]w_{ij}^2/q_{ij}^2}{\sum_{j=1}^{K_i}w_{ij}^4/q_{ij}^2} \\
&= \frac{m_i}{\sqrt{n_1n_2}}\frac{\sum_{j=1}^{K_i}\left(\mathbb{E}\left[\tilde{z}_{1ij}\tilde{z}_{2ij}\right]-\frac{\widehat{n_s\rho_t}}{\sqrt{n_1n_2}}w_{ij}\right)w_{ij}^2/q_{ij}^2}{\sum_{j=1}^{K_i}w_{ij}^4/q_{ij}^2} \\
&= \frac{m_i}{\sqrt{n_1n_2}}\frac{\sum_{j=1}^{K_i}\mathbb{E}\left[\tilde{z}_{1ij}\tilde{z}_{2ij}\right]w_{ij}^2/q_{ij}^2}{\sum_{j=1}^{K_i}w_{ij}^4/q_{ij}^2} - \frac{m_i}{\sqrt{n_1n_2}}\frac{\widehat{n_s\rho_t}}{\sqrt{n_1n_2}}\frac{\sum_{j=1}^{K_i}w_{ij}^3/q_{ij}^2}{\sum_{j=1}^{K_i}w_{ij}^4/q_{ij}^2}. \qquad (14)
\end{aligned}$$

The first term in (14) is constant. So, $Var\left(\mathbb{E}\left[\hat{\rho}_i|\left(\widehat{n_s\rho_t}/\sqrt{n_1n_2}\right)\right]\right)$ is given by

$$\begin{aligned}
Var\left(\mathbb{E}\left[\hat{\rho}_i\middle|\frac{\widehat{n_s\rho_t}}{\sqrt{n_1n_2}}\right]\right) &= Var\left(\frac{m_i}{\sqrt{n_1n_2}}\frac{\widehat{n_s\rho_t}}{\sqrt{n_1n_2}}\frac{\sum_{j=1}^{K_i}w_{ij}^3/q_{ij}^2}{\sum_{j=1}^{K_i}w_{ij}^4/q_{ij}^2}\right) \\
&= \frac{m_i^2}{n_1n_2}\left(\frac{\sum_{j=1}^{K_i}w_{ij}^3/q_{ij}^2}{\sum_{j=1}^{K_i}w_{ij}^4/q_{ij}^2}\right)^2 Var\left(\frac{\widehat{n_s\rho_t}}{\sqrt{n_1n_2}}\right).
\end{aligned}$$

$Var\left(\widehat{n_s\rho_t}/\sqrt{n_1n_2}\right)$ is the variance of the intercept estimated by LD score regression.

2 Consistency of SUPERGNOVA and GNOVA

The estimates of SUPERGNOVA is approximately consistent with the estimates of GNOVA[4] when the number of SNPs m is large enough. The estimates of GNOVA corrected for sample overlap is given by

$$\hat{\rho}_{gnova} = \frac{m\left(\sqrt{n_1n_2}z_1^Tz_2 - mn_s\rho_t\right)}{n_1n_2\sum_{j=1}^m l_j}. \qquad (15)$$

Without loss of generality, we demonstrate the equivalence between SUPERGNOVA and GNOVA for global genetic covariance estimation and we use all the eigenvalues of LD matrix. By (10), the SUPERGNOVA estimator for global genetic covariance is given by

$$\hat{\rho} = \frac{m}{\sqrt{n_1n_2}}\frac{\sum_{j=1}^m\eta_jw_j^2/q_j^2}{\sum_{j=1}^m w_j^4/q_j^2},$$

with q_j, w_j and η_j are defined in the obvious way. We assume per SNP heritability is small so that we can approximate q_j^2 by w_j^2. So, the SUPERGNOVA estimator can be written as:

$$\begin{aligned}\hat{\rho} &= \frac{m}{\sqrt{n_1 n_2}} \frac{\sum_{j=1}^{m}\left(\tilde{z}_{1j}\tilde{z}_{2j} - \frac{n_s \rho_t}{\sqrt{n_1 n_2}} w_j\right)}{\sum_{j=1}^{m} w_j^2} \\ &= \frac{m}{\sqrt{n_1 n_2}} \frac{\tilde{z}_1^T \tilde{z}_2 - \sum_{j=1}^{m} \frac{n_s \rho_t}{\sqrt{n_1 n_2}} w_j}{\sum_{j=1}^{m} w_j^2} \\ &= \frac{m}{\sqrt{n_1 n_2}} \frac{z_1^T U U^T z_2 - \frac{n_s \rho_t}{\sqrt{n_1 n_2}} tr\left(V\right)}{tr\left(V^2\right)} \\ &= \frac{m}{\sqrt{n_1 n_2}} \frac{z_1^T z_2 - \frac{m n_s \rho_t}{\sqrt{n_1 n_2}}}{\sum_{j=1}^{m} l_j} \\ &= \frac{m\left(\sqrt{n_1 n_2} z_1^T z_2 - m n_s \rho_t\right)}{n_1 n_2 \sum_{j=1}^{m} l_j},\end{aligned}$$

which is exactly the GNOVA estimator given in (15).

3 Liability threshold model for SUPERGNOVA

In this section, we demonstrate that the SUPERGNOVA framework can be used in ascertained case/control studies. Let P_k denotes the sample prevalence of ϕ_k in study k for $k = 1, 2$. Directly following LDSC[2], we compute z-scores from liability model

$$z_{kj} = \frac{\sqrt{n_k P_k \left(1 - P_k\right)} \left(\hat{p}_{cas,kj} - \hat{p}_{con,kj}\right)}{\sqrt{\hat{p}_{kj}\left(1 - \hat{p}_{kj}\right)}}, \tag{16}$$

where $\hat{p}_{kj}$ denotes alleles frequency in the entire sample, $\hat{p}_{cas,kj}$ and $\hat{p}_{con,kj}$ denote allele frequency for study k in case samples and control samples, respectively. We assume the MAF of a single SNP doesn't have much difference between the two studies and both equal to population MAF i.e. $\hat{p}_{1j} = \hat{p}_{2j} = f_j$, where f_j is the MAF of SNP j. That is because we assume an infinitesimal model and the contribution of a signle SNP is small. We assume the selection of sample for study k only based on phenotype k.Otherwise, $\hat{p}_{cas,kj}$ will be a biased estimate of $p_{cas,kj}$.

In the liability threshold (probit) model, the binary trait is determined by continuous liability ψ, i.e. $\phi = 1\left[\psi > \tau\right]$, where τ is the liability threshold. The liability threshold τ is determined by $\tau = \Phi^{-1}\left(1 - K\right)$, where Φ is the standard normal cdf and K is the population prevalence. We first state the theory for genome-wide genetic covariance. As an analogy of quantitative trait, we model continuous liability by

$$\begin{aligned}\psi_1 &= X\beta + \epsilon \\ \psi_2 &= Y\gamma + \delta,\end{aligned}$$

where the distributions of X, Y, β, γ, ϵ, and δ and the definition of heritability and genetic covariance are the same as those in quantitative trait model. Since we assume an additive model and Hardy-Weinberg equilibrium, following the arguments in LDSC[2], without loss of generality, we can state the proofs in terms of haploid genotypes. We further denote the 0-1 coded genotype matrices as G and H for notation simplicity. So, we have $X_{ij} = \left(G_{ij} - f_j\right) / \sqrt{f_j\left(1 - f_j\right)}$ and $Y_{ij} = \left(H_{ij} - f_j\right) / \sqrt{f_j\left(1 - f_j\right)}$, where f_j is the MAF of SNP j if X and Y are haploid standardized genotype matrices. Since we assume an infinitesimal model, the effect of a single SNP is

small and the MAF of a SNP doesn't have much difference between the two studies. We define

$$\begin{aligned} p_{cas,1j} &= P\left[G_{ij}=1|\phi_{1i}=1\right] \\ p_{con,1j} &= P\left[G_{ij}=1|\phi_{1i}=0\right] \\ p_{cas,2j} &= P\left[H_{ij}=1|\phi_{2i}=1\right] \\ p_{con,2j} &= P\left[H_{ij}=1|\phi_{2i}=0\right] \end{aligned}$$

as the allele frequencies of SNP j in cases and controls for study 1 and study 2, respectively, where i represents a generic individual in study 1 or study 2.

Proposition 3 *Under the liability model above, we have*

$$\begin{aligned} \mathbb{E}\left[\hat{p}_{cas,1j}\right]-\mathbb{E}\left[\hat{p}_{con,1j}\right] &= 0 \\ \mathbb{E}\left[\hat{p}_{cas,2j}\right]-\mathbb{E}\left[\hat{p}_{con,2j}\right] &= 0 \end{aligned}$$

proof. The distribution of ψ_{1i} given G_{ij} and β is $\psi_{1i}|\left(G_{ij},\beta\right)\sim N\left(V_j^T\beta,1\right)$. The variance is 1 because we assume an infinitesimal model and the marginal heritability explained by a single SNP is small. So, we have

$$\begin{aligned} P\left(\phi_{1i}=1|X_{ij},\beta\right) &= P\left(\psi_{1i}>\tau_1|X_{ij},\beta\right) \\ &= 1-\Phi\left(\tau_1-X_{ij}V_j^T\beta\right) \\ &\approx K_1+\phi\left(\tau_1\right)X_{ij}V_j^T\beta, \end{aligned} \tag{17}$$

where ϕ is the standard normal density. Taylor expansion at τ_1 is used in the above approximation. By the results in (17), we have

$$\begin{aligned} \mathbb{E}\left[\hat{p}_{cas,1j}\right] &= \mathbb{E}\left[\frac{\sum_{\phi_{1i}=1}1\left\{G_{ij}=1\right\}}{n_1P_1}\right] \\ &= \frac{n_1P_1\mathbb{E}\left[1\left\{G_{ij}=1\right\}|\phi_{1i}=1\right]}{n_1P_1} \\ &= P\left[G_{ij}=1|\phi_{1i}=1\right] \\ &= \frac{P\left[\phi_{1i}=1|G_{ij}=1\right]P\left[G_{ij}=1\right]}{P\left[\phi_{1i}=1\right]} \\ &= \frac{f_j}{K_1}\cdot P\left[\phi_{1i}=1|G_{ij}=1\right] \\ &= \frac{f_j}{K_1}\cdot \mathbb{E}\left[P\left(\phi_{1i}=1|G_{ij}=1,\beta\right)\right] \\ &= \frac{f_j}{K_1}\cdot \mathbb{E}\left[K_1+\phi\left(\tau_1\right)\sqrt{\frac{1-f_j}{f_j}}V_j^T\beta\right] \\ &= f_j. \end{aligned}$$

We compute $\mathbb{E}\left[\hat{p}_{cas,2j}\right]$, $\mathbb{E}\left[\hat{p}_{cas,2j}\right]$, and $\mathbb{E}\left[\hat{p}_{con,2j}\right]$ with the same argument and complete the proof.

By proposition 3, we have

$$\begin{aligned} \mathbb{E}\left[z_{kj}\right] &= \mathbb{E}\left[\frac{\sqrt{n_kP_k\left(1-P_k\right)}\left(\hat{p}_{cas,kj}-\hat{p}_{con,kj}\right)}{\sqrt{\hat{p}_{kj}\left(1-\hat{p}_{kj}\right)}}\right] \\ &\approx \frac{\sqrt{n_kP_k\left(1-P_k\right)}\mathbb{E}\left[\left(\hat{p}_{cas,kj}-\hat{p}_{con,kj}\right)\right]}{\mathbb{E}\left[\sqrt{\hat{p}_{kj}\left(1-\hat{p}_{kj}\right)}\right]} \\ &= 0. \end{aligned} \tag{18}$$

The approximation in (18) ignore $\mathcal{O}(1/n)$ term and put the expectation onto the nominator and denominator.

By Lemma 2 in the supplementary note of LDSC[2], we have

$$\begin{aligned}
\mathbb{E}\left[p_{cas,1j}-p_{con,1j}\right] &= 0,\\
\mathbb{E}\left[p_{cas,2j}-p_{con,2j}\right] &= 0,\\
Var\left[p_{cas,1j}-p_{con,1j}\right] &= \frac{f_j\left(1-f_j\right)\phi\left(\tau_1\right)^2 h_1^2}{mK_1^2\left(1-K_1\right)^2}l_j,\\
Var\left[p_{cas,2j}-p_{con,2j}\right] &= \frac{f_j\left(1-f_j\right)\phi\left(\tau_2\right)^2 h_2^2}{mK_2^2\left(1-K_2\right)^2}l_j,\\
Cov\left[p_{cas,1j}-p_{con,1j},p_{cas,2j}-p_{con,2j}\right] &= \frac{f_j\left(1-f_j\right)\phi\left(\tau_1\right)\phi\left(\tau_2\right)\rho}{mK_1\left(1-K_1\right)K_2\left(1-K_2\right)}l_j.
\end{aligned}$$

We want to compute $Cov\left[p_{cas,1j}-p_{con,1j},p_{cas,2\zeta}-p_{con,2\zeta}\right]$, for $j\neq\zeta$, which is equivalent to $\mathbb{E}\left[\left(p_{cas,1j}-p_{con,1j}\right)\left(p_{cas,2\zeta}-p_{con,2\zeta}\right)\right]$, to generalize the theory to SUPERGNOVA.

Proposition 4 *Under the liability model above, we have*

$$\mathbb{E}\left[\left(p_{cas,1j}-p_{con,1j}\right)\left(p_{cas,2\zeta}-p_{con,2\zeta}\right)\right] = \frac{\sqrt{f_j\left(1-f_j\right)}\sqrt{f_\zeta\left(1-f_\zeta\right)}\phi\left(\tau_1\right)\phi\left(\tau_2\right)\rho}{mK_1\left(1-K_1\right)K_2\left(1-K_2\right)}V_j^T V_\zeta$$

Proof. Following the results in proposition 3, we have

$$\begin{aligned}
p_{cas,1j}|\beta &= P\left(G_{ij}=1|\phi_{1i}=1,\beta\right)\\
&= \frac{P\left(\phi_{1i}=1|G_{ij}=1,\beta\right)P\left(G_{ij}=1\right)}{P\left(\phi_{1i}=1\right)}\\
&= \frac{f_j}{K_1}\cdot\left(K_1+\phi\left(\tau_1\right)\sqrt{\frac{1-f_j}{f_j}}V_j^T\beta\right).
\end{aligned}$$

With the same arguments, we have

$$\begin{aligned}
p_{con,1j}|\beta &= \frac{f_j}{1-K_1}\cdot\left(1-K_1-\phi\left(\tau_1\right)\sqrt{\frac{1-f_j}{f_j}}V_j^T\beta\right)\\
p_{cas,2j}|\gamma &= \frac{f_j}{K_2}\cdot\left(K_2+\phi\left(\tau_2\right)\sqrt{\frac{1-f_j}{f_j}}V_j^T\gamma\right)\\
p_{con,2j}|\gamma &= \frac{f_j}{1-K_2}\cdot\left(1-K_2-\phi\left(\tau_2\right)\sqrt{\frac{1-f_j}{f_j}}V_j^T\gamma\right).
\end{aligned}$$

Thus, we have

$$\begin{aligned}
&\mathbb{E}\left[\left(p_{cas,1j}-p_{con,1j}\right)\left(p_{cas,2\zeta}-p_{con,2\zeta}\right)\right]\\
&=\mathbb{E}\left[\mathbb{E}\left[\left(p_{cas,1j}-p_{con,1j}\right)\left(p_{cas,2\zeta}-p_{con,2\zeta}\right)|\beta,\gamma\right]\right]\\
&=\mathbb{E}\left[\mathbb{E}\left[p_{cas,1j}|\beta\right]\mathbb{E}\left[p_{cas,2\zeta}|\gamma\right]\right]+\mathbb{E}\left[\mathbb{E}\left[p_{con,1j}|\beta\right]\mathbb{E}\left[p_{con,2\zeta}|\gamma\right]\right]\\
&-\mathbb{E}\left[\mathbb{E}\left[p_{cas,1j}|\beta\right]\mathbb{E}\left[p_{con,2\zeta}|\gamma\right]\right]-\mathbb{E}\left[\mathbb{E}\left[p_{con,1j}|\beta\right]\mathbb{E}\left[p_{cas,2\zeta}|\gamma\right]\right]\\
&=\mathbb{E}\left[\left(f_j+\phi\left(\tau_1\right)\sqrt{\left(1-f_j\right)f_j}V_j^T\beta\frac{1}{K_1}\right)\left(f_\zeta+\phi\left(\tau_2\right)\sqrt{\left(1-f_\zeta\right)f_\zeta}V_\zeta^T\gamma\frac{1}{K_2}\right)\right]\\
&+\mathbb{E}\left[\left(f_j-\phi\left(\tau_1\right)\sqrt{\left(1-f_j\right)f_j}V_j^T\beta\frac{1}{1-K_1}\right)\left(f_\zeta-\phi\left(\tau_2\right)\sqrt{\left(1-f_\zeta\right)f_\zeta}V_\zeta^T\gamma\frac{1}{1-K_2}\right)\right]\\
&-\mathbb{E}\left[\left(f_j+\phi\left(\tau_1\right)\sqrt{\left(1-f_j\right)f_j}V_j^T\beta\frac{1}{K_1}\right)\left(f_\zeta-\phi\left(\tau_2\right)\sqrt{\left(1-f_\zeta\right)f_\zeta}V_\zeta^T\gamma\frac{1}{1-K_2}\right)\right]\\
&-\mathbb{E}\left[\left(f_j-\phi\left(\tau_1\right)\sqrt{\left(1-f_j\right)f_j}V_j^T\beta\frac{1}{1-K_1}\right)\left(f_\zeta+\phi\left(\tau_2\right)\sqrt{\left(1-f_\zeta\right)f_\zeta}V_\zeta^T\gamma\frac{1}{K_2}\right)\right]\\
&=\frac{\sqrt{f_j\left(1-f_j\right)}\sqrt{f_\zeta\left(1-f_\zeta\right)}\phi\left(\tau_1\right)\phi\left(\tau_2\right)\rho}{mK_1\left(1-K_1\right)K_2\left(1-K_2\right)}V_j^TV_\zeta.
\end{aligned}$$

Now we give $Cov\left(z_1,z_2\right)$ for binary traits which can be seen as a generalization of proposition 2 of LDSC[2].

Proposition 5 *Under the liability model above, we have*

$$\begin{aligned}
Cov\left(z_1,z_2\right)&=\frac{\sqrt{n_1n_2}}{m}\rho_{obs}V^2+\sqrt{n_1n_2P_1\left(1-P_1\right)P_2\left(1-P_2\right)}\\
&\times\left(\frac{N_{cas,cas}}{N_{cas,1}N_{cas,2}}+\frac{N_{con,con}}{N_{con,1}N_{con,1}}-\frac{N_{cas,con}}{N_{cas,1}N_{con,1}}-\frac{N_{con,cas}}{N_{con,1}N_{cas,1}}\right)V,
\end{aligned}$$

where ρ_{obs} denotes the covariance on the observed scale:

$$\rho_{obs}=\rho\left(\frac{\phi\left(\tau_1\right)\phi\left(\tau_2\right)\sqrt{P_1\left(1-P_1\right)P_2\left(1-P_2\right)}}{K_1\left(1-K_1\right)K_2\left(1-K_2\right)}\right)$$

and $N_{a,k}$ denotes the sample size of phenotype a in study k. $N_{a,b}$ denotes the overlapped sample with phenotype a in study 1 and phenotype b in study 2.

Proof. By proposition 3, we have $\mathbb{E}\left[z_1\right]=\mathbb{E}\left[z_2\right]=0$. So, $Cov\left(z_{1j},z_{2\zeta}\right)=\mathbb{E}\left[z_{1j}z_{2\zeta}\right]$. We have

$$\mathbb{E}\left[z_{1j}z_{2\zeta}\right]=\mathbb{E}\left[\frac{\sqrt{n_1n_2P_1P_2\left(1-P_1\right)\left(1-P_2\right)}\left(\hat{p}_{cas,1j}-\hat{p}_{con,1j}\right)\left(\hat{p}_{cas,2\zeta}-\hat{p}_{con,2\zeta}\right)}{\sqrt{\hat{p}_{1j}\hat{p}_{2\zeta}\left(1-\hat{p}_{1j}\right)\left(1-\hat{p}_{2\zeta}\right)}}\right].$$

By omitting an error term with order $\mathcal{O}\left(1/n\right)$, we have

$$\begin{aligned}
\mathbb{E}\left[z_{1j}z_{2\zeta}\right]&=\frac{\sqrt{n_1n_2P_1P_2\left(1-P_1\right)\left(1-P_2\right)}\mathbb{E}\left[\mathbb{E}\left[\left(\hat{p}_{cas,1j}-\hat{p}_{con,1j}\right)\left(\hat{p}_{cas,2\zeta}-\hat{p}_{con,2\zeta}\right)|\beta,\gamma\right]\right]}{\mathbb{E}\left[\sqrt{\hat{p}_{1j}\hat{p}_{2\zeta}\left(1-\hat{p}_{1j}\right)\left(1-\hat{p}_{2\zeta}\right)}\right]}\\
&\approx\frac{\sqrt{n_1n_2P_1P_2\left(1-P_1\right)\left(1-P_2\right)}\mathbb{E}\left[\mathbb{E}\left[\left(\hat{p}_{cas,1j}-\hat{p}_{con,1j}\right)\left(\hat{p}_{cas,2\zeta}-\hat{p}_{con,2\zeta}\right)|\beta,\gamma\right]\right]}{\sqrt{f_jf_\zeta\left(1-f_j\right)\left(1-f_\zeta\right)}}. \quad (19)
\end{aligned}$$

The approximation in (19) follows the arguements in (18). After conditioning on β and γ, the only source of variance in the sample allele frequencies is $\hat{p}_{cas,1}$, $\hat{p}_{con,1}$, $\hat{p}_{cas,2}$, and $\hat{p}_{con,2}$ is sample error. We can write $\hat{p}_{cas,1j}\hat{p}_{cas,2\zeta}=\left(p_{cas,2j}+\eta\right)\left(p_{cas,2\zeta}+\nu\right)$,where η and ν denote

sampling error. If there is no individual shared by study 1 and study 2, $\mathbb{E}[\eta\nu] = 0$. If study 1 and study 2 share samples, η and ν will be correlated:

$$\begin{aligned}\mathbb{E}\left[\hat{p}_{cas,1j}\hat{p}_{cas,2\zeta}|\beta,\gamma\right] &= p_{cas,1j}p_{cas,2\zeta} + \mathbb{E}[\eta\nu] \\ &\approx p_{cas,1j}p_{cas,2\zeta} + \frac{N_{cas,cas}}{N_{cas,1}N_{cas,2}}\sqrt{f_j(1-f_j)f_\zeta(1-f_\zeta)}V_{j\zeta}.\end{aligned}$$

We have similar results for other terms in (19). Finally, by plugging the results in proposition 4, we obtain

$$\begin{aligned}\mathbb{E}[z_{1j}z_{2\zeta}] =& \frac{\sqrt{n_1n_2P_1P_2(1-P_1)(1-P_2)}}{\sqrt{f_jf_\zeta(1-f_j)(1-f_\zeta)}}\left[\frac{\sqrt{f_j(1-f_j)}\sqrt{f_\zeta(1-f_\zeta)}\phi(\tau_1)\phi(\tau_2)\rho}{mK_1(1-K_1)K_2(1-K_2)}V_j^TV_\zeta\right. \\ &\left.+\sqrt{f_j(1-f_j)f_\zeta(1-f_\zeta)}V_{j\zeta}\left(\frac{N_{cas,cas}}{N_{cas,1}N_{cas,2}}+\frac{N_{con,con}}{N_{con,1}N_{con,1}}-\frac{N_{cas,con}}{N_{cas,1}N_{con,1}}-\frac{N_{con,cas}}{N_{con,1}N_{cas,1}}\right)\right] \\ =& \sqrt{n_1n_2P_1P_2(1-P_1)(1-P_2)}\left[\frac{\rho\phi(\tau_1)\phi(\tau_2)V_j^TV_\zeta}{mK_1K_2(1-K_1)(1-K_2)}\right. \\ &\left.+V_{j\zeta}\left(\frac{N_{cas,cas}}{N_{cas,1}N_{cas,2}}+\frac{N_{con,con}}{N_{con,1}N_{con,1}}-\frac{N_{cas,con}}{N_{cas,1}N_{con,1}}-\frac{N_{con,cas}}{N_{con,1}N_{cas,1}}\right)\right]\end{aligned}$$

as desired. Similar to LDSC[2] and GNOVA[4], SUPERGNOVA also estimates genetic covariance on the observed scale. With the results and arguements above, we give the following corollaries.

Corollary 1 *Under the liability model above, we denote z_{1i} and z_{2i} as the z-scores of the SNPs in region i for study 1 and study 2, respectively. Under the assumptions for local genetic covariance above, we have*

$$\begin{aligned}Cov(z_1,z_2) =& \frac{\sqrt{n_1n_2}}{m_i}\rho_{i,obs}V_i^2 + \sqrt{n_1n_2P_1(1-P_1)P_2(1-P_2)} \\ &\times\left(\frac{N_{cas,cas}}{N_{cas,1}N_{cas,2}}+\frac{N_{con,con}}{N_{con,1}N_{con,1}}-\frac{N_{cas,con}}{N_{cas,1}N_{con,1}}-\frac{N_{con,cas}}{N_{con,1}N_{cas,1}}\right)V_i,\end{aligned}$$

where $\rho_{i,obs}$ is the local genetic covariance on observed scale:

$$\rho_{i,obs} = \rho_i\left(\frac{\phi(\tau_1)\phi(\tau_2)\sqrt{P_1(1-P_1)P_2(1-P_2)}}{K_1(1-K_1)K_2(1-K_2)}\right).$$

Corollary 2 *Similar to the corollary 2 in LDSC[2], SUPERGNOVA framework can also be generated to estimate genetic covariance between binary traits and quantitative traits. If study 1 is a binary trait, and study 2 is a quantitative study, proposition 5 holds with genetic covariance on the half-observed scale:*

$$\rho_{obs} = \rho_g\left(\frac{\phi(\tau_1)\sqrt{P_1(1-P_1)}}{K_1(1-K_1)}\right).$$

4 Additional simulations

4.1 UK Biobank (UKBB) genotype data

We have used non-imputed genotype data from the Wellcome Trust Case Control Consortium (WTCCC) to conduct simulations to compare the performance of different global/local genetic covariance estimation methods. Here we introduce a new set of simulation which extended our

previous settings to densely imputed data from the UK Biobank (UKBB). Unlike ρ-HESS[5], we used real genotype data from WTCCC and UKBB as the GWAS samples while 1000 Genome Project samples were used as the external LD reference panel[3].

We used phase 3 genotype data released by UKBB and restricted the analysis to autosomal variants with genotype missing rate per marker <0.01, imputation quality score > 0.3, Hardy-Weinberg Equilibrium p-value > 1e-9, and minor allele frequency (MAF) > 0.1%. We also excluded SNPs not present in the 1000 Genomes (which is our LD reference panel). We randomly selected n=20,000 independent subjects with European ancestry for the simulation. We divided the samples into two equal-sized groups.

Similar to the **Simulation settings** section of the main text, SNPs on chromosome 2 were set as the background variants, and the heritability values for both traits were fixed as 0.5. There are 406,785 SNPs on chromosome 2 after quality control. We used the same region in the main text as the local region of interest (chromosome 2: 176,998,822-180,334,969). There are 5,667 SNPs in this region. The covariance of the local genetic effects was set to be 0, 0.001, 0.002, 0.003, 0.004, and 0.005, respectively.

We obtained highly consistent results in these new simulations (**Supplementary Figure 10**). SUPERGNOVA achieved lower bias and improved statistical power with well-calibrated type I error. The density of SNPs did not show much influence on the performance of SUPERGNOVA.

4.2 Model mis-specification

We investigate the robustness of SUPERGNOVA under various settings of model mis-specification in this section. We used the same genotype data from the WTCCC introduced in **Simulation settings** section. The phenotypes were simulated for two randomly partitioned sample subgroups (N=7,959 in each set). All SNPs in chromosome 2 were set as background SNPs and the largest region in chromosome 2 (chr2: 176,998,822-180,334,969) was set to be the local region of interest.

4.2.1 10% causal SNPs

We first investigated the robustness of SUPERGNOVA against sparse genetic architecture with fewer causal SNPs. In each replicate of 100, We randomly selected 10% of SNPs inside and outside of the region of interest as the causal SNPs, respectively. Phenotypes of the two traits are generated based on the randomly selected causal SNPs. The true local genetic covariance values were set to be 0 and 0.005 and the heritability of both traits is fixed as 0.5. Genetic covariance and heritability were evenly distributed among all causal SNPs. When estimating local genetic covariance, we did not use information about causal SNP assignment and still fitted an (locally) infinitesimal model as proposed in SUPERGNOVA. The results are shown in **Supplementary Figure 11**. SUPERGNOVA is robust to the sparse setting even when model was mis-specified and achieved comparable results with a slight reduction in statistical power.

4.2.2 Minor allele frequency (MAF)

In SUPERGNOVA framework, we assume effect sizes for normalized genotype are not related with MAF. Here, we investigated the performance of SUPERGNOVA when the SNP effect size is affected by MAF which we denote as f_j. Specifically, we assume the variance and covariance of the effect size of SNP j are proportional to $f_j(1-f_j)$. That is to say $\beta_j \sim N(0, h_1^2[f_j(1-f_j)/\sigma])$ and $\gamma_j \sim N(0, h_2^2[f_j(1-f_j)/\sigma])$, where $\sigma = \sum f_j(1-f_j)$. And for the SNPs in the region of interest, $cov(\beta_j, \gamma_j) = \rho[f_j(1-f_j)/\sigma_i]$, where σ_i is the sum of $f_j(1-f_j)$ for all the SNPs in region i. This kind of assumption has been used in some other literatures[1]. Namely, the per SNP heritability and genetic covariance are the same if the genotypes are not normalized.

The results are shown in **Supplementary Figure 12**. The point estimates of local genetic covariance remained unbiased but we observed some inflation in the type-I error rate. This motivates us to propose a more general model that fits into different relation between MAF and genetic effects in the future work.

4.2.3 LDscore

Finally, we simulated the effect sizes to be inversely proportional to LD score. The results of point estimate are shown in **Supplementary Figure 13**. We did not observe any inflation in the type-I error rate. No replicates had p-values below 0.05 when the true genetic covariance was 0. There was substantial underestimation of local genetic covariance when the true genetic covariance was set to be 0.005. Only one replicate had a p-value below 0.05. Overall, these results suggest that SUPERGNOVA is too conservative under a mis-specified model with LD-dependent genetic effects.

4.3 Window size

SUPERGNOVA is a flexible statistical framework that estimates local genetic covariance in any pre-specified genomic region. How the window size will affect SUPERGNOVAs performance depends on the true (shared) genetic architecture of two phenotypes.

We started from the same region from WTCCC used in the simulations described in the **Simulation settings** section of the main text(chromosome 2: 176,998,822-180,334,969; 3.3Mb). Then, we expanded the size of the region to 6.8Mb (174,472,685-181,308,939), 8.8Mb (172,946,315-181,794,257), 12.2Mb (170,996,943-183,183,026), and 14.8Mb (169,568,977-184,356,242). We simulated data in two different scenarios to study the performance of SUPERGNOVA:

1) The effect sizes of the SNPs are correlated in the single region chr2: 176,998,822-180,334,969. The local genetic covariance is set to be 0.005. The genetic covariances of the SNPs outside the region are zero. This simulation setting represents a scenario where expanding the window size simply adds noise (with 0 genetic covariance) into the estimation.

2) The effect sizes of the SNPs are correlated in the entire chromosome 2. The global genetic covariance is set to be 0.25. This simulation setting mimics the genetic architecture where genetic covariance is ubiquitous in the genome. Under this scenario, expanding the window size will add to the total genetic covariance in the window.

Similar to the **simulation settings** of the main text, SNPs on chromosome 2were set as the background SNP and the heritability values for both traits are fixed as 0.5. We estimated the local genetic covariances of the regions with different sizes. **Supplementary Figure 14** shows the trend of statistical power as the window size increases in two scenarios.

We can observe that when the SNP effects are correlated locally, statistical power decreases as the window expands. When the SNP effects are correlated globally, the power increases as the window expands. However, we note that the argument about how to define window size is not entirely statistical. When two traits have highly polygenic genetic sharing, analysis based on a large window size will most likely improve statistical power. Still, smaller window size will help“fine-map” the local correlational patterns and identify precise genomic components that contribute to the shared genetic basis between complex traits. Our implemented SUPERGNOVA software allows users to specify the genomic region of interest and will estimate local genetic covariance accordingly.

4.4 Applications of LDSC on local genetic correaltion estimation

Following the simulations for WTCCC without sample overlap, we directly applied LDSC on ocal genetic correlation estimation. The results showed that LDSC showed severe type-I error

inflation when estimating local genetic correlation. The distribution of the p-values under the true local genetic correlation equal to zero is shown in **Supplementary Figure 15**.

5 Interpretability of local genetic covariance

Although global genetic correlation is a desirable metric, local genetic correlation estimates can be very noisy and difficult to interpret. We detail this argument here by theoretical and numerical demonstrations.

Genetic correlation is defined as $\rho/\sqrt{h_1^2 h_2^2}$, where ρ denote genetic covariance and h_1^2 and h_2^2 denote the heritability of trait 1 and trait 2. In local genetic correlation, ρ, h_1^2, and h_2^2 are replaced by the local genetic covariance and local heritability, respectively. To simplify the notation, we use ρ to denote the estimated genetic covariance and h to denote the estimation of $\sqrt{h_1^2 h_2^2}$ in this section. Then, the estimate of genetic correlation is given by ρ/h. We denote the standard error of ρ and h as σ_1 and σ_2, respectively. The covariance between ρ and h is r. By delta methods, the standard error ρ/h is given by

$$se(\rho/h) = \sqrt{\sigma_1^2/h^2 + \sigma_2^2\rho^2/h^4 - 2r\rho/h^3}.$$

The SNP heritability in a local genomic region is often very small compared to its estimation standard error, which makes the estimation of genetic correlation ρ/h very noisy.

We illustrate this using numerical analysis. We selected two sets of genetic covariance and heritability values to mimic the magnitude of parameters in global and local genetic correlation analyses. In the first setting, we set the true value of genetic covariance as 0.25 and heritability as 0.5. In the second setting, we set the true value of genetic covariance as 0.05 and heritability as 0.1. The genetic correlation is 0.5 for both settings. Here, for simplicity, the term heritability refers to $\sqrt{h_1^2 h_2^2}$. The standard error of all the estimates are 0.03. We assume the estimates of genetic covariance and heritability are independent. We set the standard error of genetic covariance to be the same in two scenarios because global and local genetic covariances have comparable standard errors in practice. For each setting, we randomly sample (ρ, h) as follows:

1) $\rho \sim N(0.25, 0.03^2)$ and $h \sim N(0.5, 0.03^2)$;

2) $\rho \sim N(0.05, 0.03^2)$ and $h \sim N(0.1, 0.03^2)$.

The comparison of genetic correlation estimates (i.e., ρ/h) is shown in **Supplementary Figure 1**. We observed substantially noisier estimates in setting-2 where the magnitude of genetic covariance and heritability is small.

References

[1] B. C. Brown, C. J. Ye, A. L. Price, N. Zaitlen, A. G. E. N. T. . D. Consortium, et al. Transethnic genetic-correlation estimates from summary statistics. *The American Journal of Human Genetics*, 99(1):76–88, 2016.

[2] B. Bulik-Sullivan, H. K. Finucane, V. Anttila, A. Gusev, F. R. Day, P.-R. Loh, L. Duncan, J. R. Perry, N. Patterson, E. B. Robinson, et al. An atlas of genetic correlations across human diseases and traits. *Nature genetics*, 47(11):1236, 2015.

[3] . G. P. Consortium et al. An integrated map of genetic variation from 1,092 human genomes. *Nature*, 491(7422):56–65, 2012.

[4] Q. Lu, B. Li, D. Ou, M. Erlendsdottir, R. L. Powles, T. Jiang, Y. Hu, D. Chang, C. Jin, W. Dai, et al. A powerful approach to estimating annotation-stratified genetic covariance via gwas summary statistics. *The American Journal of Human Genetics*, 101(6):939–964, 2017.

[5] H. Shi, N. Mancuso, S. Spendlove, and B. Pasaniuc. Local genetic correlation gives insights into the shared genetic architecture of complex traits. *The American Journal of Human Genetics*, 101(5):737–751, 2017.
