# Supplementary material for: SUPERGNOVA: local genetic correlation analysis reveals heterogeneous etiologic sharing of complex traits
Source: Genome Biol. 2021 Sep 7;22:262. doi: 10.1186/s13059-021-02478-w (PMC8422619; doi:10.1186/s13059-021-02478-w)

Supplementary Figures

Supplementary Figure 1. Estimates of genetic correlation when the magnitude of genetic covariance is different. The genetic correlation is fixed as 0.5. The genetic covariance varies from 0.25 to 0.05. The red dashed represents the true value of genetic correlation.

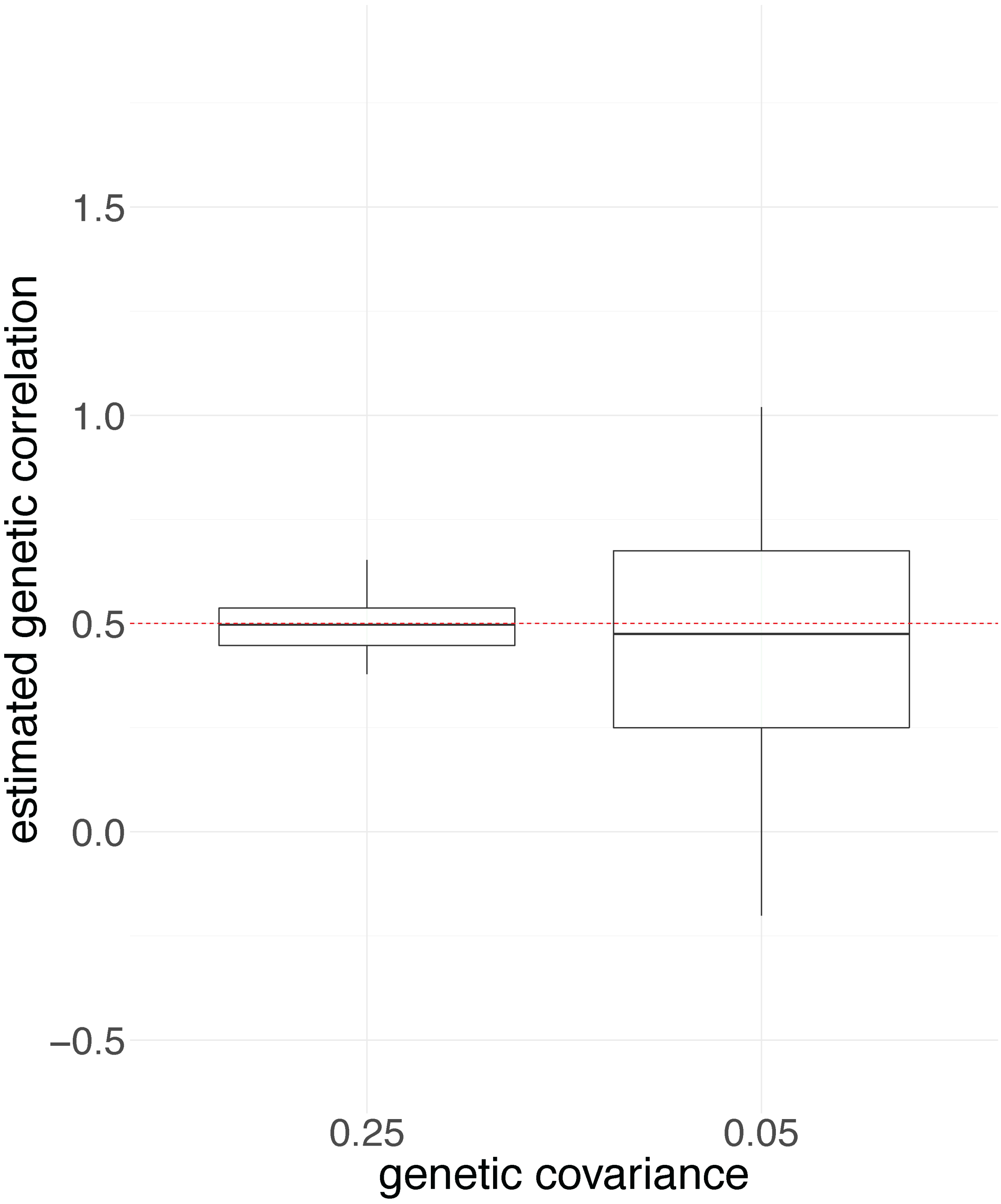

Supplementary Figure 2. Evaluation of global covariance estimation and type I error control on non-overlapping datasets (set 1 and set 2). (A) Comparison of global genetic covariance estimates among LDSC, GNOVA and SUPERGNOVA demonstrated by boxplot which shows the quantiles of the estimates. The red dashed lines represent true value of genetic covariance. **(B)** Comparison of global genetic covariance estimates among LDSC, GNOVA and SUPERGNOVA by means and the corresponding confidence intervals **(C)** qq-plot of p value when the true global genetic covariance is set to be 0.

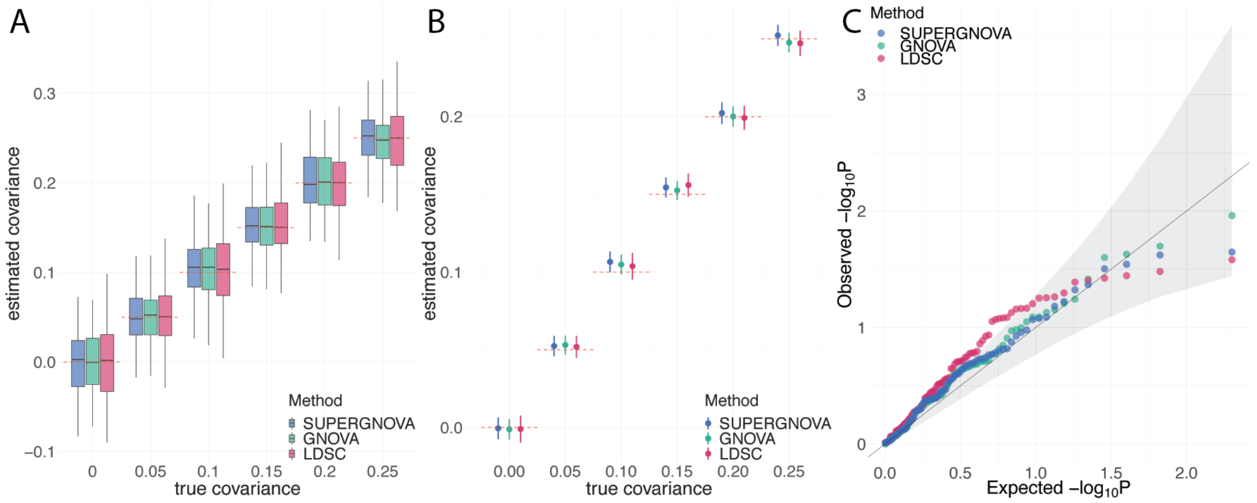

Supplementary Figure 3. Evaluation of global covariance estimation and type I error control on partially-overlapping datasets (set 1 and set 3). (A) Comparison of global genetic covariance estimates among LDSC, GNOVA and SUPERGNOVA demonstrated by boxplot which shows the quantiles of the estimates. The red dashed lines represent true value of genetic covariance. **(B)** Comparison of global genetic covariance estimates among LDSC, GNOVA and SUPERGNOVA by means and the corresponding confidence intervals **(C)** qq-plot of p value when the true global genetic covariance is set to be 0.

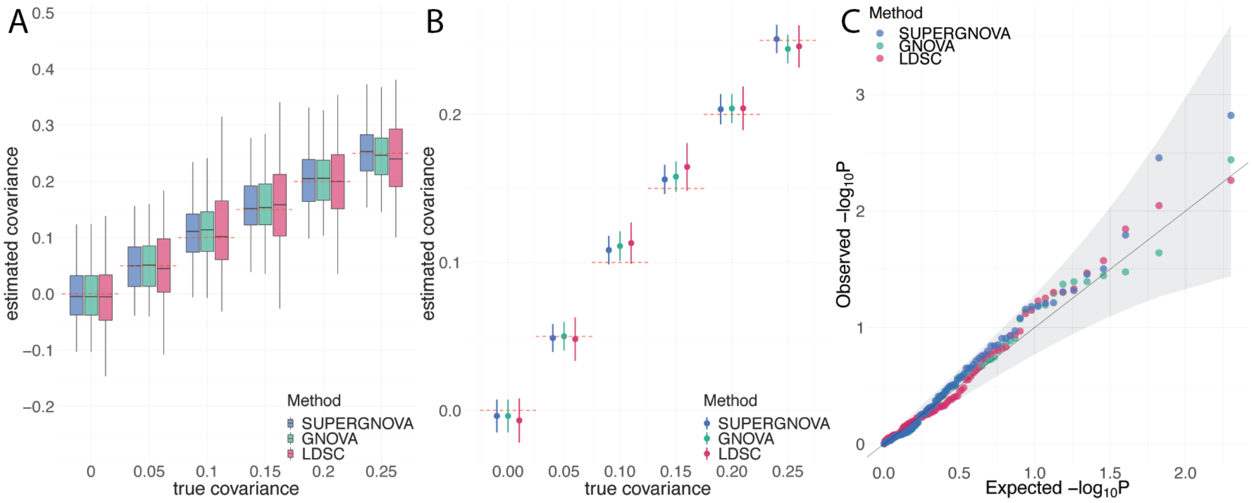

Supplementary Figure 4. Evaluation of global covariance estimation and type I error control on a completely-overlapping dataset (set 1). (A) Comparison of global genetic covariance estimates among LDSC, GNOVA and SUPERGNOVA demonstrated by boxplot which shows the quantiles of the estimates. The red dashed lines represent true value of genetic covariance. **(B)** Comparison of global genetic covariance estimates among LDSC, GNOVA and SUPERGNOVA by means and the corresponding confidence intervals **(C)** qq-plot of p value when the true global genetic covariance is set to be 0.

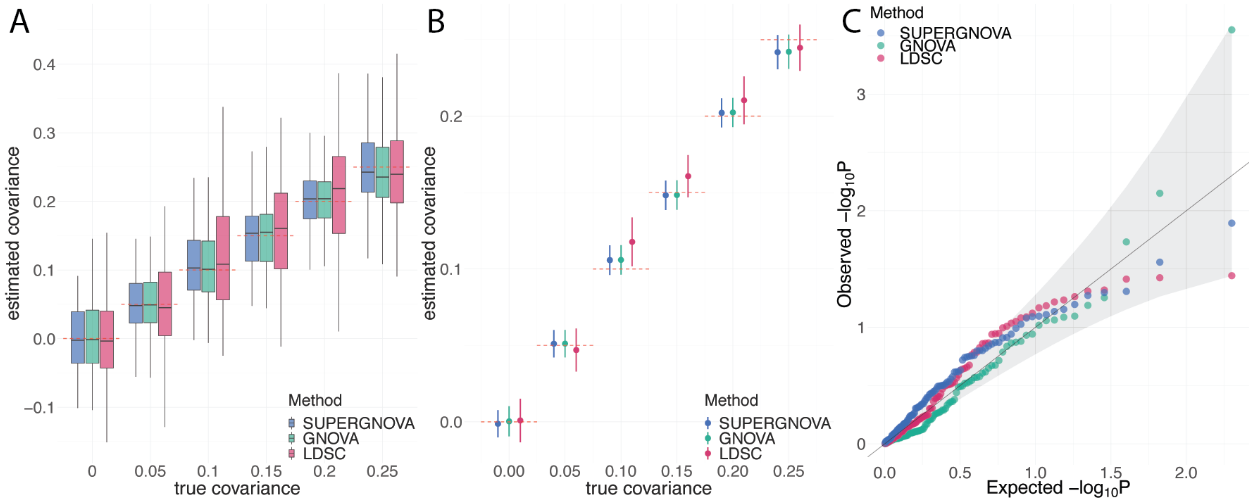

Supplementary Figure 5. Evaluation of local covariance estimation and type I error control on non-overlapping datasets (set 1 and set 2). (A) Comparison of local genetic covariance estimates between SUPERGNOVA and ρ-HESS demonstrated by boxplot which shows the quantiles of the estimates. The red dashed lines represent true value of genetic covariance. **(B)** Comparison of local genetic covariance estimates between SUPERGNOVA and ρ-HESS by means and the corresponding confidence intervals **(C)** qq-plot of p value when the true local genetic covariance is set to be 0.

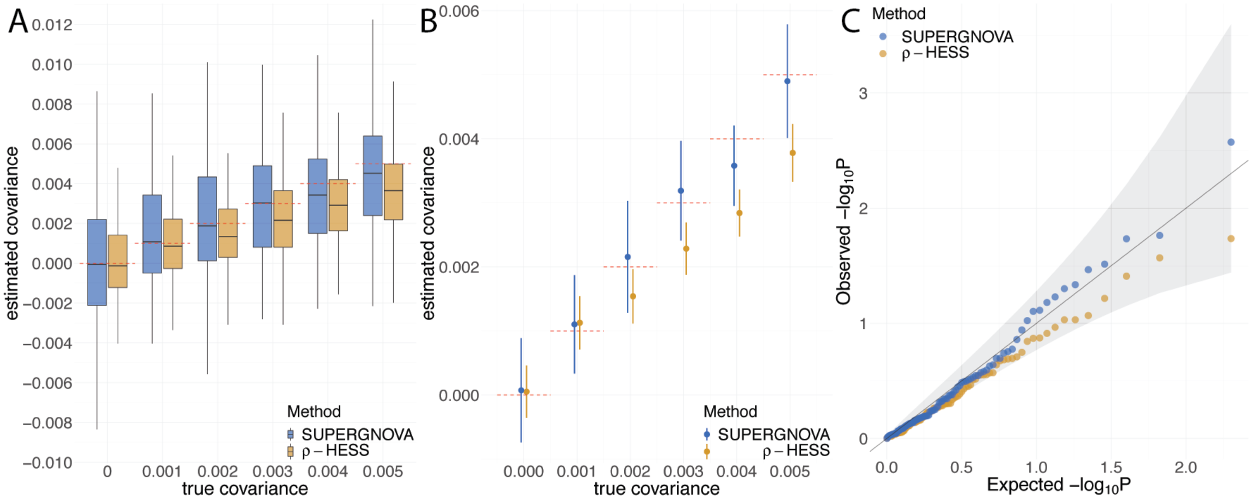

Supplementary Figure 6. Evaluation of local covariance estimation and type I error control on partially-overlapping datasets (set 1 and set 3). (A) Comparison of local genetic covariance estimates between SUPERGNOVA and ρ-HESS demonstrated by boxplot which shows the quantiles of the estimates. The red dashed lines represent true value of genetic covariance. **(B)** Comparison of local genetic covariance estimates between SUPERGNOVA and ρ-HESS by means and the corresponding confidence intervals **(C)** qq-plot of p value when the true local genetic covariance is set to be 0.

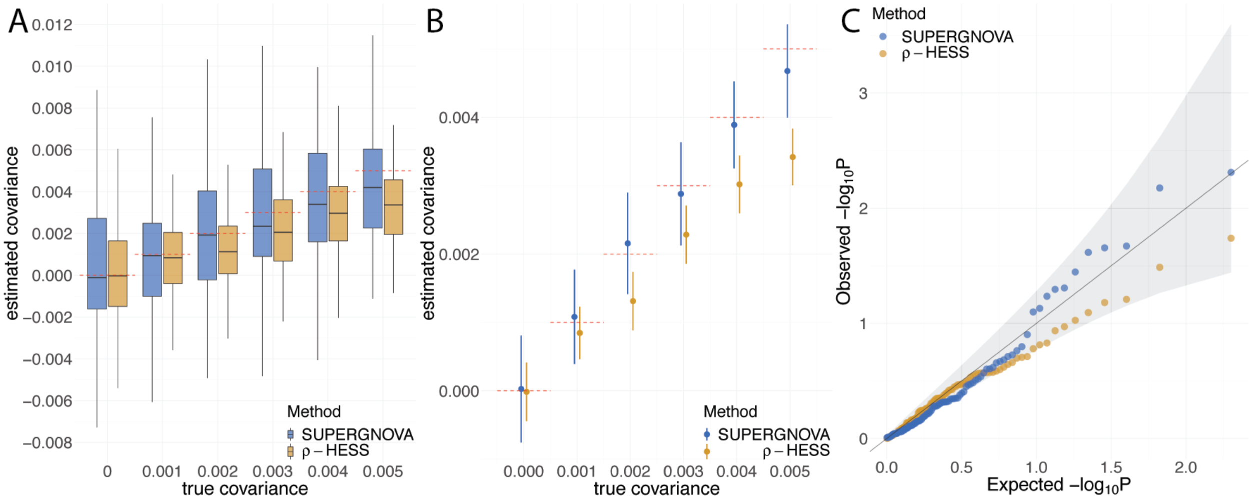

Supplementary Figure 7. Evaluation of local covariance estimation and type I error control on a completely-overlapping dataset (set 1). (A) Comparison of local genetic covariance estimates between SUPERGNOVA and ρ-HESS demonstrated by boxplot which shows the quantiles of the estimates. The red dashed lines represent true value of genetic covariance. **(B)** Comparison of local genetic covariance estimates between SUPERGNOVA and ρ-HESS by means and the corresponding confidence intervals **(C)** qq-plot of p value when the true local genetic covariance is set to be 0.

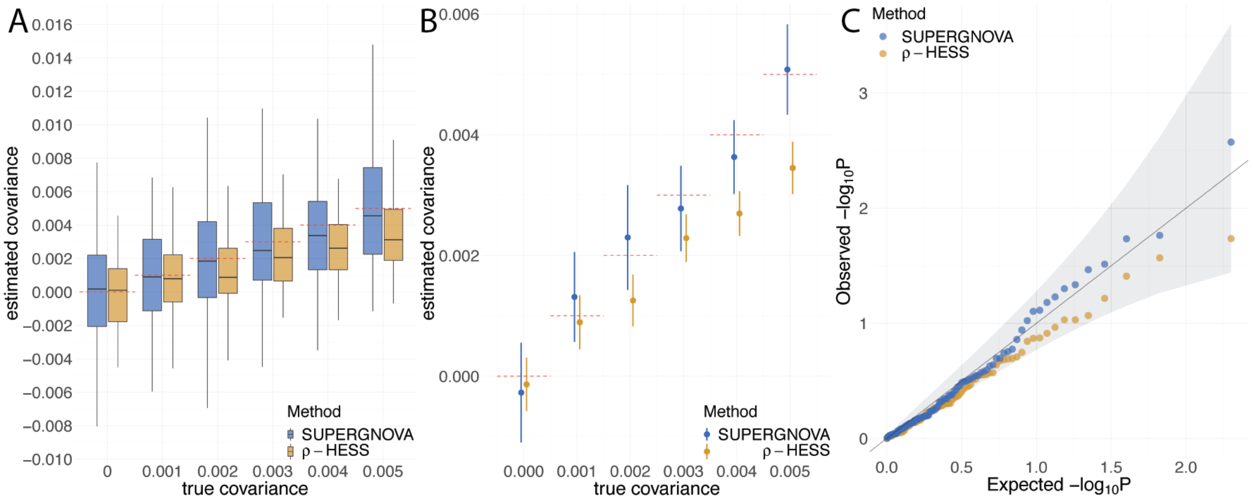

Supplementary Figure 8. Evaluation of the performance of ρ-HESS when provided with inaccurate overlapping sample size on a partially-overlapping datasets (set 1 and set 3). (A) local genetic covariance estimates. The red dashed lines represent true value of genetic covariance. **(B)** type-I error and statistical power (C) qq-plot of p value when the true local genetic covariance is set to be 0.

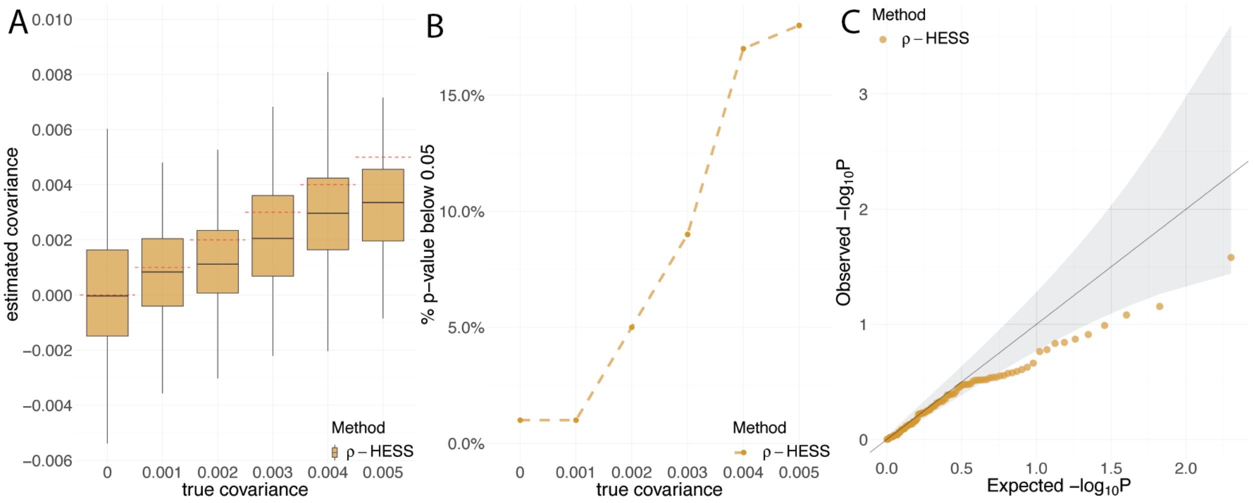

Supplementary Figure 9. Evaluation of the performance of ρ-HESS when provided with inaccurate overlapping sample size on a completely-overlapping dataset (set 1). (A) local genetic covariance estimates. The red dashed lines represent true value of genetic covariance. **(B)** type-I error and statistical power (C) qq-plot of p value when the true local genetic covariance is set to be 0.

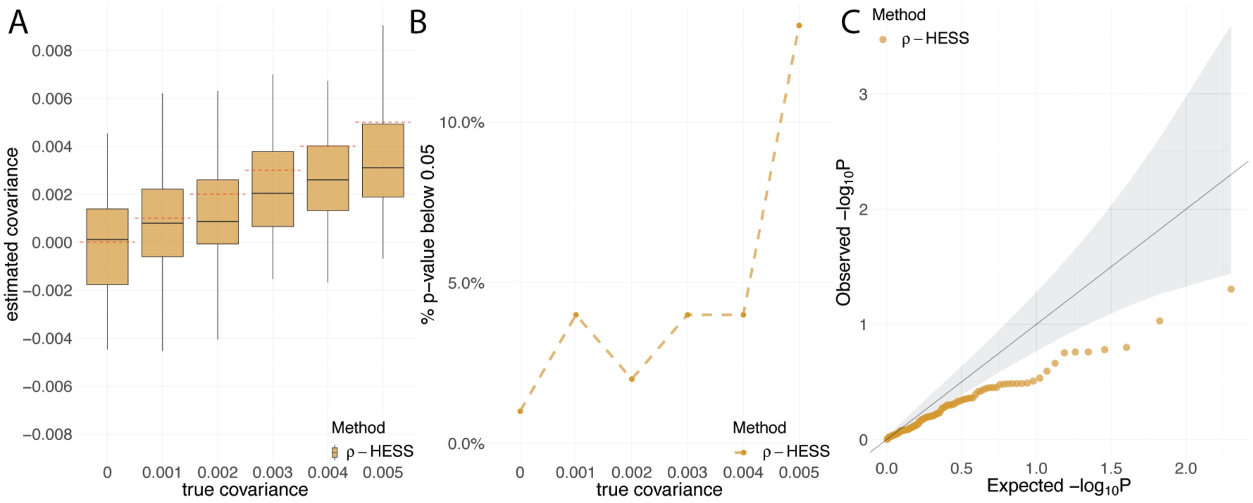

Supplementary Figure 10. Evaluation of local covariance estimation, statistical power, and type I error control on UKBB datasets. (A) Comparison of local genetic covariance estimates between SUPERGNOVA and demonstrated by boxplot which shows the quantiles of the estimates. The red dashed lines represent true value of genetic covariance. **(B)** Comparison of local genetic covariance estimates between SUPERGNOVA and ρ-HESS by means and the corresponding confidence intervals **(C)** Comparison of type I error and statistical power **(D)** qq-plot of p value when the true local genetic covariance is set to be 0.

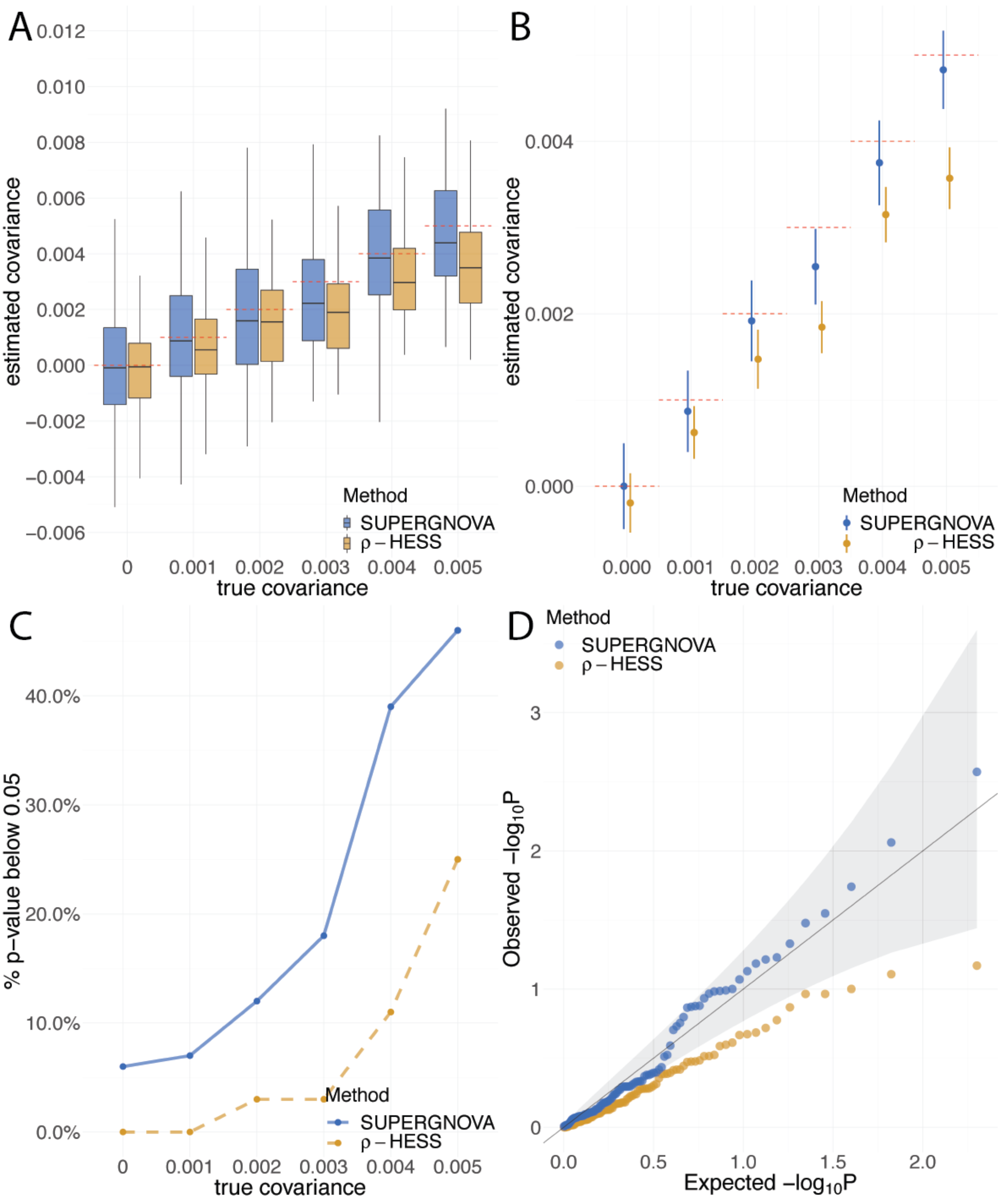

Supplementary Figure 11. SUPERGNOVA is robust under mis-specified models with sparse genetic architecture. (A) Local genetic covariance estimation. The red dashed lines represent true value of genetic covariance. **(B)** Type I error and statistical power **(C)** qq-plot of p value when the true local genetic covariance is set to be 0.

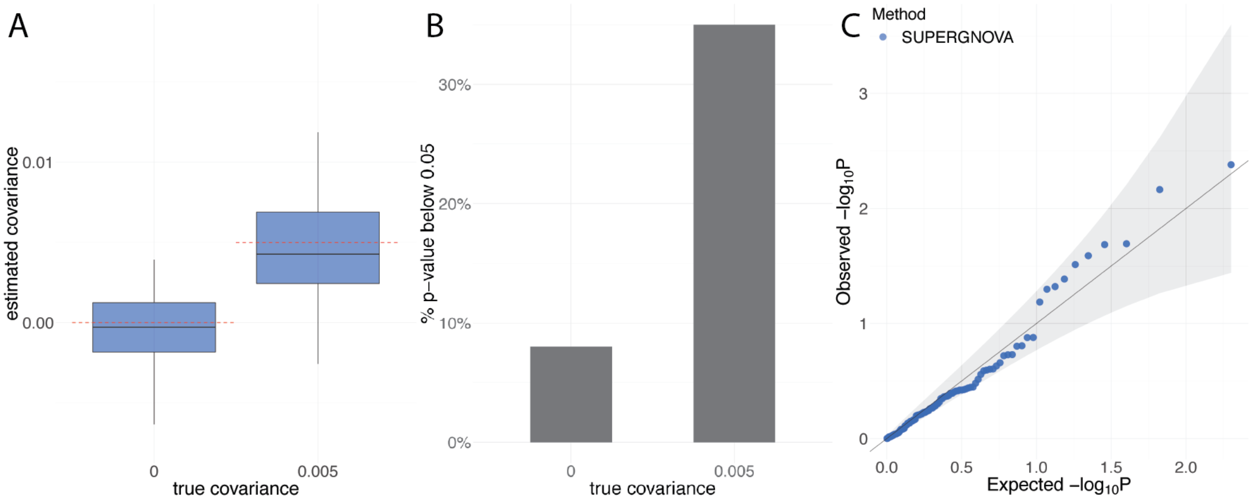

Supplementary Figure 12. Under a mis-specified model assuming MAF-dependent genetic effects, local genetic covariance estimation remains unbiased but type I error shows some inflation. (A) Local genetic covariance estimation. The red dashed lines represent true value of genetic covariance. **(B)** Type I error and statistical power **(C)** qq-plot of p value when the true local genetic covariance is set to be 0.

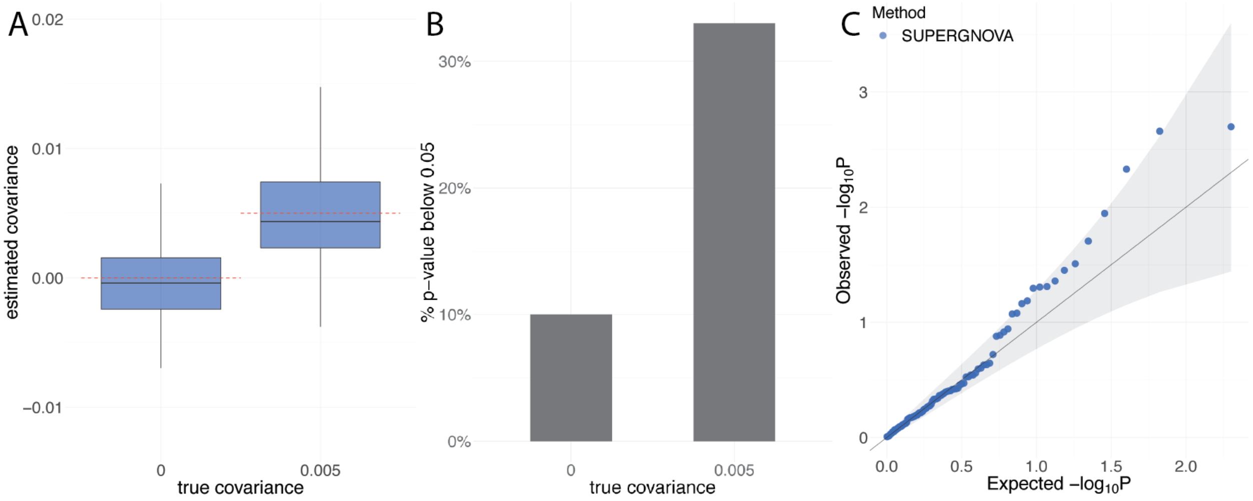

Supplementary Figure 13. Local genetic covariance estimation of SUPERGNOVA under model misspecification when effect sizes are related with LDscore. SUPERGNOVA is not robust when per SNP genetic covariance is related with LDscore. The red dashed lines represent true value of genetic covariance.

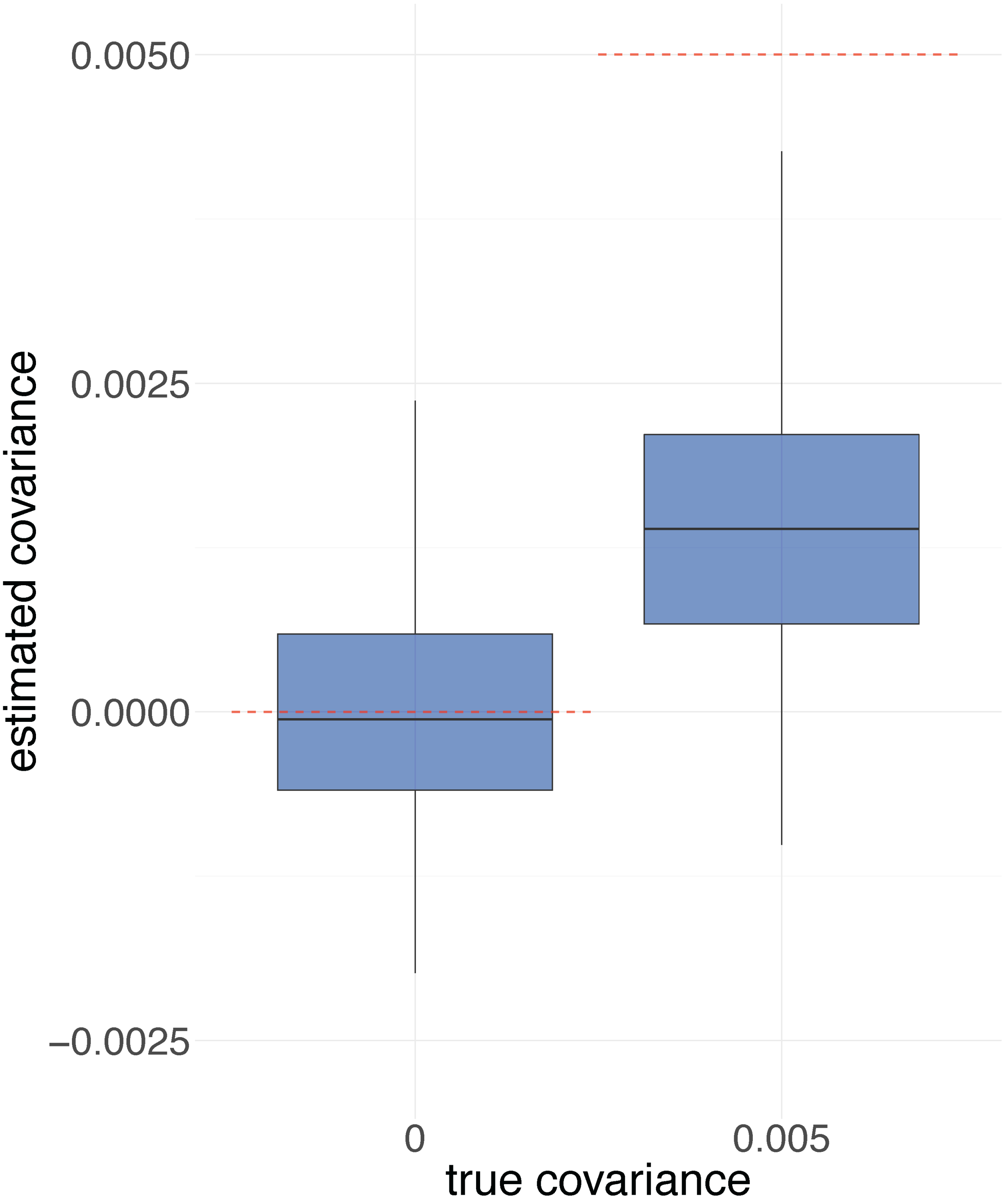

Supplementary Figure 14. The power of local genetic covariance estimation for different window sizes. (**A**) Effect sizes of the SNPs are correlated only in a 3.3Mb region. (**B**) Effect sizes of the SNPs are correlated globally.

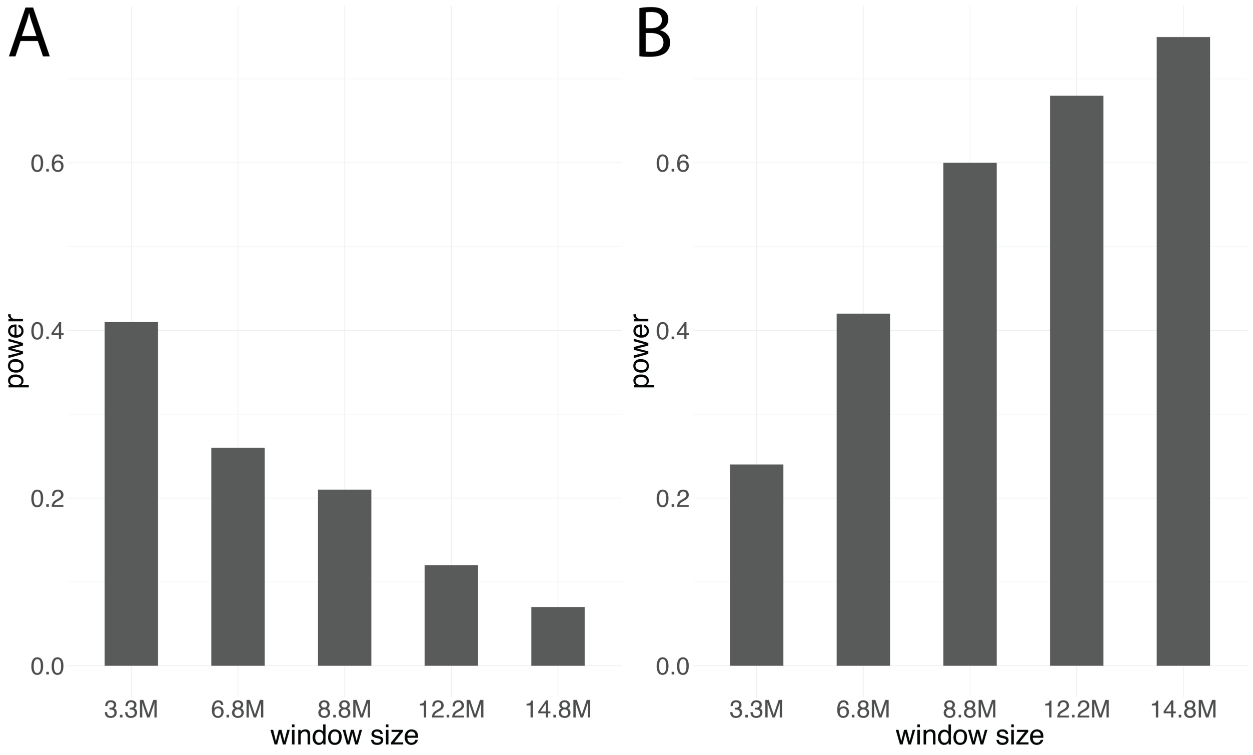

Supplementary Figure 15. LDSC suffers from type-I error inflation when directly applied to local genetic correlation estimation. We directly applied LDSC to local genetic correlation estimation. qq-plot of p value when the true global genetic covariance is set to be 0.

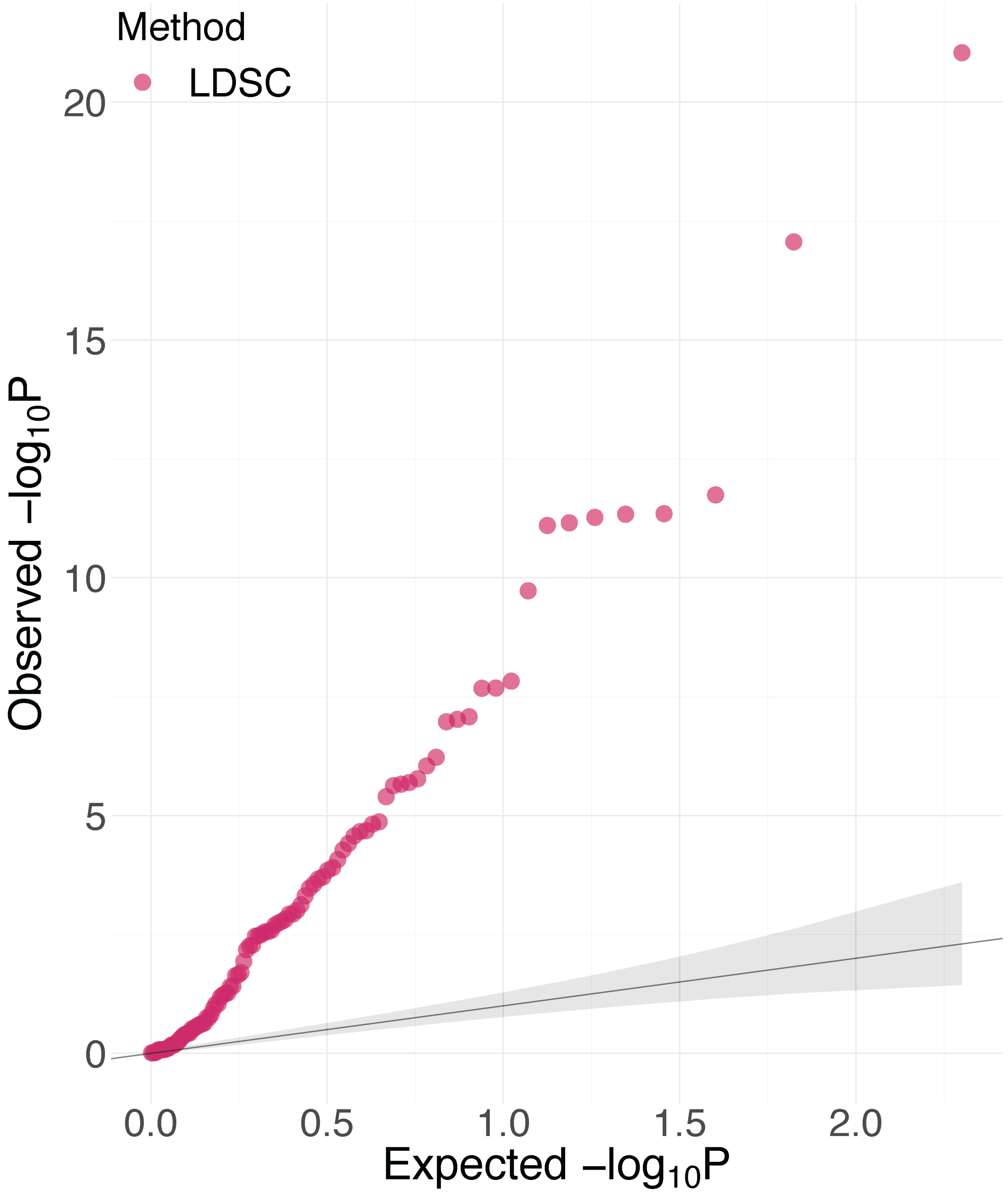

Supplementary Figure 16. Volcano plot for global genetic correlation. Each point represents a trait pair. Color of each data point represents the significance and direction of global correlation.

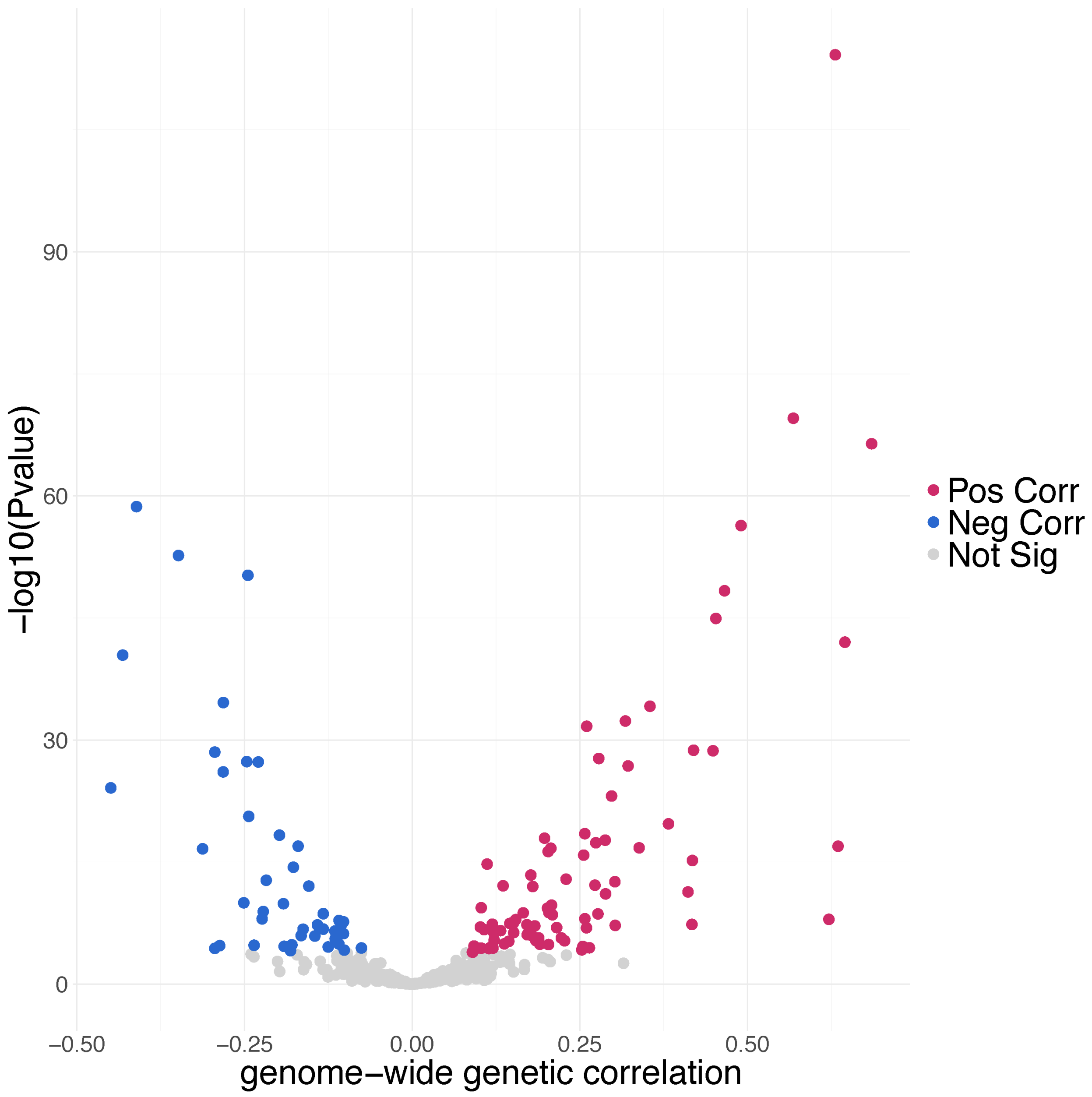

Supplementary Figure 17. Histograms of z scores of local genetic covariance. The red lines represent the density function of a standard normal distribution. The trait pairs are **(A)** Crohn-IBD, **(B)** IBD-UC, **(C)** Crohn-UC**, (D)** CP-EA, **(E)** BMI-HDL, and **(F)** ASD-CP.

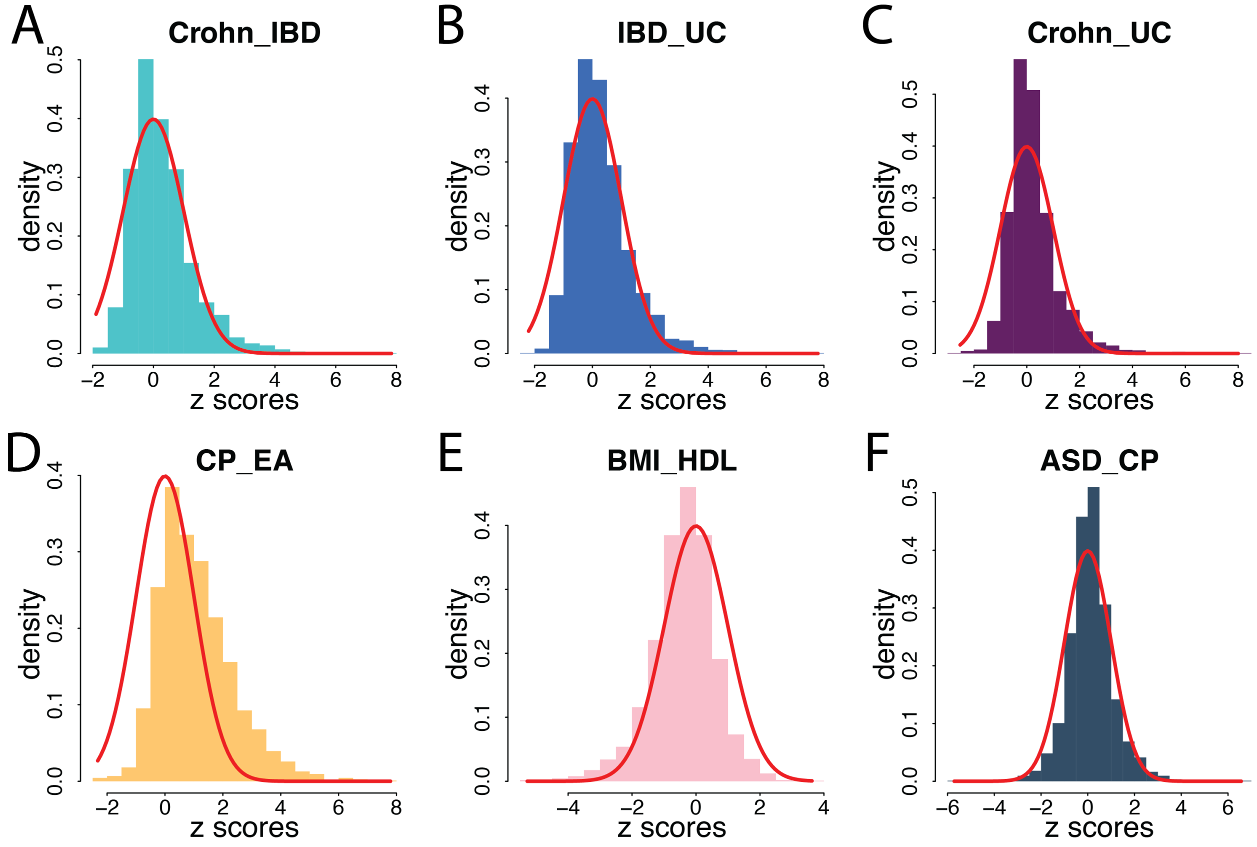

Supplementary Figure 18. The relationship between the square root of the sample size product and proportion of correlated regions. Each point represents a pair of traits. Color of each data point denotes the significance and direction of global genetic covariance. The sample size and the proportion of correlated regions showed a modest association (correlation=0.28).

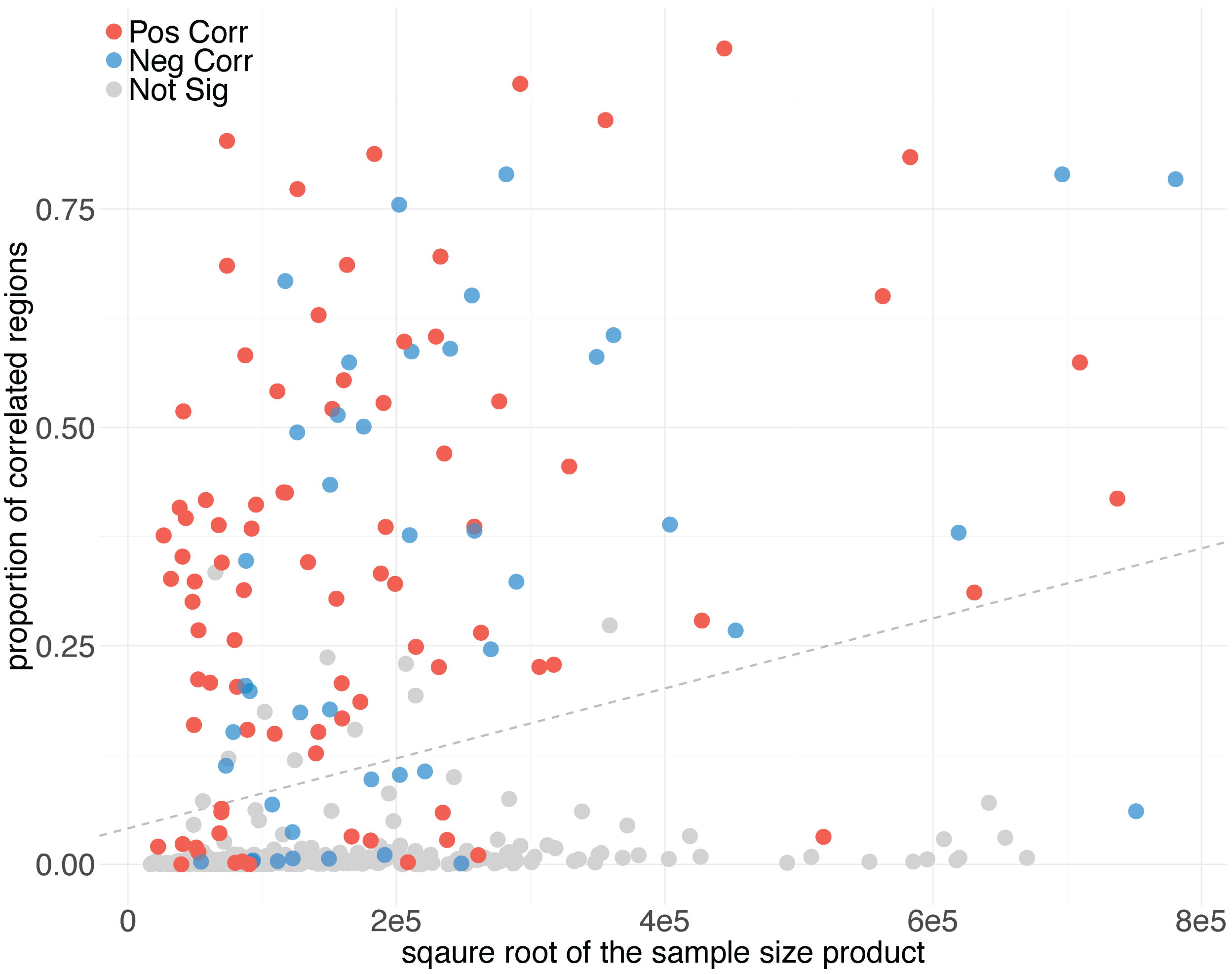

Supplementary Figure 19. Histograms of z scores of local genetic covariance. The red lines represent standard normal distribution. These trait pairs were significantly correlated in local regions but not identified as globally correlated, including **(A)** HDL-LDL, **(B)** CP-MDD, **(C)** AXD-OCD, and **(D)** ASD-BD.

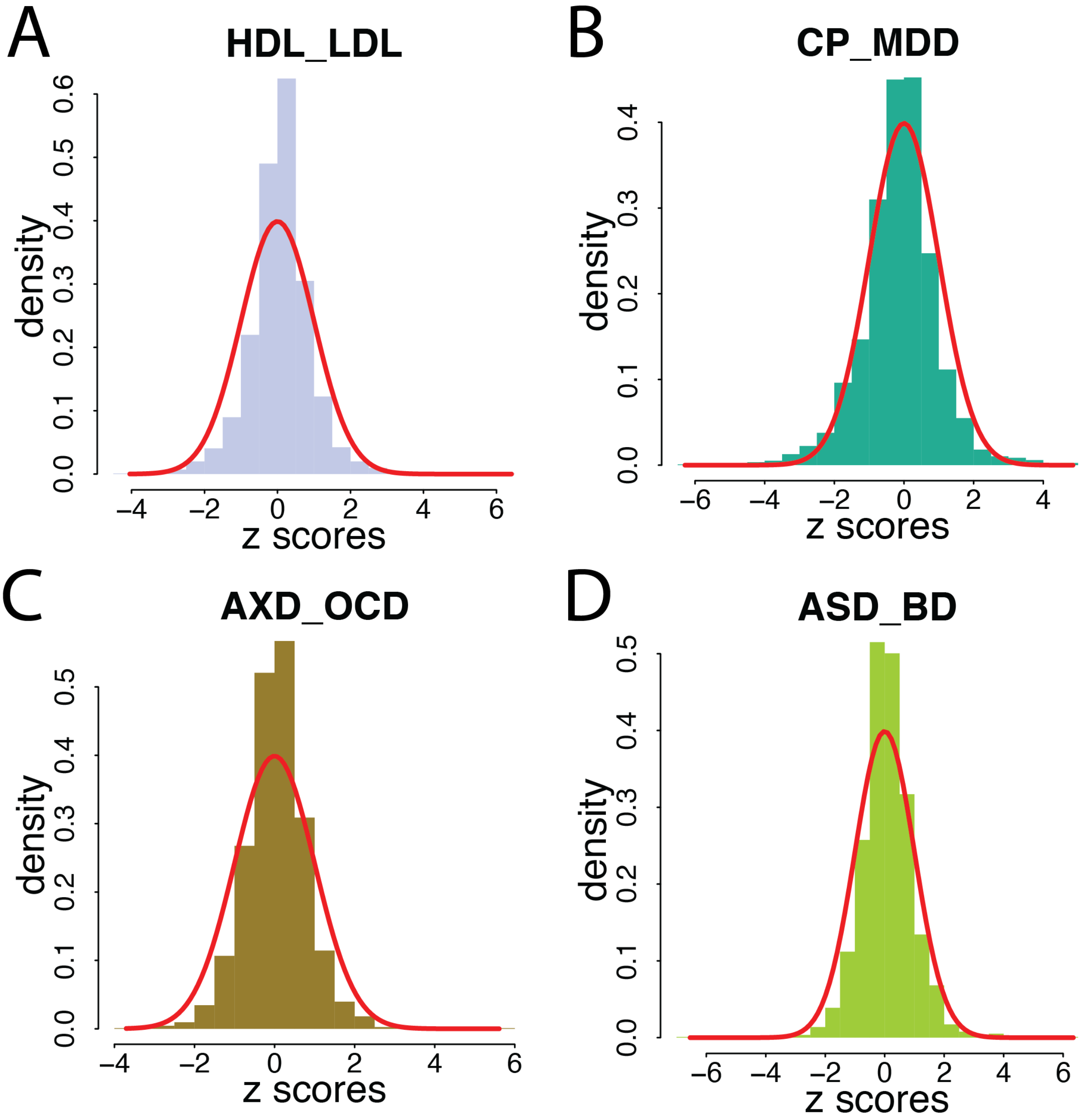

Supplementary Figure 20. LocusZoom plots for SCZ, EA, SmkInit, DrnkWk, and ADHD at the *BDNF* locus. ADHD-DrnkWk, ADHD-SCZ, ADHD-SmkInit, and DrnkWk-SCZ are positively correlated in the highlighted region and ADHD-EA and DrnkWk-EA are negatively correlated in the highlighted region.

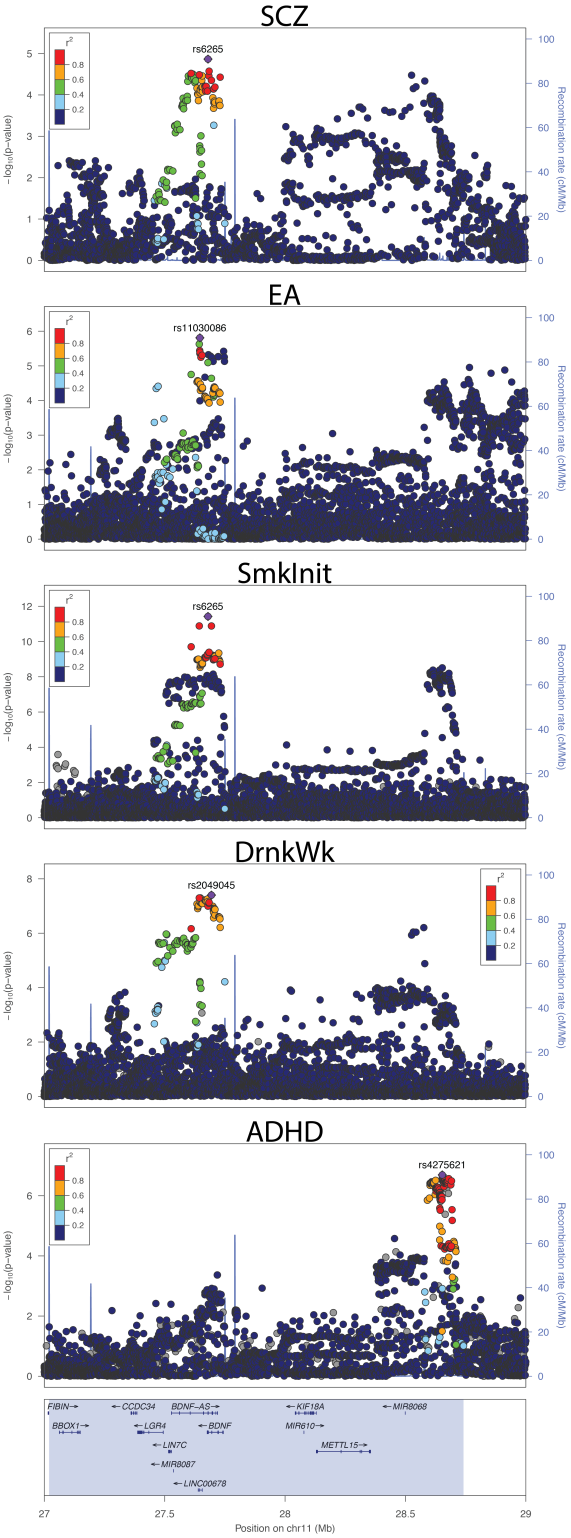

Supplementary Figure 21. Estimates of local genetic covariance among EA, ADHD, SCZ, DrnkWk, and SmkInit at the *BDNF* locus. Single asterisks highlight significant genetic covariances after Bonferroni correction for 435 pairs (among the 30 traits). Double asterisks highlight significant genetic covariances after Bonferroni correction for all 1,006,072 regions in 435 trait pairs.

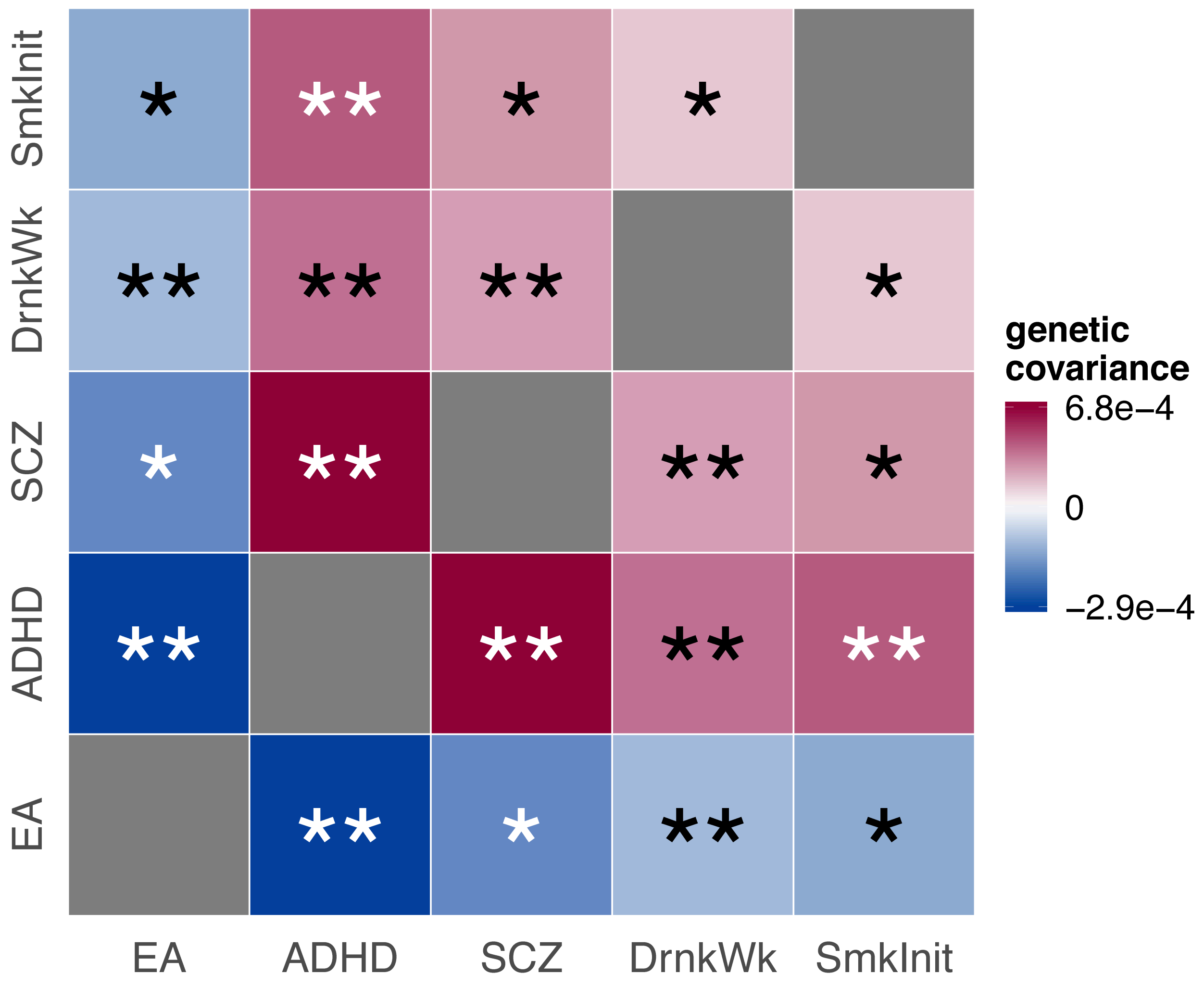

Supplementary Figure 22. LocusZoom plots for AN, BD, MDD, CP, SCZ, SmkInit, and NSM at the *NCAM1* locus. AN-SCZ, BD-SmkInit, MDD-SmkInit, NSM-SmkInit, and DrnkWk-SCZ are positively correlated in the highlighted region and AN-CP, CP-SCZ, and CP-SmkInit are negatively correlated in the highlighted region.

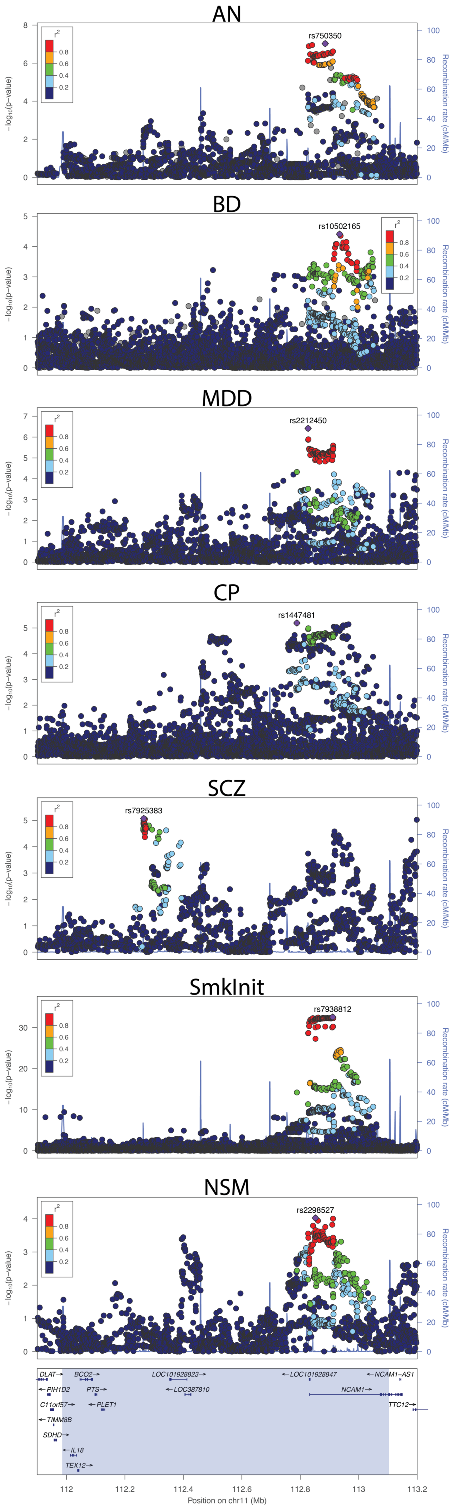

Supplementary Figure 23. Estimates of local genetic covariance among CP, AN, MDD, NSM, BD, SmkInit, and SCZ at the *NCAM1* locus. Single asterisks highlight significant genetic covariances after Bonferroni correction for 435 pairs (among the 30 traits). Double asterisks highlight significant genetic covariances after Bonferroni correction for all 1,006,072 regions in 435 trait pairs.

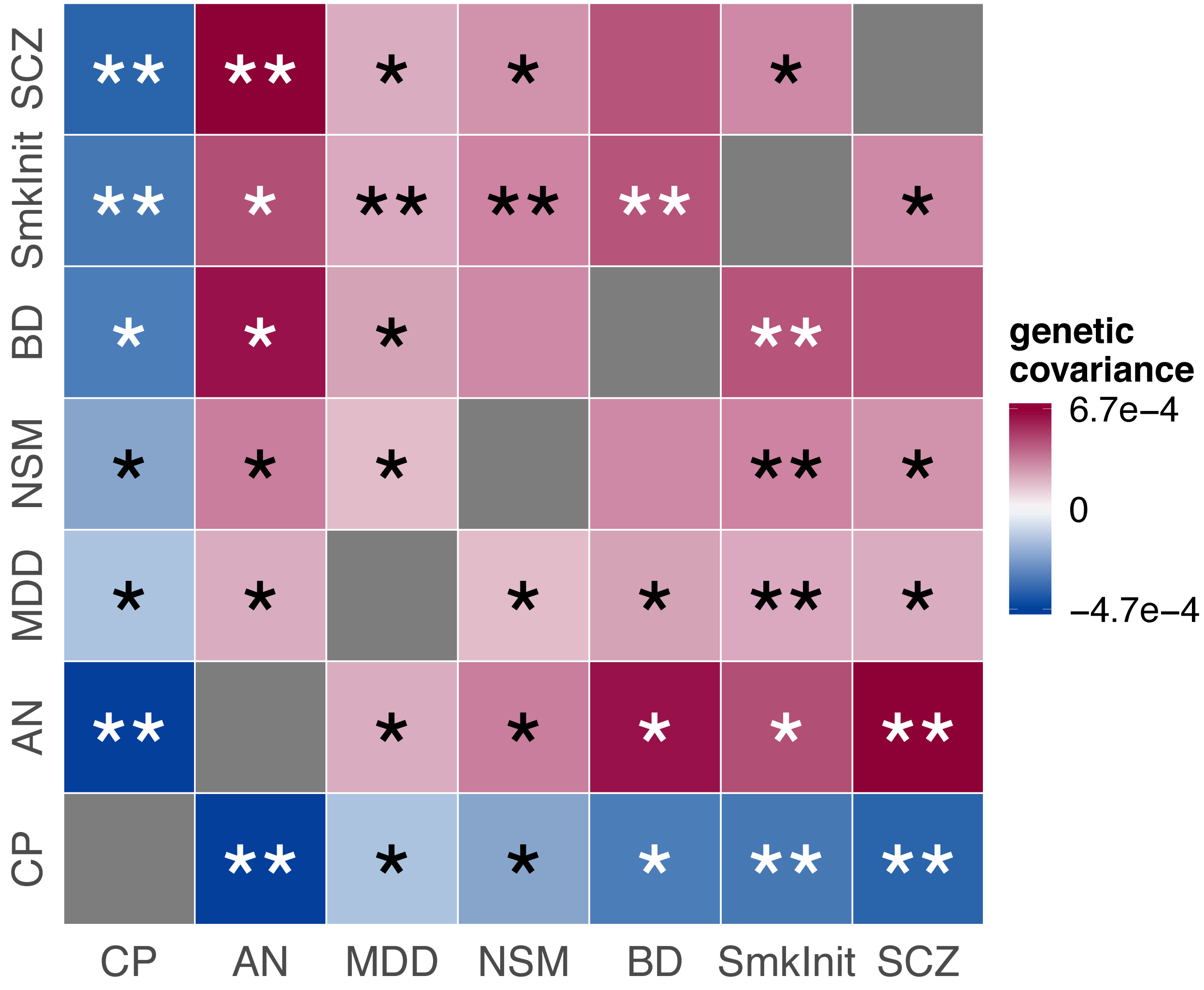

Supplementary Figure 24. Local genetic covariance at the *SPI1* locus among AD, DrnkWk and NSM. (A) replications of local genetic covariances estimates using UKBB AD-proxy summary data. The horizontal axis and the vertical axis represent local covariance estimates based on IGAP2019 AD and AD-proxy summary data, respectively. 95% confidence intervals are shown by the gray dashed lines. Panels B-C demonstrate overlap of genes identified by S-PrediXcan for AD, DrnkWk and NSM in **(B)** macrophages and **(C)** monocytes, respectively. **(D)** Genes and local Manhattan plots for AD, DrnkWk, and NSM at the *SPI1* loci where local genetic covariance was identified and successfully replicated among these traits.

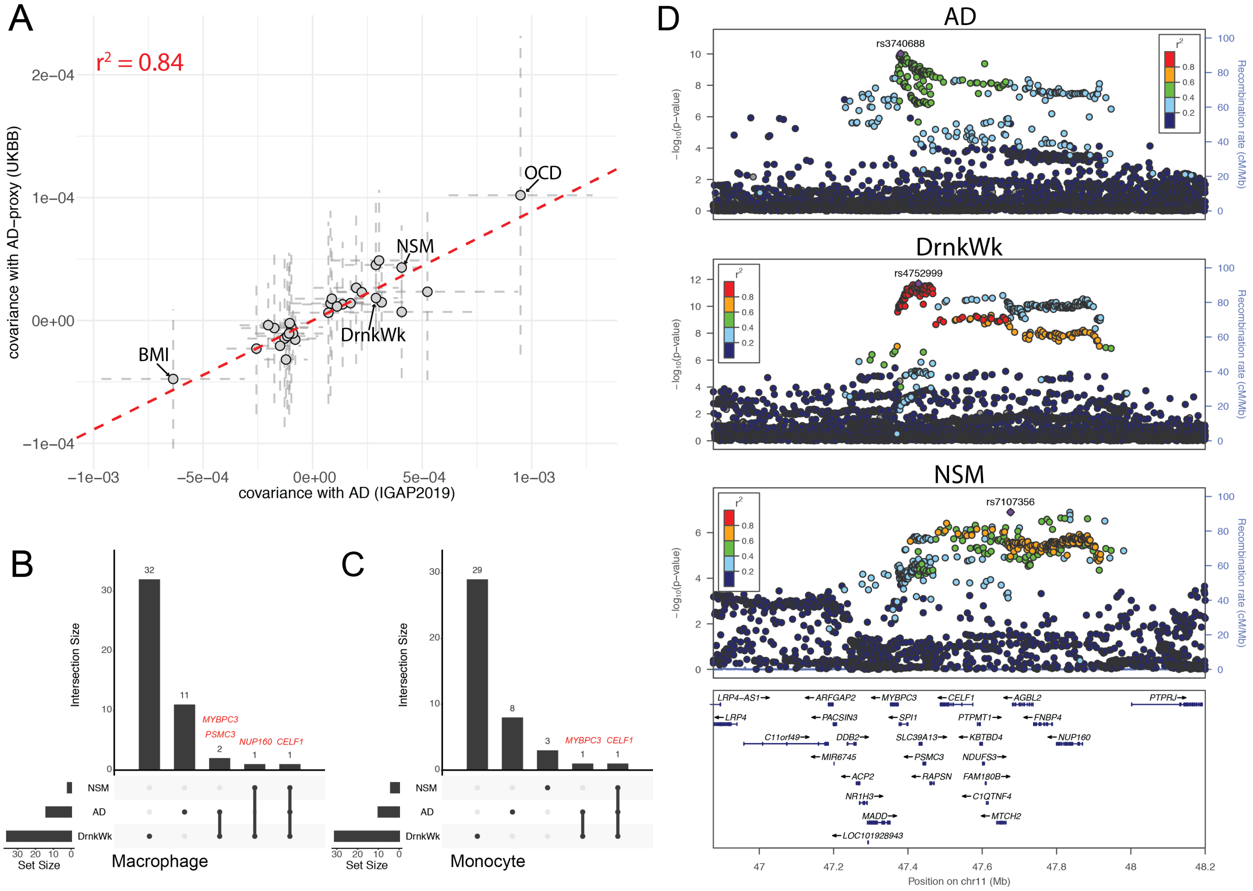

Supplementary Figure 25. Workflow of ASD-CP local genetic covariance analysis.

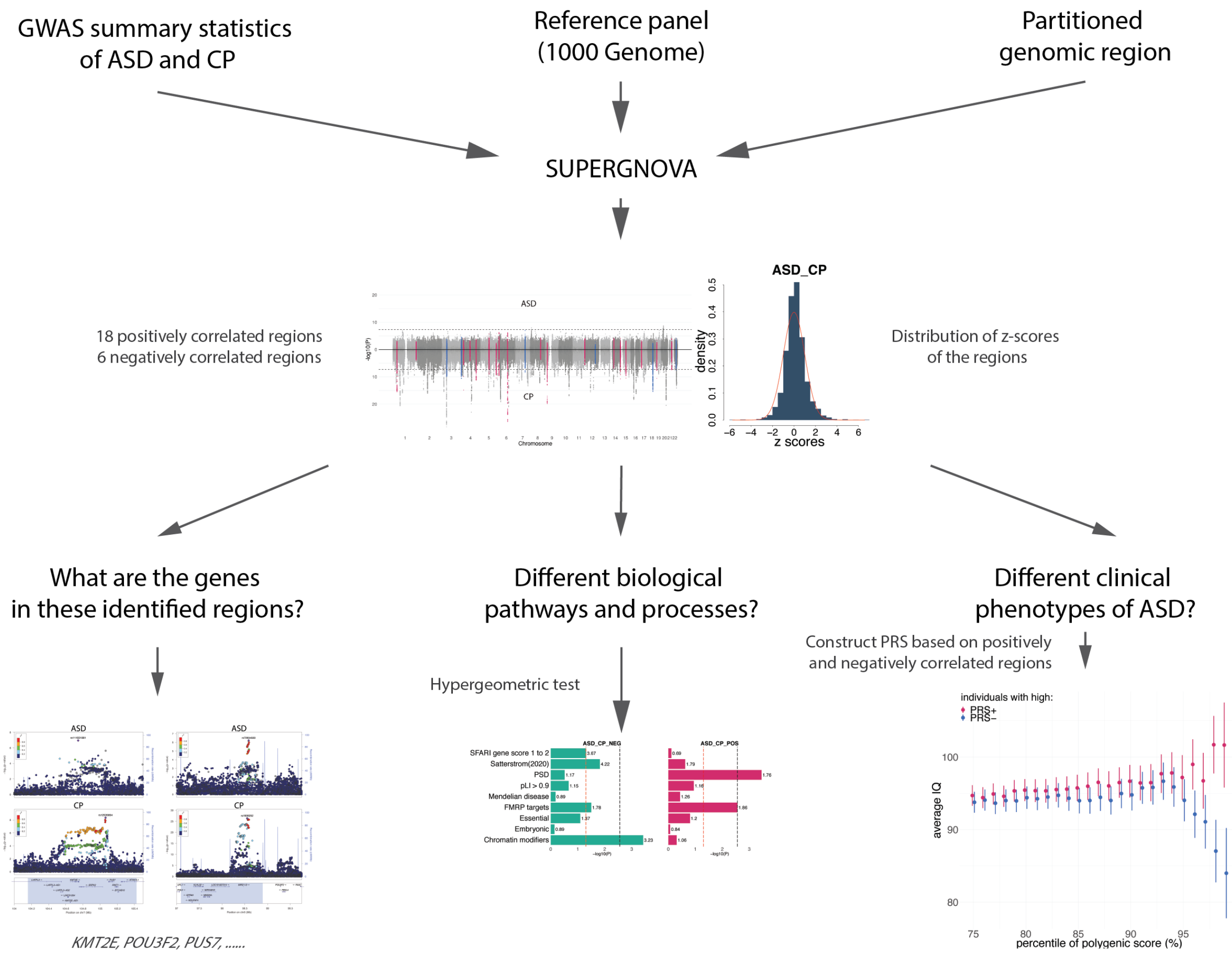

Supplementary Figure 26. Mirrored Manhattan plots for ASD-CP, ADHD-ASD, and ADHD-CP. Regions highlighted in red and blue are positively and negatively correlated, respectively, at an FDR cutoff of 0.1.

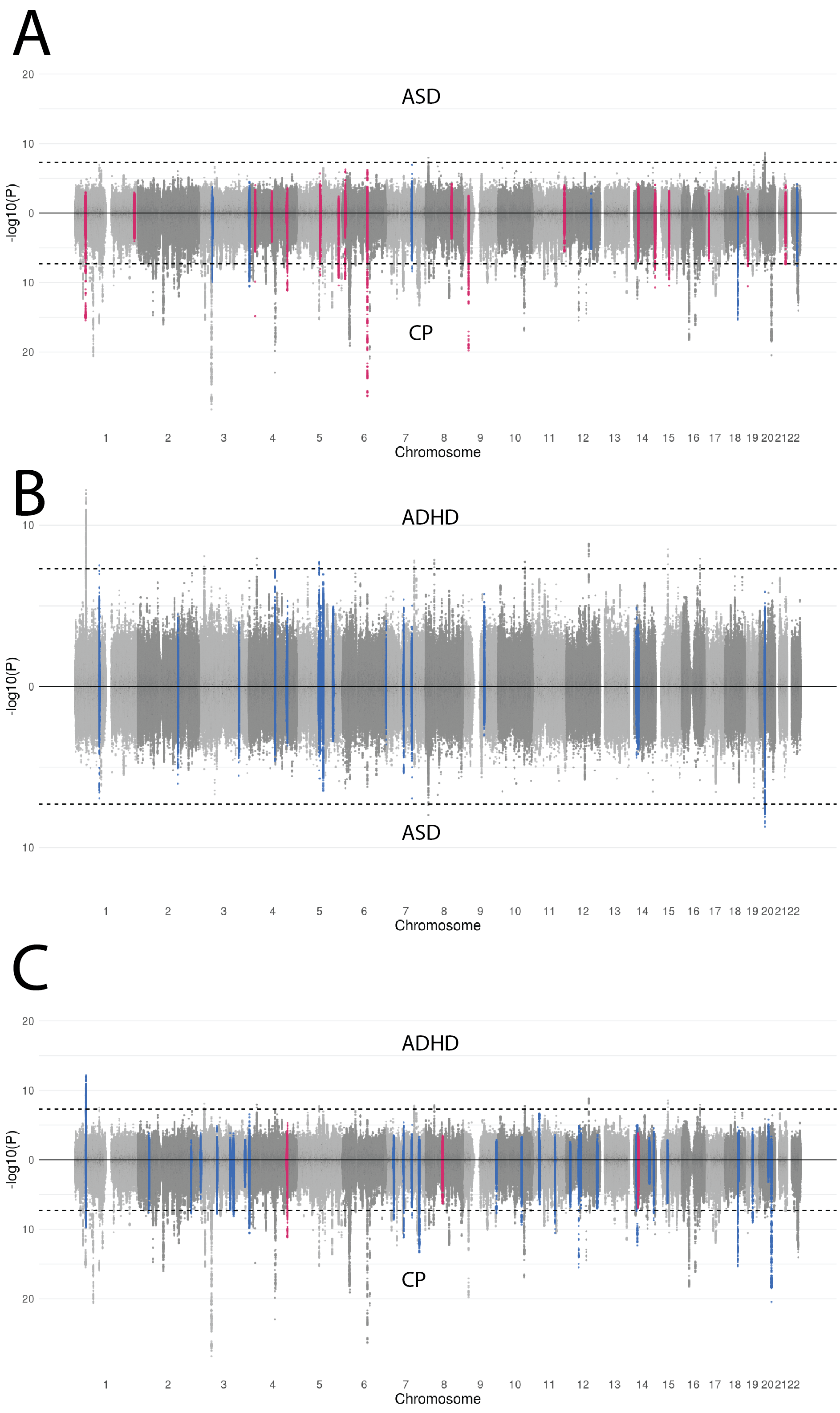

Supplementary Figure 27. LocusZoom plots for ADHD, ASD, and CP at chromosome 4: 150.5M-153.3M. ADHD, ASD, and CP are mutually positively corelated in the highlighted region.

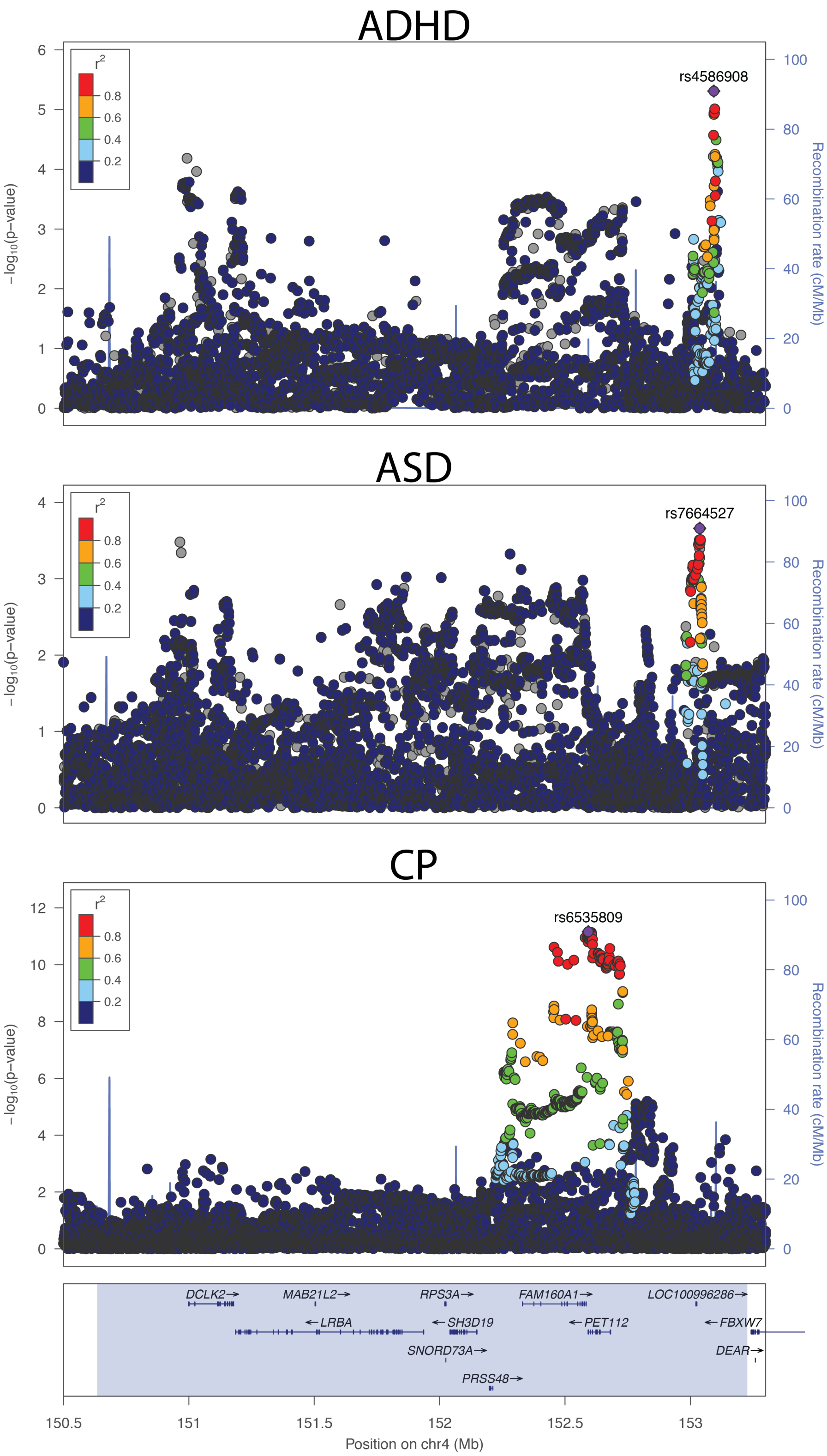

Supplementary Figure 28. LocusZoom plots for ADHD, ASD, and CP at chromosome 14: 36.5M-38.5M. ADHD, ASD, and CP are identified as mutually positively correlated at the highlighted region.

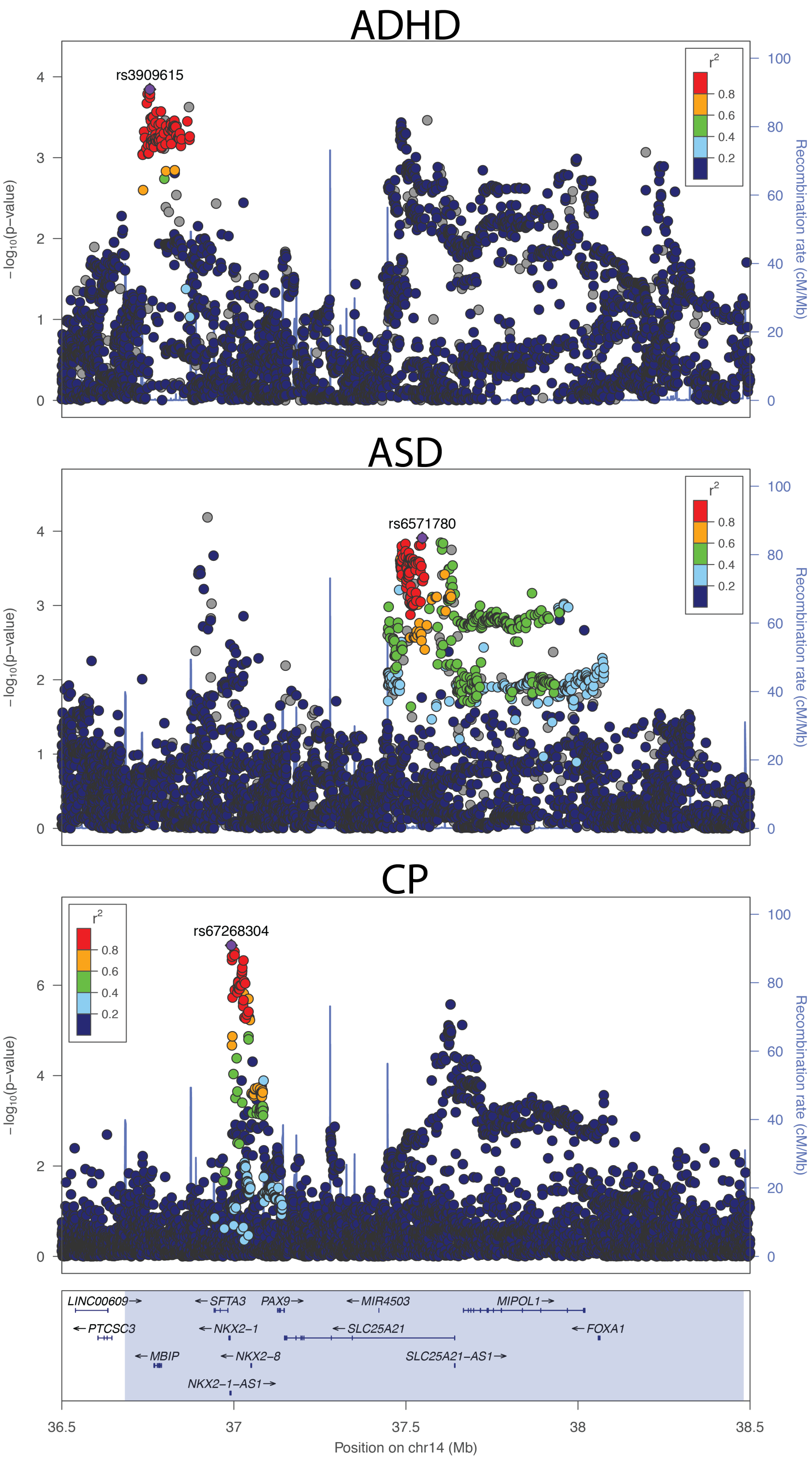

Supplementary Figure 29. LocusZoom plots for ADHD and SCZ at the *KMT2E* locus. The SNP in 1000 Genomes 2014 release with the lowest p value in GWAS was chosen to be the index SNP. Besides the correlations among ADHD, ASD and CP, SCZ was also identified to be negatively correlated with CP at the highlighted region.

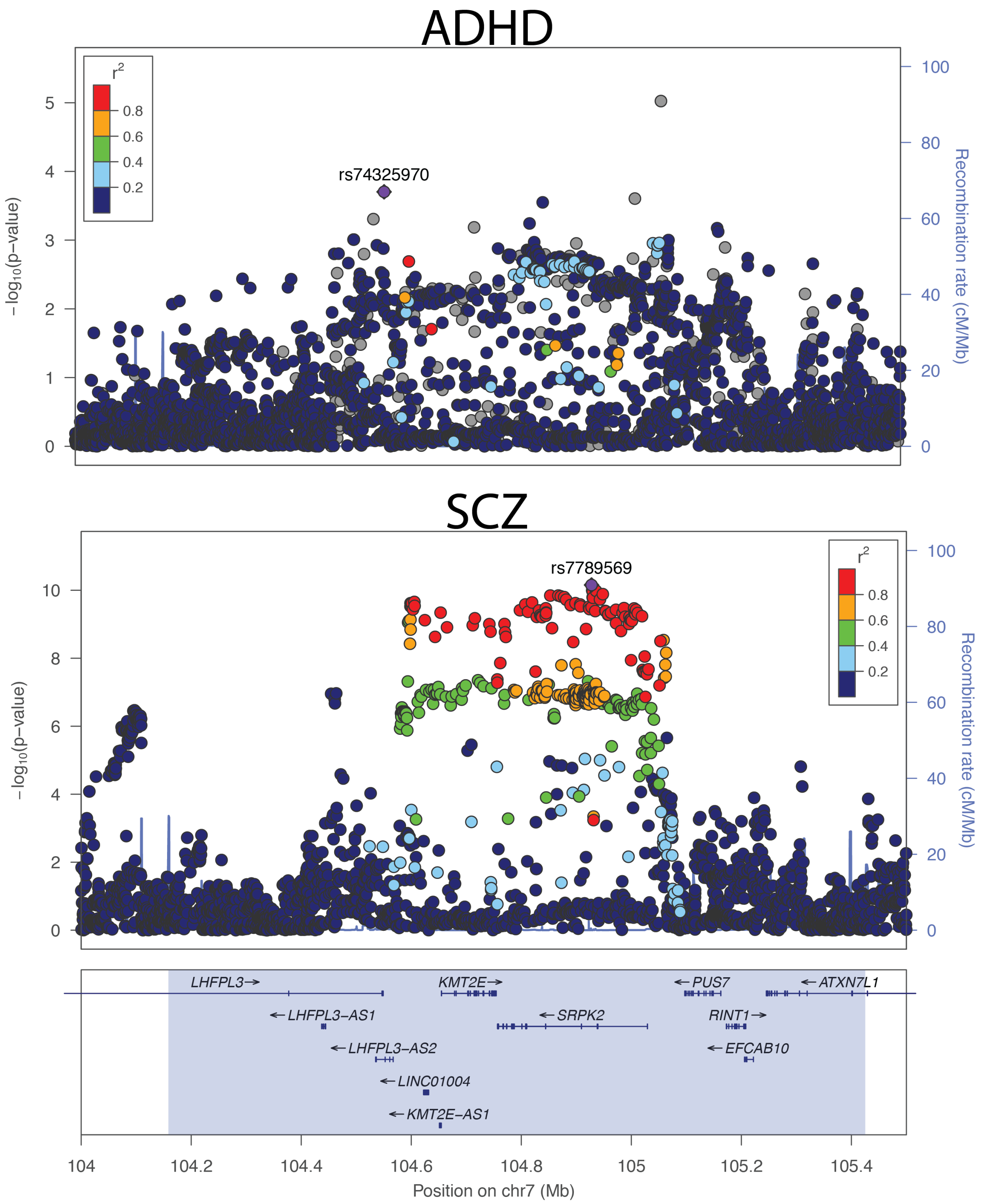

Supplementary Figure 30. LocusZoom plots for AN, BD, DrnkWk, EA, and SmkInit at the *POU3F2* locus. SNP in 2014 1000 Genome reference with the least p value for the association is chosen to be the index SNP. Positive correlations are identified among these neuropsychiatric phenotypes at the highlighted region.

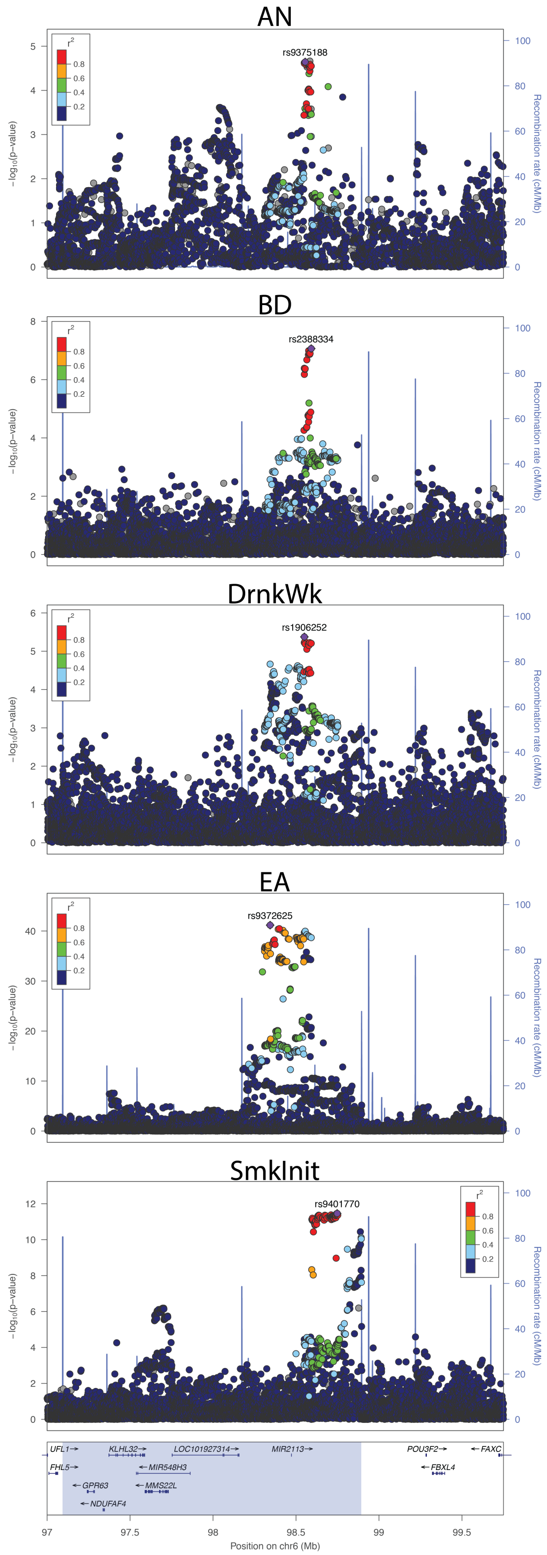

Supplementary Figure 31. Estimates of local genetic covariance among the 7 correlated neuropsychiatric phenotypes at the *POU3F2* locus. Asterisks highlight significant genetic covariances after Bonferroni correction for 435 trait pairs.

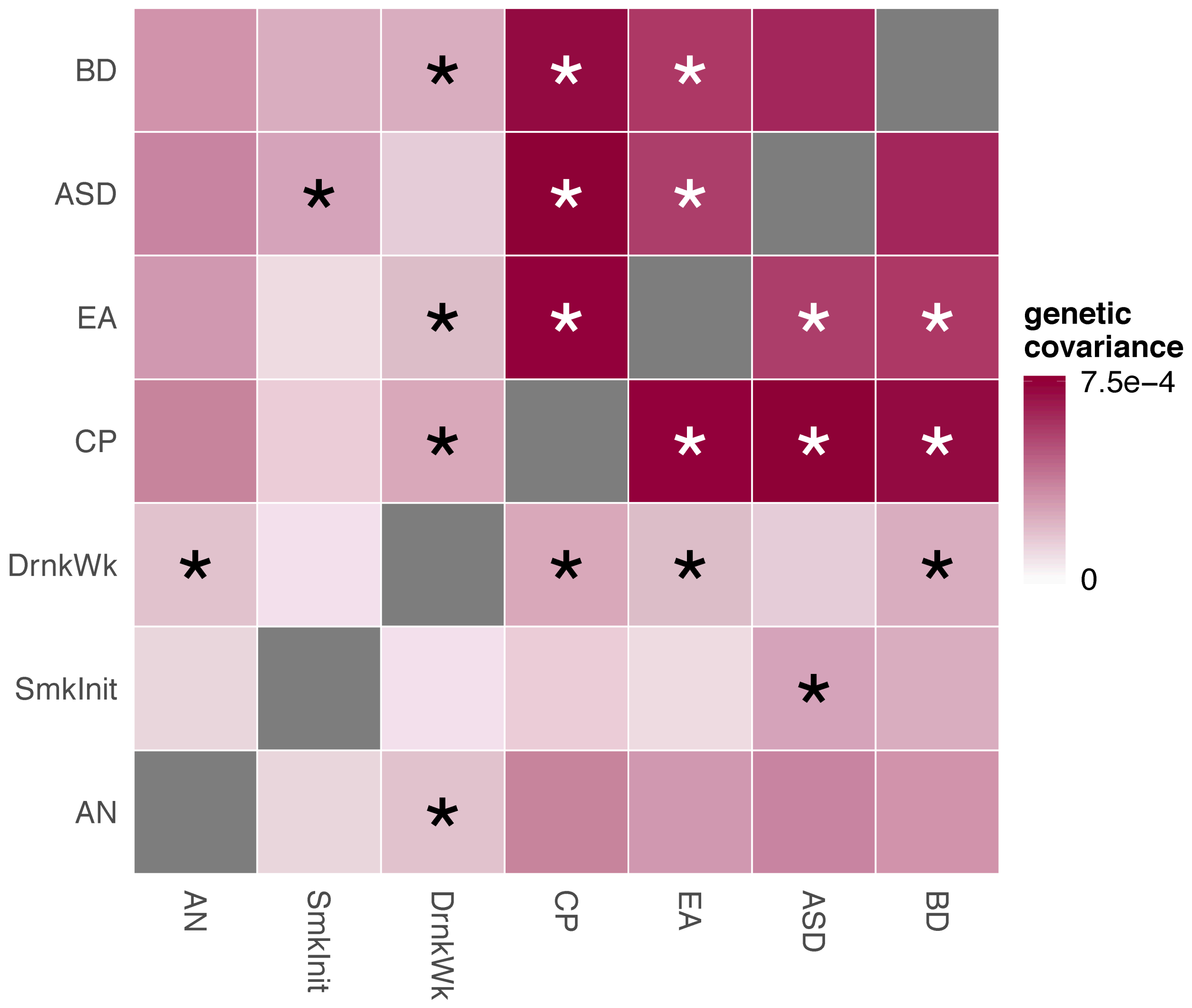

Supplementary Figure 32. Enrichment for genetic associations with other complex traits in regions with opposite correlations between ASD and CP. Odds ratio values are labeled next to each bar. The red dashed lines mark the p-value cutoff of 0.05 and the black dashed lines denote the p-value thresholds after Bonferroni correction (p=7.5e-5).

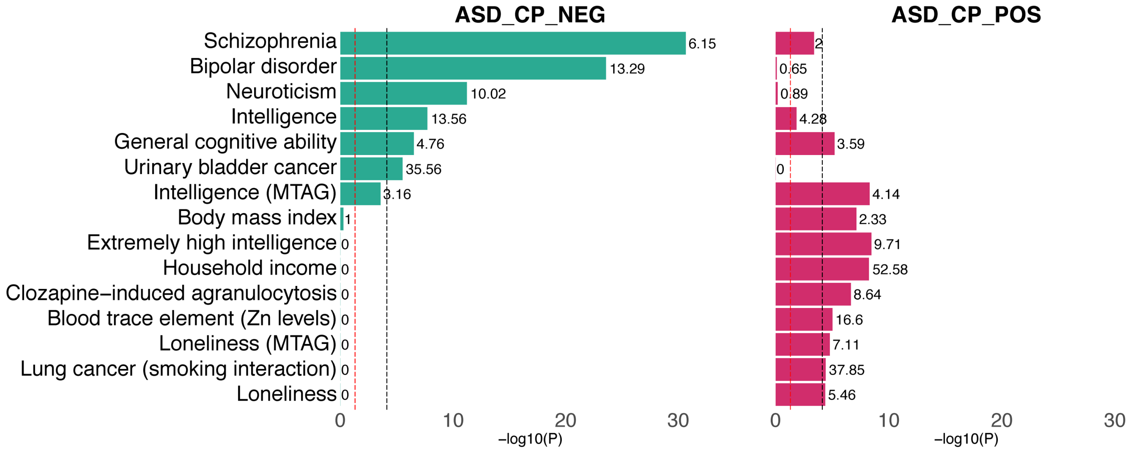

Supplementary Figure 33. Stratified genetic covariance of 28 complex traits with ASD and CP in regions identified with significant ASD-CP covariance. Red intervals denote genetic covariance in regions with a positive ASD-CP covariance. Blue intervals denote genetic covariance in regions with a negative ASD-CP covariance. Triangle dots denote genetic covariance between ASD and other traits. Circle dots denote genetic covariance between CP and other traits. Traits with a suggestive genetic covariance with either ASD or CP ($p<0.05$) are shown. Asterisks highlight significant covariances after Bonferroni correction. The interval length represents standard errors.

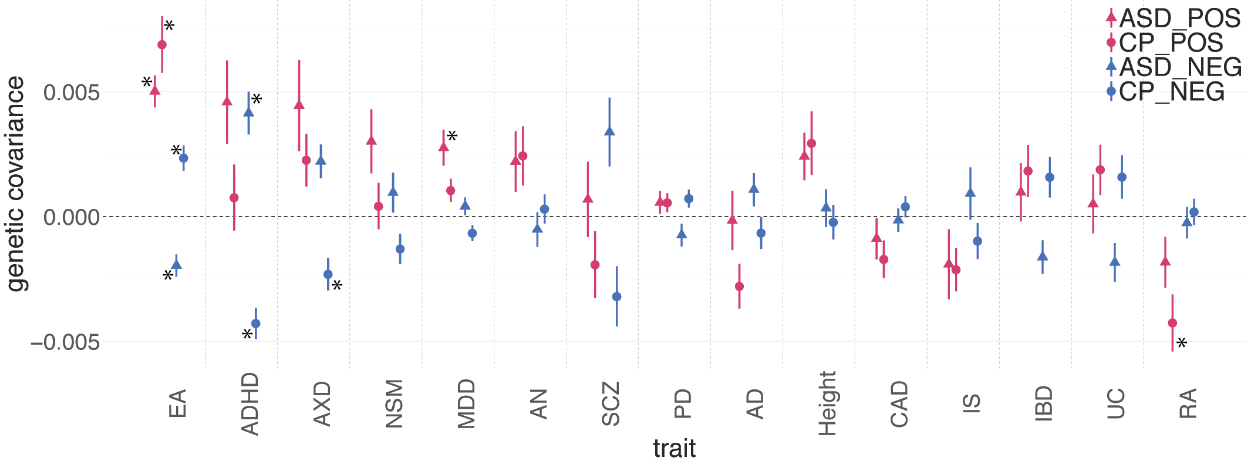

Supplementary Figure 34. Cumulative gene expression rate in fetal brain of ASD-CP positively and negatively correlated genes and other genes across developmental stages. Standard error of gene expression rate estimation for ASD-CP positively and negatively correlated genes and other genes is shown.

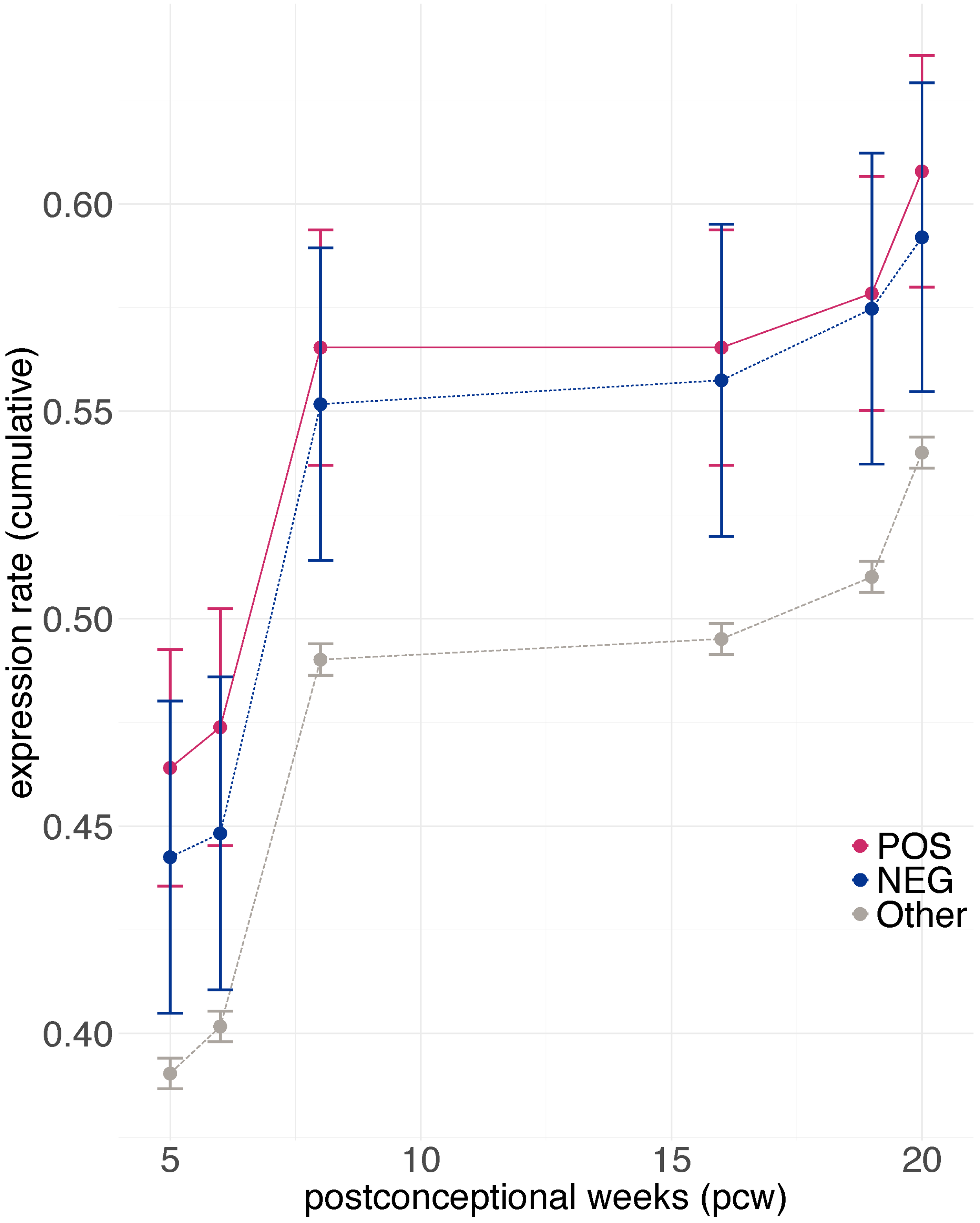

Supplementary Figure 35. Gene expression in brain across developmental stages. Means of log-transformed RPKM and standard error are shown. The black dashed line represents neonatal time. The red line, blue line and gray line represents expression levels of ASD-CP positively correlated, negatively correlated and other background genes, respectively.

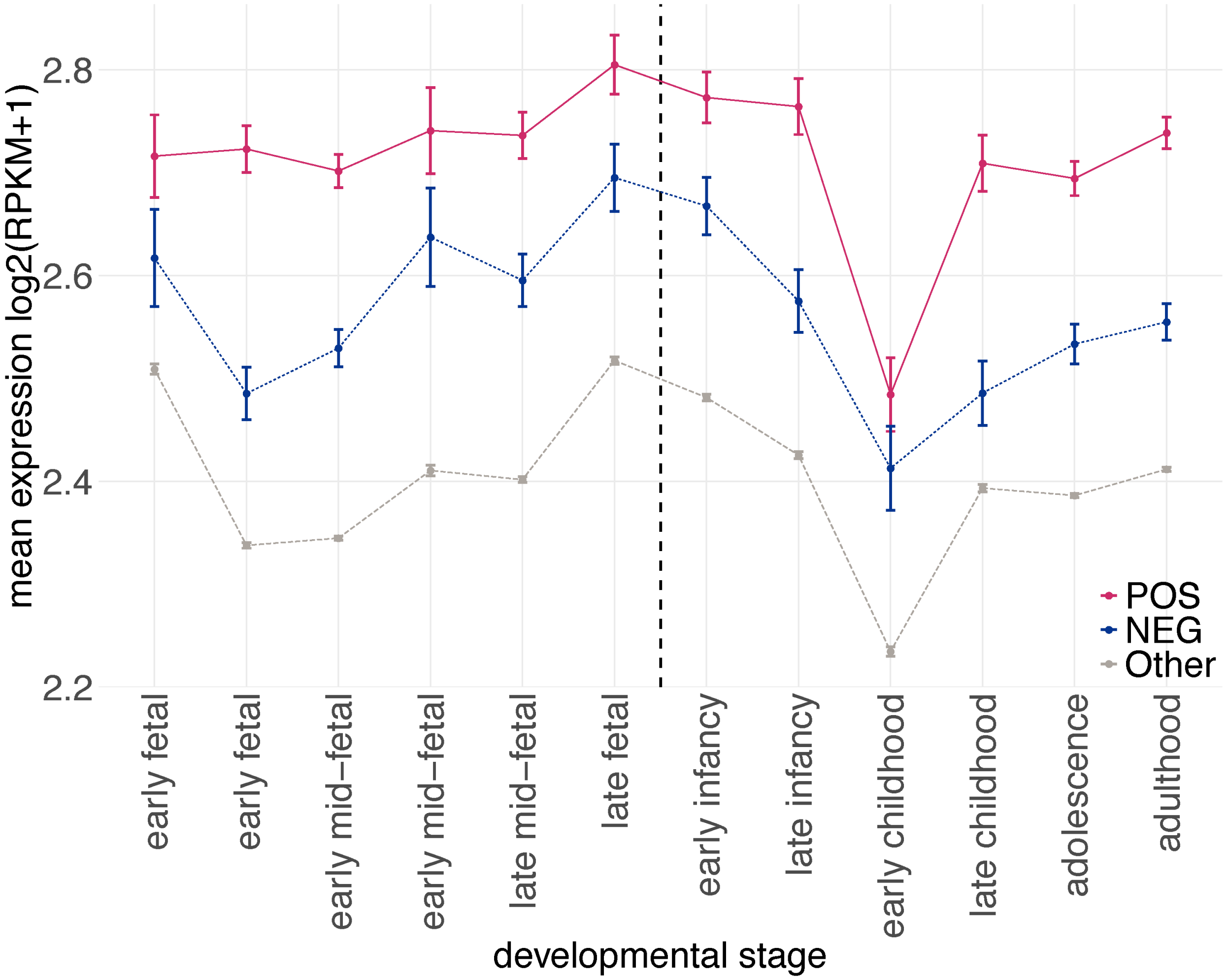

Supplementary Figure 36. Normality of PRSs. We normalized PRS+ and PRS- of ASD probands with sample mean and standard deviation. In panel A-B, we used qq-plot to compare the distributions of normalized **(A)** PRS+ and **(B)** PRS- with standard normal distribution.

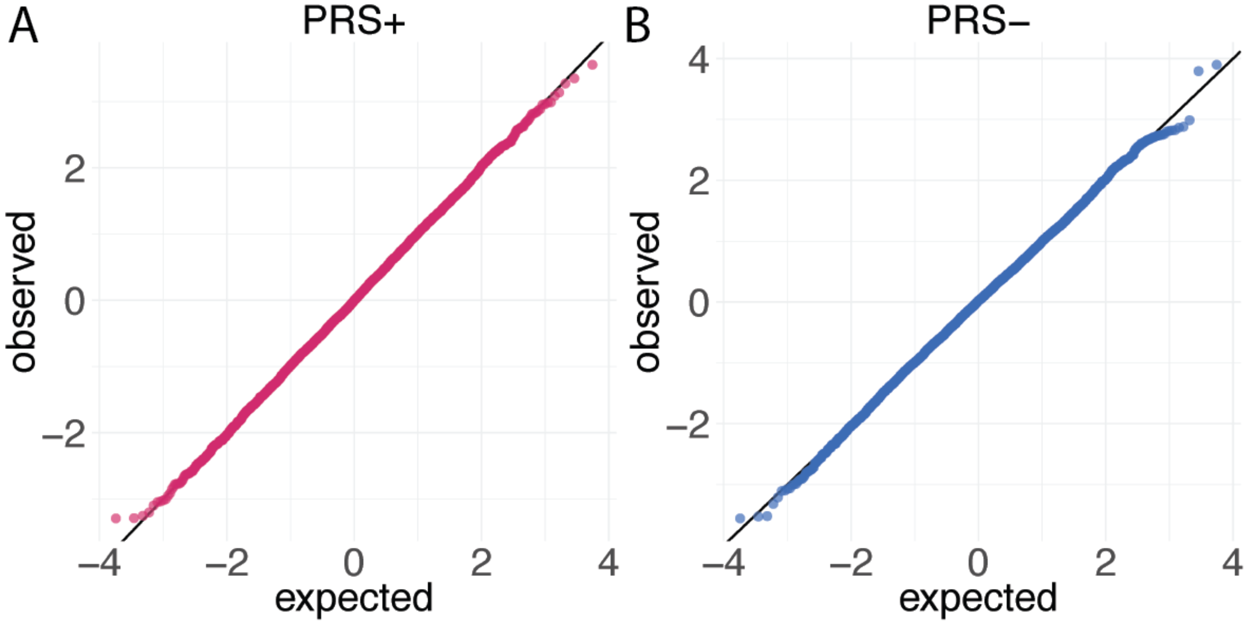

Supplementary Figure 37. Distribution of IQ in ASD probands with extreme PRS+ and PRS- values. The distribution was estimated by the Gaussian kernel approach.

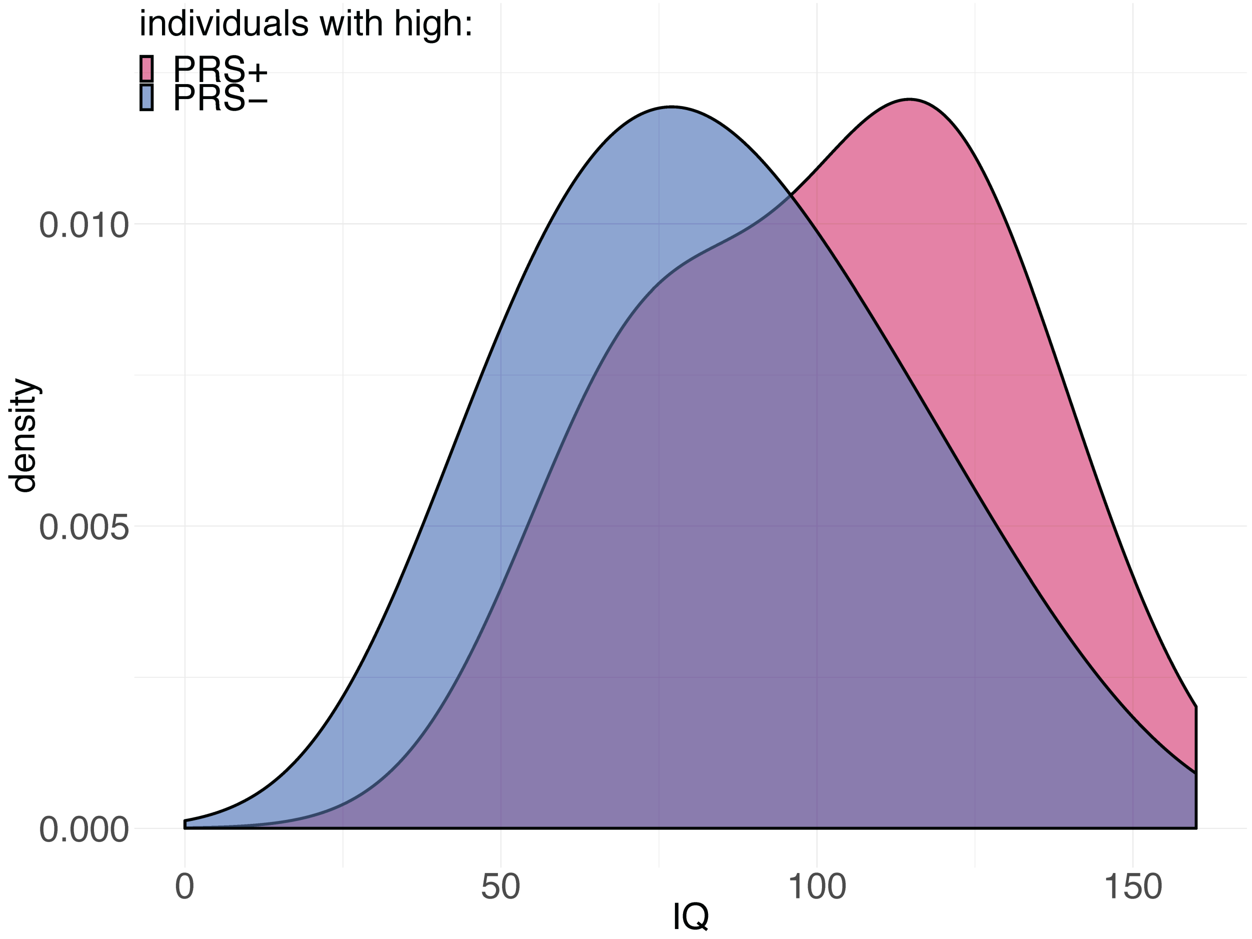

Supplementary Figure 38. Average phenotypic scores of ASD were computed for ASD probands at various PRS percentiles. Intervals denote standard errors for the estimated means. The phenotypic scores are **(D)** SCQ score, **(E)** RBS-R score, and **(F)** DCDQ score.

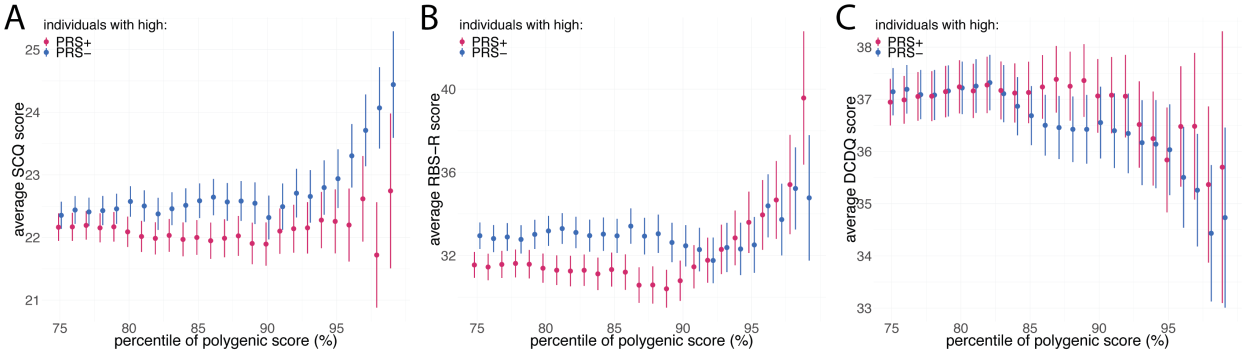

Supplementary Figure 39. Enrichment of ASD subtypes in extreme PRS+ and PRS- groups. Each bar indicates the fold enrichment of a subtype in ASD probands with extreme PRS. The p values of enrichment are annotated to the top of each bar. The dashed line represents enrichment=1. Autism spectrum disorder represents Rett disorder and childhood disintegrative disorder. PPD-NOS represents pervasive developmental disorder – not otherwise specified.

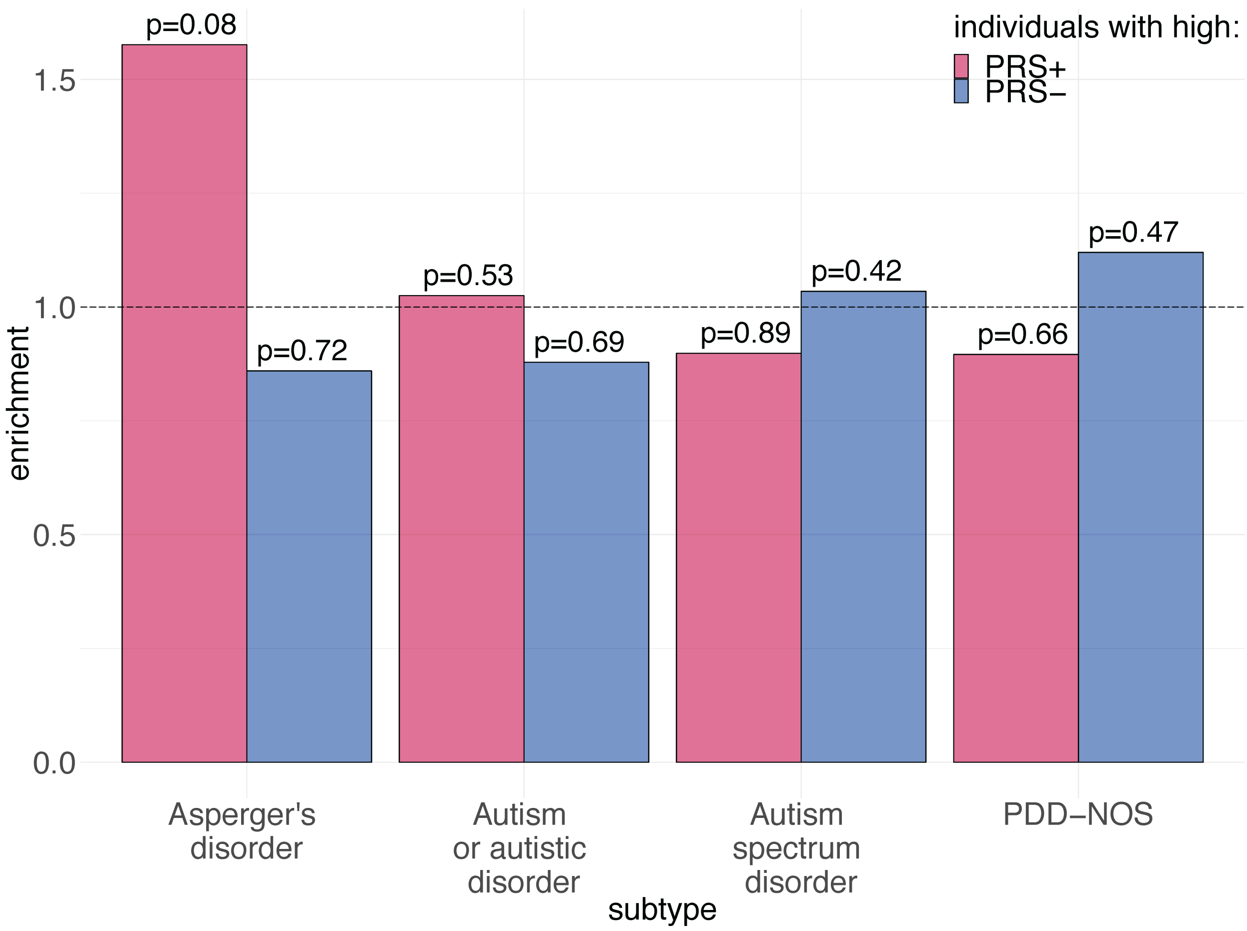

Supplementary Figure 40. Theoretical variance and empirical variance of estimates of local genetic covariance. The x-axis is the cutoff for eigenvalues included in the estimation of local genetic covariance. Theoretical variance decreases with the increase of K_i and empirical variance increase rapidly when the cutoff is small enough. We used the data from one example of simulation to plot this figure.

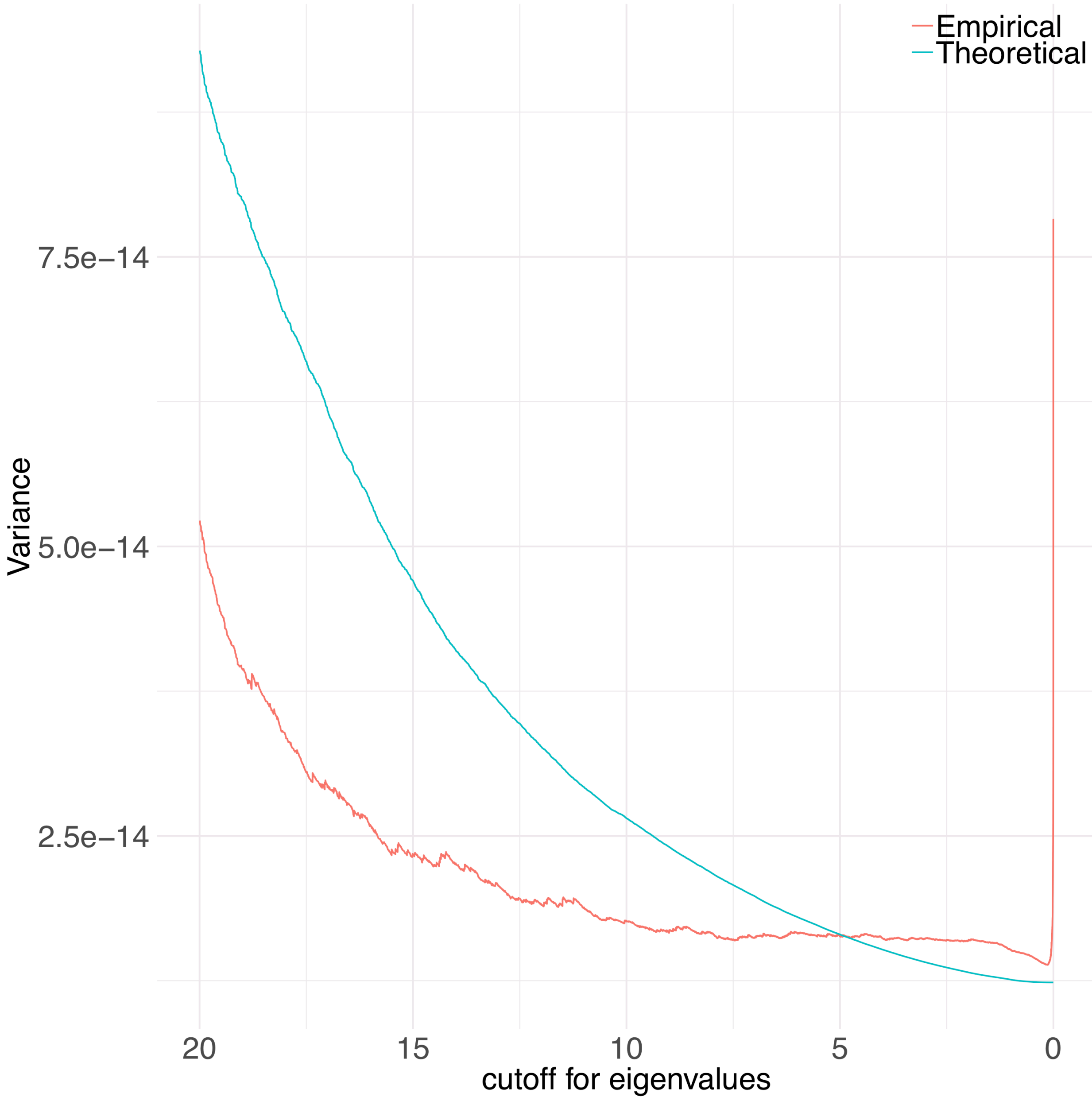

Supplement: Supplementary file 2 — Additional file 2. Supplementary figures 1-40. [file 13059_2021_2478_MOESM2_ESM.pdf]
