# Supplementary material for: SUPERGNOVA: local genetic correlation analysis reveals heterogeneous etiologic sharing of complex traits
Source: Genome Biol. 2021 Sep 7;22:262. doi: 10.1186/s13059-021-02478-w (PMC8422619; doi:10.1186/s13059-021-02478-w)
Supplement: Supplementary file 4 — Additional file 4. Review history. [file 13059_2021_2478_MOESM4_ESM.docx]

**1^st^ round**

**Reviewer 1**

Recent years have witnessed an increasing and useful application of genetic correlations, but dominantly only of global genetic correlations. This paper considers estimation, inference and application of local genetic correlations, a much less discussed topic. A unified statistical framework is proposed, covering existing and popular methods such as LDSC and GNOVA. The proposed new method, called SUPERGNOVA, was shown to be much more powerful than LDSC and slightly better than GNOVA for global genetic correlations, and much more powerful than rho-HESS for local genetic correlations through simulations (e.g. Fig 2). Real data applications, in particular, to paradoxical genetic correlation between autism spectrum disorder and cognitive performance, demonstrate substantial biological insights that can be gained by using the proposed method. To me, the proposed method is well motivated, technically solid and novel, and seems to perform very well, thus will be useful in practice. The paper was well written.

I only have a few MINOR questions for clarification.

1. If I understand correctly, SUPERGNOVA applies WLS to the products of the pairs of the (decorrelated) z-scores in a local region, hence it looks similar to LDSC (in estimating a global genetic correlation). Would you please clarify what are their main differences in terms statistical modeling and estimation (putting aside their difference in estimating local versus global genetic correlations)?

With the same reasoning, it seems to me that SUPERGNOVA is quite different from GNOVA (i.e. Regression versus MoM), which makes it hard to understand intuitively why they had similar performance in Fig 2 and the statement "When the per SNP heritability is small, SUPERGNOVA is equivalent to GNOVA (Supplementary Note) which is a method that has been proved to achieve theoretical optimality compared to LDSC". Some explanation would help.

2. I understand that estimating local genetic correlations is more challenging due to correlated (and fewer) SNPs in a region, but why "most methods developed for global genetic correlation cannot be directly applied to estimate local correlations"? For example, why cannot LDSC (or its corresponding estimation method) be directly applied to only a local region to estimate the local genetic correlation? If possible and not too much trouble, it would be interesting to see how LDSC would perform for local genetic correlations in the bottom of Fig 2.

Relatedly, how does SUPERGNOVA estimate a global genetic correlation, by applying the method to the whole genome or by aggregating its estimated local genetic correlations? I tried but did not find an explicit formula or discussion in the main test.

3. Sample overlapping is listed as one special challenge in estimating local genetic correlations: "In addition, ubiquitous sample overlap across GWASs introduces technical correlations among association statistics from different studies, which further complicates the estimation of genetic correlation, especially in local regions." But isn't this common to BOTH global and local genetic correlation estimation? For example, as in your proposed method, you can apply LDSC to the whole genome to estimate the non-genetic correlation due to sample overlapping once and only once, regardless what local regions are being considered. Is this right? Some comments would be helpful.

4. It would help to know why rho-HESS was much less powerful than SUPERGNOVA, e.g. in Fig 2. The estimation method of rho-HESS seems to be straightforward in using the estimated marginal effect sizes of the SNPs (similar to z-scores) and a reference LD matrix (that is also used in SUPERGNOVA). It is noted that rho-Hess is based on assuming fixed effects of the SNPs, in contrast to SUPERGNOVA's random effects (as well as of LDSC and GNOVA). Is their performance difference due to the different estimation methods or different models? A comment on the choice of the model and which one is preferred would be helpful, given that there are other models or definitions for genetic correlation (e.g. in the listed reference [15]) and that this important issue does not seem to be much discussed or debated in the literature.

**Reviewer 2**

The authors report on a novel method for detecting local genetic correlation: SUPERGNOVA and conduct simulations and an empirical application to investigate the performance of this method. Several other methods allow the estimation of local genetic correlation, including p-HESS, LOGODetect and LAVA. The authors conduct a systematic comparison with p-HESS. The advantages over global genetic correlation analysis include: Pinpointing of influential regions of genome, regions of positive and negative correlation, and increased mechanistic insight from local correlations.

This is a generally well written manuscript and I have the following suggestions for improvement:

Major comments:

1)	The method relies on LD blocks, would be useful to have approach that directly estimates LD and integrates into algorithm in dynamic manner (LD not consistent across the genome)

2)	Line 168ish: With respect to simulation studies, it would be interesting to see diversity of genetic architectures (number of causal SNPs) in simulations, differences in LD patterns, etc. Also, a brief description of the simulation study in the results section, would help the reader to interpret results without going back and forth to the methods section

3)	Line 100: The authors refer to the positive genetic correlation between ASD and cognitive ability as a paradox, but I believe there are epidemiological studies also reporting a positive phenotypic association. Can the authors provide more background information explaining why this positive genetic correlation is a paradox?

4)	Line 135: Figure 1 is very helpful but can the authors explain how sample overlap is distinguished from LD when decorrelating Z-statistics. Can the correction for overlap in Sample size be added to Figure 1?

5)	The primary simulation analysis are conducted using WTCCC data. Does the relatively small sample size limit the ability to simulate realistic GWAS scenario's?

6)	The authors conduct PRS analyses to further explore the influence of ASD-CP positive genetic correlation regions and ASD-CP negative genetic correlation regions. Although I find the question in itself interesting, the results of this specific section seem weak. The main problem is that the authors do not control the type-I error rate. For some analyses they use PRS percentiles, for other analyses they distinguish between extreme and non-extreme PRS scores. It is not always clear what the comparison group is. Some of the discussed findings are not even significant at P<0.05 and combined with a failure to control the family-wise type I error rate, I was not convinced that these findings are robust. The authors should revise their approach, correct for multiple testing and better explain why they apply different PRS thresholds in the different analyses.

7)	Sentence 476: What is the test statistic of the overall test? What happens at percentiles below 75. Can this be added to the figure 6A?

8)	Line 484: please clarify, Figure 6C does not help and I did not understand this sentence.

Minor comments:

There are some sentences that are difficult to read. E.g., lines 443-445; line 484

**Authors’ response**

**1. If I understand correctly, SUPERGNOVA applies WLS to the products of the pairs of the (decorrelated) z-scores in a local region, hence it looks similar to LDSC (in estimating a global genetic correlation). Would you please clarify what are their main differences in terms statistical modeling and estimation (putting aside their difference in estimating local versus global genetic correlations)?**

Thank you for your comment. As the reviewer correctly pointed out, SUPERGNOVA transforms z scores from each input GWAS to decorrelate the marginal association statistics. LDSC does not apply this transformation. With this transformation, SUPERGNOVA can fully utilize information from the covariance of z scores while LDSC only models the diagonal terms in the covariance matrix. We provide more detailed explanations below. In the **Methods** section and the **Supplementary Note** of our manuscript, we have shown that the covariance of association statistics from two GWASs (i.e., 𝑧" and 𝑧#) is

𝐶𝑜𝑣(𝑧", 𝑧#) = √𝑛"𝑛#𝜌

𝑚

𝑉# +

𝑛2𝜌3

√𝑛"𝑛#

𝑉. (1)

Here, 𝜌 quantifies genetic covariance and is the parameter of interest; 𝑉 denotes the LD matrix; 𝑚 is the number of SNPs; 𝑛", 𝑛#, and 𝑛2 denote the sample size in study-1, study-2, and the shared sample size, respectively; and 𝜌3 is defined as the sum of genetic covariance and non-genetic effects covariance. This formula is the backbone of most methods for global genetic covariance estimation.

If we denote the eigen decomposition of 𝑉 as 𝑉 = 𝑈Σ𝑈8, then we can show that

𝐶𝑜(𝑈8𝑧", 𝑈8𝑧#) = √9:9;<

= Σ# + 9><?

√9:9;

Σ. Further, if we denote 𝑧̃" = 𝑈8𝑧" and 𝑧̃# = 𝑈8𝑧#, the elements in 𝑧̃" and 𝑧̃# are independent and we can apply weighted least squares on the element-wise product of 𝑧̃" and 𝑧̃# to estimate genetic covariance. This is important since we can easily obtain a closed form solution for the standard error of 𝜌A given that element-wise products 𝑧̃"B𝑧̃#B are independent.

There are several major advantages of this approach compared to LDSC. First, LDSC does not decorrelate the z scores using eigenvectors of 𝑉. Instead, it estimates genetic covariance through minimizing the distance between element-wide products 𝑧"B𝑧#B and the diagonal terms of 𝐶𝑜(𝑧", 𝑧#) in formula (1) above, i.e., 𝐸D𝑧"B𝑧#BE = √9:9;< = 𝑙B + 9><? √9:9; , where 𝑙B is the LD score for SNP 𝑗. This ignores the numerous non-diagonal terms in 𝐶𝑜(𝑧", 𝑧#) and may result in substantial information loss. In our approach, such information is preserved in transformations 𝑈8𝑧" and 𝑈8𝑧#. In addition, the element-wise products 𝑧"B𝑧#B used in LDSC are not statistically independent. To address this issue (i.e., data points are not i.i.d.), LDSC implements a block-jackknife approach to estimating the standard error of 𝜌A. This procedure is more computationally intensive than the close-form solution in SUPERGNOVA. More importantly, it cannot be used in local genetic covariance estimation due to substantial LD in local genomic regions and likely violation of the block-wise independence assumption in block-jackknife. In summary, the approach implemented in SUPERGNOVA leverages full information in the covariance matrix 𝐶𝑜𝑣(𝑧", 𝑧#) which benefits both global and local genetic covariance analyses, and provides close-form estimates on standard errors which is computationally more efficient and particularly important for local covariance applications. Since the standard error estimation is also a central topic in the response to comment #3 below, we will provide more detailed explanations about estimating standard error in our response to the later comment.

Related clarifications have been added to the **Overview of SUPERGNOVA analytical framework** section of our revised manuscript.

**2. With the same reasoning, it seems to me that SUPERGNOVA is quite different from GNOVA (i.e. Regression versus MoM), which makes it hard to understand intuitively why they had similar performance in Fig 2 and the statement "When the per SNP heritability is small, SUPERGNOVA is equivalent to GNOVA (Supplementary Note) which is a method that has been proved to achieve theoretical optimality compared to LDSC". Some explanation would help.**

Thank you for your comment. In the **Supplementary Note** of our manuscript (**Section 2**), we demonstrated that if per-SNP heritability is small, i.e., (𝑛3ℎ3I # ⁄𝑚I)IB = 𝑜(1) (𝑡 = 1,2), the estimates of SUPERGNOVA are equivalent to the estimates of GNOVA. Here, 𝑛3 is the sample size of study 𝑡, ℎ3I # ⁄𝑚I is the per-SNP heritability in the ith genomic region, and 𝑤IB is the jth eigenvalue of LD matrix in the ith region. In practice, the average value of 𝑤IB for each region is 1, per-SNP heritability ℎ3I # ⁄𝑚I is on the magnitude of 10OP~10OR, and the sample size 𝑛3 is about 10S~10T. Therefore, the value of (𝑛3ℎ3I # ⁄𝑚I)IB is about 10OS~10O#, which can be considered as 𝑜(1).

To make it easier for reviewers to review these details, we show the related derivations in **Supplementary Note** here in the following figure. We also introduce the intuition behind the derivations. For the GNOVA estimator shown in equation (15) in **Supplementary Note**, the sum of LD scores across all SNPs is in the denominator, which is exactly 𝑡(𝑉#). 𝑡(𝑉#) is also the sum of the squared eigenvalues of 𝑉. Therefore, the point estimations of SUPERGNOVA and GNOVA are mostly similar. However, the approaches to estimate standard errors are different in GNOVA and SUPERGNOVA.

We have improved these details in the **Overview of SUPERGNOVA analytical framework** section of the revised manuscript.

**Figure.** Screenshot for the derivations for the consistency of SUPERGNOVA and GNOVA from the

**Supplementary Note**.

**3. I understand that estimating local genetic correlations is more challenging due to correlated (and fewer) SNPs in a region, but why "most methods developed for global genetic correlation cannot be directly applied to estimate local correlations"? For example, why cannot LDSC (or its corresponding estimation method) be directly applied to only a local region to estimate the local genetic correlation? If possible and not too much trouble, it would be interesting to see how LDSC would perform for local genetic correlations in the bottom of Fig 2.**

Thank you for your questions and suggestion. As you correctly pointed out, a major challenge for local genetic covariance estimation is the extensive LD in a genomic region. While the point estimates of LDSC remains valid when directly applied to a local genomic region, the block-jackknife approach in LDSC will fail to estimate the standard error of local genetic covariance. This is because block-jackknife assumes SNPs in different blocks to be independent. When applied to genome-wide summary data, LDSC divides the whole genome into ~200 regions and applies block-jackknife on the 200 blocks. This is a reasonable approach for global genetic correlation estimation because the average size for each block is ~15mb. The correlation of SNPs between different blocks is relatively small given the large size of the blocks. However, when applied to local genomic regions, we cannot divide the region into sufficient blocks while maintaining block independence. Violation of this assumption will lead to underestimated standard error and inflated type-I error rates. The following figure shows the distribution of the p-values from LDSC when the true local genetic covariance is zero. The simulation setting follows the same procedure in the original submission which uses two independent subgroups of WTCCC samples. We have added the related discussions in the **Simulation** section of the revised manuscript. We also added the description of the related simulation in the **Supplementary Note**. The results have been included as **Supplementary Figure 40** in the revised manuscript.

**Figure (Supplementary Figure 40 in the revised manuscript).** The distribution of LDSC p-values (log transformed) when the true local genetic correlation was set to be zero. LDSC showed severe type-I error inflation when applied to local genetic correlation estimation.

**4. Relatedly, how does SUPERGNOVA estimate a global genetic correlation, by applying the method to the whole genome or by aggregating its estimated local genetic correlations? I tried but did not find an explicit formula or discussion in the main test.**

To estimate global genetic correlation, SUPERGNOVA treats the whole genome as a “big region” and applies the same method for local genetic correlation. Since it is computationally expensive to implement SVD on the global LD matrix, SUPERGNOVA assumes the global LD matrix to be block-wise diagonal. Under this assumption, SVD can be applied separately to each diagonal block of the global LD matrix. Both eigenvalues and eigenvectors of the global LD matrix can be easily derived from the SVD results of the submatrices. Similar to local genetic correlation estimation, the genomic regions corresponding to the block-wise submatrix are determined by LDetect[1]. We have added related details in the **Local genetic covariance estimation** section of our revised manuscript.

**5. Sample overlapping is listed as one special challenge in estimating local genetic correlations: "In addition, ubiquitous sample overlap across GWASs introduces technical correlations among association statistics from different studies, which further complicates the estimation of genetic correlation, especially in local regions." But isn't this common to BOTH global and local genetic correlation estimation? For example, as in your proposed method, you can apply LDSC to the whole genome to estimate the non-genetic correlation due to sample overlapping once and only once, regardless what local regions are being considered. Is this right? Some comments would be helpful.**

Thank you so much for pointing this out. We agree that sample overlap is also a challenge in genome-wide genetic correlation estimation and it may be inaccurate to describe this as a special challenge for local covariance estimation. We have revised the text in the manuscript.

**6. It would help to know why rho-HESS was much less powerful than SUPERGNOVA, e.g. in Fig 2. The estimation method of rho-HESS seems to be straightforward in using the estimated marginal effect sizes of the SNPs (similar to z-scores) and a reference LD matrix (that is also used in SUPERGNOVA). It is noted that rho-Hess is based on assuming fixed effects of the SNPs, in contrast to SUPERGNOVA's random effects (as well as of LDSC and GNOVA). Is their performance difference due to the different estimation methods or different models? A comment on the choice of the model and which one is preferred would be helpful, given that there are other models or definitions for genetic correlation (e.g. in the listed reference [15]) and that this important issue does not seem to be much discussed or debated in the literature.**

Thank you for your comment. First, we note that 𝜌-HESS also requires overlapping sample size 𝑛2 and random error covariance 𝜌V as input while SUPERGNOVA only needs GWAS summary statistics and reference panel. That is, there is more required input for 𝜌-HESS than SUPERGNOVA and the additional input can be difficult to obtain when there is sample overlap between GWASs. Regarding the superior performance of SUPERGNOVA compared to 𝜌-HESS, we believe that the differences in model assumption and parametrization are not the biggest reasons. Instead, improved modeling on the LD structure and more sophisticated parameter selection are two of the key factors that improve the performance of our approach. We provide more detailed explanations below.

To explain why SUPERGNOVA could outperform 𝜌-HESS on local genetic covariance estimation, we start with the intuition of global genetic covariance estimation of SUPERGNOVA. In the **Methods** section and the **Supplementary Note** of our manuscript, we have shown that the covariance of association statistics from two GWASs (i.e., 𝑧" and 𝑧#) is 𝐶𝑜(𝑧", 𝑧#) = √𝑛"𝑛#𝜌 𝑚 𝑉# + 𝑛2𝜌3 √𝑛"𝑛# 𝑉.

Here, 𝜌 quantifies genetic covariance and is the parameter of interest; 𝑉 denotes the LD matrix; 𝑚 is the number of SNPs; 𝑛", 𝑛#, and 𝑛2 denote the sample size in study-1, study-2, and the shared sample size, respectively; and 𝜌3 is defined as the sum of genetic covariance and non-genetic effects covariance. Importantly, as we noted in the response to an earlier comment, LDSC only uses the diagonal terms in this formula to estimate 𝜌, while our method accounts for the entire matrix (i.e., 𝑉 and 𝑉#). Leveraging complete information from the LD reference improves the estimation performance in SUPERGNOVA. Additionally, we have provided theoretical justifications for the improvement in estimation performance in this paper and in an earlier published paper from our group [2]. In the **Supplementary Note** of our manuscript (**Section 2**), we demonstrated that if per-SNP heritability is small, i.e., (𝑛3ℎ3I # ⁄𝑚I)IB = 𝑜(1) (𝑡 = 1,2), the estimates of SUPERGNOVA is consistent with the estimates of GNOVA. Here, 𝑛3 is the sample size of study 𝑡, ℎ3I # ⁄𝑚I is the per SNP heritability of the ith region, and 𝑤IB is the jth eigenvalue of the LD matrix in the ith region. In practice, the average value of 𝑤IB for each region is 1, per SNP heritability ℎ3I # ⁄𝑚I is about 10OP~10OR, and the sample size 𝑛3 is about 10S~10T. Therefore, the value of (𝑛3ℎ3I # ⁄𝑚I)IB is about 10OS~10O#, which can be considered as 𝑜(1). The theoretical results were also backed up by empirical data. **Supplementary Figures 2-4** in our manuscript showed that the performance of SUPERGNOVA estimator is similar to the GNOVA estimator. Two methods provide comparable estimates on global genetic covariance. It has been proven in the Proposition 2 of [2] that the estimator of GNOVA achieves better standard error than LDSC estimator and hence in the estimation of global genetic covariance, SUPERGNOVA and GNOVA both outperform LDSC.

**Figure** (**Supplementary Figure 2-4A** in the revised manuscript). SUPERGNOVA and GNOVA provide similar estimates across different simulation settings. Both methods outperform LDSC.

Now, we explain why SUPERGNOVA outperforms 𝜌-HESS in local genetic covariance estimation. Applied to local genomic regions, SUPERGNOVA generalizes the framework for global genetic covariance estimation by assuming that SNPs in different regions have different values of heritability and genetic covariance. Therefore, the intuition of global and local genetic covariance estimation behind SUPERGNOVA is similar. SUPERGNOVA and 𝜌-HESS adopt different procedures to select the cutoffs in truncated singular value decomposition (SVD). Due to the noise in LD estimation, especially for the smaller eigenvalues, we only use the first 𝐾I eigenvalues to estimate 𝜌I . As described in the **Methods** section of our manuscript, SUPERGNOVA applies an adaptive approach to selecting the value of cutoff 𝐾I , i.e., only the eigenvalues larger than 𝐾I of region 𝑖 are included to estimate local genetic covariance. It decides the value of 𝐾I based on the behavior of two variances defined in the **Methods** section. This procedure is also illustrated in **Supplementary Figure 34**. It tends to select the 𝐾I that optimizes the power while properly controlling the type I error. After 𝐾I is decided, we use weighted least squares to obtain the estimate of genetic covariance which can also improve the estimation. These intuitions were also supported by empirical data. Compared with 𝜌-HESS, SUPERGNOVA shows lower estimation bias and higher statistical power (**Figure 2**; **Supplementary Figures 5-7**).

**Reviewer 2**

**1. The method relies on LD blocks, would be useful to have approach that directly estimates LD and integrates into algorithm in dynamic manner (LD not consistent across the genome)**

Thank you for your suggestion. We used LDetect[1] to partition the genome into local regions. LDetect is a method to identify approximately independent blocks of LD in the human genome. In SUPERGNOVA, the LD information for each genomic region is explicitly modeled during parameter estimation. Therefore, combining LDetect and SUPERGNOVA allows us to dynamically integrate LD into analyses. In our implemented software of SUPERGNOVA, we also allow users to provide their own LD blocks as they see fit. We have added related details into the **Overview of SUPERGNOVA analytical framework** section of the revised manuscript.

**2. Line 168ish: With respect to simulation studies, it would be interesting to see diversity of genetic architectures (number of causal SNPs) in simulations, differences in LD patterns, etc. Also, a brief description of the simulation study in the results section, would help the reader to interpret results without going back and forth to the methods section**

Thank you for your suggestion. We have implemented simulations for diverse genetic architecture in our manuscript, but we did not highlight and describe these settings clearly enough in the main text. In **Section 4** of the **Supplementary Note** of our paper, we had additional simulations to evaluate the robustness of SUPERGNOVA under various effect size distributions, number of causal variants, and LD patterns. Similar to simulations in the main text, we used genotype data from WTCCC to conduct these simulations. Quantitative phenotypes were simulated for two randomly partitioned sample subgroups (N=7,959 in each set). All SNPs in chromosome 2 were set as background SNPs and the largest region in chromosome 2 (chr2: 176,998,822- 180,334,969) was set to be the local region of interest.

We first investigated the robustness of SUPERGNOVA against sparse genetic architecture with fewer causal SNPs. We randomly selected 10% of SNPs inside and outside of the region of interest as the causal SNPs, respectively. The true local genetic covariance values were set to be 0 and 0.005 and the heritability of both traits was fixed as 0.5. Genetic covariance and heritability were evenly distributed among all causal SNPs. When estimating local genetic covariance, we did not use information about causal SNP assignment and still fitted an (locally) infinitesimal model as proposed in SUPERGNOVA. The results are shown in the **Figure** below. SUPERGNOVA is robust to the sparse setting even when the model was mis-specified and achieved comparable results with a slight reduction in statistical power.

**Figure** (**Supplementary Figure 36** in the original submission). SUPERGNOVA is robust under misspecified models with sparse genetic architecture. **(A)** Local genetic covariance estimation. The red dashed lines represent true value of genetic covariance. **(B)** Type I error and statistical power **(C)** qq-plot of p value when the true local genetic covariance is set to be 0.

Next, we investigated the performance of SUPERGNOVA when the SNP effect size is affected by MAF which we denote as 𝑓B . Specifically, we assume the variance and covariance of the effect size of SNP 𝑗 are proportional to 𝑓B(1 − 𝑓B). That is to say

𝛽B~𝑁(0, ℎ" #[𝑓B(1 − 𝑓B)/𝜎]) and 𝛾B~𝑁(0, ℎ# #[𝑓B(1 − 𝑓B)/𝜎]), where 𝜎 = Σ𝑓B(1 − 𝑓B). And for the SNPs in the region of interest, 𝑐𝑜𝑣D𝛽B, 𝛾BE = [𝑓B(1 − 𝑓B)/𝜎I], where 𝜎I is the sum of 𝑓B(1 − 𝑓B) for all the SNPs in region 𝑖. This kind of assumption has been used in some literature[3]. The point estimates of local genetic covariance remained unbiased but we observed some inflation in the type-I error rate. This motivates us to propose a more general model that fits into different relation between MAF and genetic effects in our future work.

**Figure** (**Supplementary Figure 37** in the original submission). Under a mis-specified model assuming MAF-dependent genetic effects, local genetic covariance estimation remains unbiased but type I error shows some inflation. **(A)** Local genetic covariance estimation. The red dashed lines represent true value of genetic covariance. **(B)** Type I error and statistical power **(C)** qq-plot of p value when the true local genetic covariance is set to be 0.

Then, we simulated the effect sizes to be inversely proportional to LD score[4]. The results of point estimates are shown in **Supplementary Figure 38** (also see below). We did not observe any inflation in the type-I error rate. No replicates had p-values below 0.05 when the true genetic covariance was 0. There was substantial underestimation of local genetic covariance when the true genetic covariance was set to be 0.005 (see **Figure** below). Only one replicate had a p-value below 0.05. Overall, these results suggest that SUPERGNOVA may be conservative under a mis-specified model with LDdependent genetic effects.

**Figure** (**Supplementary Figure 38** in the original submission). SUPERGNOVA is not robust when per SNP genetic covariance is related with LD score. The red dashed lines represent true value of genetic covariance.

Finally, we studied how the sizes of local regions affect the performance of SUPERGNOVA. We started from the same region used in the simulations described in our original submission (chromosome 2: 176,998,822-180,334,969; 3.3Mb). Then, we expanded the size of the region to 6.8Mb (174,472,685-181,308,939), 8.8Mb (172,946,315-181,794,257), 12.2Mb (170,996,943-183,183,026), and 14.8Mb (169,568,977-184,356,242). We simulated data in two different scenarios to study the performance of SUPERGNOVA:

1) The effect sizes of the SNPs are correlated in the single region chr2: 176,998,822-180,334,969. The local genetic covariance is set to be 0.005. The genetic covariances of the SNPs outside the region are zero. This simulation setting represents a scenario where expanding the window size simply adds noise (with 0 genetic covariance) into the estimation.

2) The effect sizes of the SNPs are correlated in the entire chromosome 2. The global genetic covariance is set to be 0.25. This simulation setting mimics the genetic architecture where genetic covariance is ubiquitous in the genome. Under this scenario, expanding the window size will add to the total genetic covariance in the window. Similar to the simulation settings in our initial submission, SNPs on chromosome 2 were set as the background SNPs and the heritability values for both traits were fixed as 0.5.

We estimated the local genetic covariances of the regions with different sizes. The **Figure** below shows the trend of statistical power as the window size increases in two scenarios. We can observe that when the SNP effects are correlated locally, statistical power decreases as the window expands. When the SNP effects are correlated globally, the power increases as the window expands. However, we note that the argument about how to define window size is not entirely statistical. When two traits have highly polygenic genetic sharing, analysis based on a large window size will most likely improve statistical power. Still, smaller window size will help “fine-map” the local correlational patterns and identify precise genomic components that contribute to the shared genetic basis between complex traits. Our implemented SUPERGNOVA software allows users to specify the genomic region of interest and will estimate local genetic covariance accordingly.

**Figure** (**Supplementary Figure 39** in the original submission)**.** The power of local genetic covariance estimation for different window sizes. (**A**) Effect sizes of the SNPs are correlated only in a 3.3Mb region.

(**B**) Effect sizes of the SNPs are correlated globally.

We have added more details and clearer references to these additional analyses in the **Simulations** section in our revised manuscript.

**3. Line 100: The authors refer to the positive genetic correlation between ASD and cognitive ability as a paradox, but I believe there are epidemiological studies also reporting a positive phenotypic association. Can the authors provide more background information explaining why this positive genetic correlation is a paradox?**

We really appreciate this comment. For years, GWASs of ASD have failed to identify robust associations that can be consistently replicated, most likely due to ‘omnigenicity’, weak effect of common SNPs, and insufficient sample size. However, whole-exome sequencing (WES) studies for ASD have been fruitful, identifying numerous genes harboring loss-of-function *de novo* mutations and ultra-rare inherited variants[5-8]. The most recent WES study has increased the number of ASD risk genes to 102[5]. These findings have implicated multiple important pathways and biological processes involved in ASD etiology. One main story about ASD genes in these (rare variant) studies was that *de novo* mutations in ASD probands are enriched in certain pathways (e.g., chromatin modifiers) and these variants cause not only ASD but also a variety of neurodevelopmental disorders[6]. Comorbidity of ASD and intellectual disability in probands with *de novo* and ultra-rare variants shaped our understanding of ASD genetics until very recently. As the sample size grew, common SNP associations began to emerge in ASD GWAS. The GWAS dataset we used in our study was from Grove et al. 2019 which is the first GWAS that reported robust, significant, and replicable associations for ASD[9]. Large well-powered GWASs, coupled with methodological advances in multi-trait analysis, have led to exciting findings about the shared genetic basis of ASD and other genetically correlated traits. However, a finding that surprised many in the field was the highly significant genetic correlation between ASD and higher cognitive ability. This genetic correlation was first identified using relatively underpowered ASD GWAS[10], but has since been replicated in well-powered large studies[11] using various approaches [12]. This is why we described these observations as paradoxical – **WES tells us that genetics contribute to ASD and intellectual disability while GWAS suggests that genetics contribute to ASD and higher IQ**.

In this paper, we applied our new approach to estimate the local genetic covariance between ASD and cognitive ability and identified significant bidirectional correlations. Follow-up analyses further revealed that genomic regions showing opposite correlations were enriched for very different gene sets. Regions with negative correlations were in fact strongly enriched for chromatin modifier genes, suggesting that signals in ASD GWAS and WES may not be so different after all. However, genomic regions positively correlated between ASD and cognition hinted at additional ASD mechanisms not wellcharacterized in WES studies. Given the biological difference between two sets of genomic regions with opposite correlations between ASD and CP, an improved interpretation of the ‘paradox’ described above would be genetic heterogeneity. However, we note that it is the new results in our paper that illustrated different pathways’ heterogeneous effects. Before this, the positive genetic correlation of ASD and IQ has been a puzzling observation. Taken together, we believe that it is fair to describe the problem as a ‘paradox’. However, it is also completely fair to note that positive correlations between autism and cognition may have been identified in epidemiological studies. Here, our discussion focuses on the heterogenous roles of *genetics* in the shared etiology of autism and other disorders. We have highlighted related discussions in the **Discussion** section in our revised manuscript.

**4. Line 135: Figure 1 is very helpful but can the authors explain how sample overlap is distinguished from LD when decorrelating Z-statistics. Can the correction for overlap in Sample size be added to Figure 1?**

Thank you for your great suggestion. SUPERGNOVA needs an estimate of 9><? √9:9; (see **equation 3** in this manuscript) in order to correct for correlation of non-genetic effects of overlapping samples between two GWASs. We also put it here in the figure below.

**Figure** (**equation 3** in the manuscript). The covariance matrix of local z scores. We estimate local genetic covariance based on this equation.

As described in the **Local genetic covariance estimation** section on page 21 of our revised manuscript, we used the intercept of LDSC, i.e.,

𝐸d𝑧"B𝑧#Be𝑙Bf = √𝑛"𝑛#𝜌 𝑚 𝑙B + 𝜌3𝑛2 √𝑛"𝑛# to estimate 9><? √9:9;

.

We have highlighted the related sentences in the **Local genetic covariance estimation** section in our revised manuscript. We have also added this information in **Figure 1** of the revised manuscript.

**5. The primary simulation analysis are conducted using WTCCC data. Does the relatively small sample size limit the ability to simulate realistic GWAS scenario's?**

In **Section 4** of the **Supplementary Note** of our original submission, we had additional simulations which extended our simulation settings in the main text to densely imputed data from the UK Biobank (UKBB). We used phase 3 genotype data released by UKBB and restricted the analysis to autosomal variants with genotype missing rate < 0.01, imputation quality score > 0.3, Hardy-Weinberg equilibrium p-value > 1e-9, and minor allele frequency (MAF) > 0.1%. We also excluded SNPs not present in the 1000 Genomes (which is our LD reference panel). We randomly selected n=20,000 independent subjects with European ancestry for the simulation.

Similar to the **Simulation settings** section of the main text, SNPs on chromosome 2 were set as the background variants, and the heritability values for both traits were fixed as 0.5. There are 406,785 SNPs on chromosome 2 after quality control. We used the same region in the main text as the local region of interest (chromosome 2: 176,998,822- 180,334,969). There are 5,667 SNPs in this region. The covariance of the local genetic effects was set to be 0, 0.001, 0.002, 0.003, 0.004, and 0.005, respectively. We obtained highly consistent results in these new simulations. SUPERGNOVA achieved lower bias and improved statistical power with well-calibrated type I error. The density of SNPs did not show much influence on the performance of SUPERGNOVA.

The figure below shows the simulation results on UKBB genotype data, which is also the **Supplementary Figure 35** of our original submission. We described these details in the **Additional simulations** section in the **Supplementary Note** of our manuscript.

**Figure** (**Supplementary Figure 35** in the original submission). Comparison of the performance of SUPERGNOVA and 𝜌-HESS on UKBB data.

Since the main conclusion of the additional simulations on UKBB was consistent with the results of the simulations conducted on WTCCC, we only include the results on UKBB in the supplementary material. We have added clearer references to these results in the **Simulations** section of our revised manuscript.

**6. The authors conduct PRS analyses to further explore the influence of ASD-CP positive genetic correlation regions and ASD-CP negative genetic correlation regions. Although I find the question in itself interesting, the results of this specific section seem weak. The main problem is that the authors do not control the type-I error rate. For some analyses they use PRS percentiles, for other analyses they distinguish between extreme and non-extreme PRS scores. It is not always clear what the comparison group is. Some of the discussed findings are not even significant at P<0.05 and combined with a failure to control the familywise type I error rate, I was not convinced that these findings are robust. The authors should revise their approach, correct for multiple testing and better explain why they apply different PRS thresholds in the different analyses.**

We apologize for the lack of clarity on this matter in our original submission. Here, we provide more detailed rationales behind our analyses. The analyses described in **Figure 6** were designed to be a continuation of what was discussed in **Figures 4 and 5**. By applying SUPERGNOVA to GWAS of ASD and CP, we identified 24 genomic regions with significant local genetic covariance between two traits. Importantly, these identified regions showed bidirectional correlations between ASD and CP. These results clearly suggested that different pathways and biological processes were underlying the positive and negative genetic correlation of ASD and cognitive ability. It was reasonable to further investigate if these two sets of genetic components were associated with different clinical symptoms of ASD. This is what we presented in **Figure 6**. We built PRS+ and PRS- based on SNPs in these two sets of regions and tested their associations with IQ. Each proband has two PRSs constructed for ASD. PRS+ was constructed based on the regions where ASD and CP were positively correlated while PRS- was constructed based on the regions where ASD and CP were negatively correlated. We were expecting to see the probands with relatively high PRS+ should also have higher IQ than the probands with relatively high PRS-. So, all the comparisons in terms of IQ are between the groups of high PRS+ and high PRS-. We used PRS quantile to show that the gap of IQ between the groups of high PRS+ and PRS- groups increased with the quantile of high PRS+ and PRS- groups (**Figure 6A**). The probands in 99% quantile of PRS+ or PRS- were defined having “extreme” PRS+ or PRS- and we only compared the average IQ between the probands in 99% quantile of PRS+ and PRS-. Thank you for pointing this out, we have replaced all the “extreme” in this section by more rigorous description in our revised manuscript to avoid confusion. We only use 75% and 99% as the percentile threshold to perform statistical testing. 99% was the threshold for extreme PRS+/PRS- group and 75% was the baseline threshold because the statistics before 75% threshold were trivial. For example, if we used 0 as the threshold, all the probands would be in the same subgroup which is the complete set of all probands.

For other traits related to ASD, unlike IQ, we thought it would be more interesting to investigate the difference of these traits between high PRS+/PRS- groups with other probands who were not in the high percentile group of PRS+/PRS-. We acknowledge that the association results of PRS+\PRS- with clinical symptoms and ASD subtypes may not be statistically significant after multiple testing correction. We now put the figures and tables for these analyses in the supplementary material and discuss the limitations of these analyses in our **Discussion** section of the revised manuscript.

**7. Sentence 476: What is the test statistic of the overall test? What happens at percentiles below 75. Can this be added to the figure 6A?**

We used two-sample t-test to test the difference of IQ in the 99% percentile groups of PRS+ and PRS-. We have clarified this in the revised manuscript. The x-axis of **Figure 6A** in the original submission represents the percentile for PRS+/PRS-. The point ranges represent the means and standard errors of the IQ of the proband subgroups above the corresponding percentile for PRS+/PRS-. Consequentially, there are a substantial number of overlapping samples for the percentiles below 75 for PRS+ and PRS- and the differences of means and standard errors of IQ for percentiles PRS+ and PRS- below 75 of are trivial which are shown in the following figure.

**Figure.** Extension of Figure 6A in the original submission which include the percentiles below 75. Each interval indicates the standard error of the estimated mean.

**8. Line 484: please clarify, Figure 6C does not help and I did not understand this sentence.**

Thank you for your comment. As explained in our responses to comments 6 and 7, we constructed two PRSs for each proband (i.e., PRS+ and PRS-) based on genomic regions showing positive/negative local correlations between ASD and cognition. These two scores could be seen as the genetic load for ASD risk from two etiologically distinct pathways. The sentence referred to by the reviewer states that among the 19 ASD probands with very high PRS+ (above 99% percentile), no one had PRS- above the 90% percentile. This indicates that we did not observe any probands with extreme values of both PRS+ and PRS- which is consistent with the hypothesis that these two scores may represent different etiologic mechanisms underlying ASD. We have revised the sentence in the manuscript.

**9. There are some sentences that are difficult to read. E.g., lines 443-445; line 484**

Thank you for pointing this out. We have improved the texts in the revised manuscript.

**References**

1. Berisa, T. and J.K. Pickrell, *Approximately independent linkage disequilibrium blocks in*

*human populations.* Bioinformatics, 2016. **32**(2): p. 283-285.

2. Lu, Q., et al., *A Powerful Approach to Estimating Annotation-Stratified Genetic Covariance via GWAS Summary Statistics.* The American Journal of Human Genetics, 2017. **101**(6): p. 939-964.

3. Brown, B.C., et al., *Transethnic Genetic-Correlation Estimates from Summary Statistics.* American Journal of Human Genetics, 2016. **99**(1): p. 76-88.

4. Lee, J.J., et al., *The accuracy of LD Score regression as an estimator of confounding and genetic correlations in genome*-*wide association studies.* Genetic epidemiology, 2018. **42**(8): p. 783-795.

5. Satterstrom, F.K., et al., *Large-scale exome sequencing study implicates both developmental and functional changes in the neurobiology of autism.* Cell, 2020. **180**(3): p. 568-584. e23.

6. Iossifov, I., et al., *The contribution of de novo coding mutations to autism spectrum disorder.* Nature, 2014. **515**(7526): p. 216-U136.

7. Krumm, N., et al., *Excess of rare, inherited truncating mutations in autism.* Nat Genet, 2015. **47**(6): p. 582-8.

8. Sanders, S.J., et al., *De novo mutations revealed by whole-exome sequencing are strongly associated with autism.* Nature, 2012. **485**(7397): p. 237-41.

9. Grove, J., et al., *Identification of common genetic risk variants for autism spectrum disorder.* Nat Genet, 2019. **51**(3): p. 431-444.

10. Bulik-Sullivan, B., et al., *An atlas of genetic correlations across human diseases and traits.* Nature Genetics, 2015. **47**(11): p. 1236-+.

11. Anttila, V., et al., *Analysis of shared heritability in common disorders of the brain.* Science, 2018. **360**(6395): p. 1313-+.

12. Weiner, D.J., et al., *Polygenic transmission disequilibrium confirms that common and rare variation act additively to create risk for autism spectrum disorders.* Nature genetics, 2017. **49**(7): p. 978.

**2^nd^ round**

**Reviewer 1**

I thank the authors for having carefully and more than satisfactorily addressed my earlier questions with detailed explanations, and I congratulate the authors for an excellent paper!

**Reviewer 2**

Thank you for your very extensive responses to my earlier comments and for making the requested edits in the manuscript. I have no further questions or comments.
